# The myth of the De Geer Zone

**DOI:** 10.12688/openreseurope.16791.1

**Published:** 2024-01-03

**Authors:** Jean-Baptiste P. Koehl

**Affiliations:** 1 Department of Earth and Planetary Sciences, McGill University, Montreal, Québec, H3A 0E8, Canada; 2 Department of Geosciences, Universitetet i Oslo, Oslo, 0371, Norway

**Keywords:** Svalbard, Fram Strait, transform fault, thrust fault, shear zone, Cenozoic, De Geer Zone, Hornsund Fault Complex

## Abstract

**Background:**

Cenozoic rifting in the Arctic and the resulting opening of the Labrador Sea and the Fram Strait are typically associated with the movement of the Svalbard Archipelago c. 400 km southwards and its separation from Greenland. Thus far, most of this tectonic displacement was ascribed to lateral movement along the N–S-striking De Geer Zone, a thousand-kilometer-long paleo-transform fault believed to extend from northwestern Norway to northern Greenland.

**Methods:**

The study presents a new interpretation of tectonic structures on seismic reflection data north and west of Svalbard.

**Results:**

The present study reports the presence of two km-thick, hundreds of kilometers long, E–W- to WNW–ESE-striking shear zones, northwest and west of the island of Spitsbergen, Svalbard, in the Norwegian Arctic. Contractional structures within the shear zones, their strike, the inferred transport direction, and the great depth at which they are found indicate that they formed during the Timanian Orogeny in the late Neoproterozoic (c. 650–550 Ma). These structures extend at least 80–90 km west of the coastline of Spitsbergen. The presence of continuous, late Neoproterozoic Timanian thrusts this far west of Spitsbergen invalidates the occurrence of c. 400 km lateral movements along the N–S-striking De Geer Zone along the western Barents Sea–Svalbard margin in the Cenozoic.

**Conclusions:**

The present results suggest that the De Geer Zone does not exist and that related fault complexes (e.g., Hornsund Fault Complex) did not accommodate any strike-slip movement. In addition, the formation of major NW–SE-striking transform faults in the Fram Strait was controlled by Timanian thrust systems. The present results call for major revisions of all current plate tectonics models for the opening of the Fram Strait and Arctic tectonics in the Cenozoic and for critical reviews of major fault zones inferred from indirect observations.

## Plain language summary

Thus far, the Svalbard Archipelago and Greenland are thought to have started drifting away from one another during the opening of the Labrador Sea and subsequently of the Fram Strait in the last 60 million years. This resulted in the displacement of Svalbard c. 400 km to the south at its present location. Thus far, most of this lateral displacement was commonly believed to have occurred along the De Geer Zone, a major, N–S-striking, presently inactive fault that was proposed to run in a linear fashion along the coastline of western Svalbard and extending from northwestern Norway to northern Greenland. The present results indicate the presence of two, 650–550 million years old, several kilometers thick, hundreds of kilometers long, WNW–ESE-striking fault zones, which extend well past the speculated location of the <60 million years old De Geer Zone. The present study therefore suggest that the De Geer Zone does not exist and that the area’s tectonic history was largely controlled by WNW–ESE-striking fault zones, e.g., controlling the location of major active lateral fault zones in the Fram Strait (e.g., Molloy Fracture Zone). The study also suggests a need for a revision of the nomenclature related to fault zones.

## Introduction

The De Geer Zone is a major structural element of the west Spitsbergen transform margin that is believed to have accommodated 400 kilometers of dextral strike-slip movement during the opening of the Northeast Atlantic and Arctic oceans and of the Fram Strait in the Cenozoic ( De Geer, 1926; du Toit, 1937; Faleide *et al*., 1993; Faleide *et al*., 2008; Harland, 1961; Harland, 1967; Harland, 1969; Horsfield & Maton, 1970; Piepjohn *et al*., 2016; Wegmann, 1948), thus facilitating the movement of Svalbard towards the south. Despite numerous studies both onshore and offshore along the western Svalbard margin, the actual trace of the De Geer Zone remains a matter of debate ( Bergh & Grogan, 2003; Geissler & Jokat, 2004; Myhre *et al*., 1982; Vogt *et al*., 1981). This is notably related to the striking lack of evidence of lateral movement on seismic datasets (e.g., Austegard *et al*., 1988; Eiken, 1994; Gabrielsen *et al*., 1990; Lasabuda *et al*., 2018; Riis & Vollset, 1988), apart from a few evidence of minor sinistral strike-slip displacement ( Eiken & Austegard, 1987), which markedly contrast with the inferred, dominant dextral component of movement along the De Geer Zone ( du Toit, 1937; Faleide *et al*., 1993; Harland, 1961; Harland, 1967; Harland, 1969; Horsfield & Maton, 1970; Lepvrier, 1990; Lepvrier & Geyssand, 1985; Steel & Worsley, 1984; Steel *et al*., 1981; Steel *et al*.,1985; Wegmann, 1948).

The present study targets two E–W- to WNW–ESE-striking structures northwest and west of Spitsbergen, respectively the Risen and Kinnhøgda–Daudbjørnpynten fault zones, which extend c. 80–90 km west of the coastline of Spitsbergen. The structures appear on several, NNW–SSE- to NNE–SSW- as well as E–W-oriented 2D seismic lines. The present contribution describes their overall geometry and that of minor internal structures. The latter are further discussed to resolve the kinematics and reactivation history of the main shear zones. The geometry and kinematics were then used to infer the possible timing of formation of the structures. The results have major implications for the opening of the Fram Strait, fault kinematics along sheared margins (e.g., western Barents Sea–Svalbard margin) and for the interpretation of major paleo-transform faults (e.g., De Geer Zone).

The results of the present study suggest that all the current plate tectonics models for the opening of the Fram Strait should be updated with new fault lines and kinematics. In addition, the study calls for a serious reconsideration of all major faults inferred from indirect observations, generally as necessities to make up for paleogeographic reconstructions shortcomings, rather than observed on specific datasets (e.g., Wegener Fault). A methodology should be set for the classification of major faults to clearly distinguish tentative faults (e.g., Wegener Fault and De Geer Zone), e.g., by calling them “lineaments” or “zones” instead of “faults”, from beyond-reasonable-doubts faults (e.g., San Andreas fault – Crowell, 1979; Grant Ludwig *et al*., 2019; Molnar & Atwater, 1973; Huffman, 1972; – and Timanian thrusts systems in the Norwegian Barents Sea and Fram Strait – Klitzke *et al*., 2019; Koehl, 2020; Koehl *et al*., 2022a; Koehl *et al*., 2023a). Another implication of the present study is the need to adopt an interdisciplinary approach when mapping and interpreting major faults, including at least some regional (e.g., geophysical) datasets, rather than using exclusively local fieldwork data.

### Geological setting

**Figure 1.**
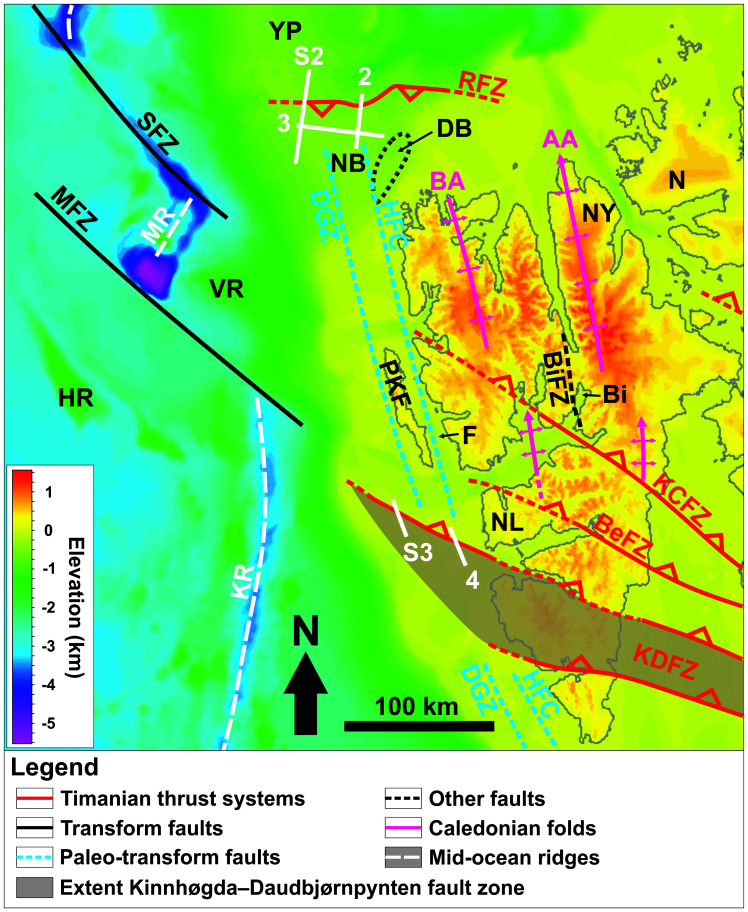
Location of the study area north and northwest of Spitsbergen. The white lines show the location of the seismic sections discussed. The topographic–bathymetry map is from Jakobsson *et al*. (2012). The geology of the area is from Gee and Hjelle (1966), Johnson and Eckhoff (1966), Faleide *et al*. (1993), Myhre and Thiede (1995), Bergh *et al*. (1997), Witt-Nilsson *et al*. (1998), Bergh and Grogan (2003), Blinova *et al*. (2013), Braathen *et al*. (2018), Koehl and Allaart (2021), Kristoffersen *et al*. (2020), Koehl *et al*. (2022a). Abbreviations: AA: Atomfjella Antiform; BA: Bockfjorden Anticline; BeFZ: Bellsundbanken fault zone; Bi: Billefjorden; BiFZ: Billefjorden Fault Zone; DB: Danskøya Basin; DGZ: De Geer Zone; F: Forlandsundet; HFC: Hornsund Fault Complex; HR: Hovgård Ridge; KCFZ: Kongsfjorden–Cowanodden fault zone; KDFZ: Kinnhøgda–Daudbjørnpynten fault zone; KR: Knipovich Ridge; MFZ: Molloy Fracture Zone; MR: Molloy Ridge; N: Nordaustlandet; NB: Nansen Bank; NF: Ny-Friesland; NL: Nordenskiöld Land; PKF: Prins Karls Forland; RFZ: Risen fault zone; SFZ: Spitsbergen Fracture Zone; VR: Vestnesa Ridge; YP: Yermak Plateau.

***Timanian Orogeny*.** The Timanian Orogeny is a major episode of overall top-SSW, late Neoproterozoic (650–550 Ma) contraction, during which continental lithosphere formed in the Arctic (e.g., Estrada *et al*., 2018a; Estrada *et al*., 2018b; Gee *et al*., 2000; Pease *et al*., 2004; Rekant *et al*., 2019; Rosa *et al*., 2016). This tectonic episode was initially thought to be restricted to northeastern Norway ( Dallmeyer & Reuter, 1989; Gorokhov *et al*., 2001; Siedlecka, 1975; Siedlecka & Siedlecki, 1971) and northwestern Russia ( Kostyuchenko *et al*., 2006; Kuznetsov *et al*., 2007; Larionov *et al*., 2004; Lorenz *et al*., 2004; Olovyanishnikov *et al*., 2000; Pease *et al*., 2004; Remizov, 2006; Remizov & Pease, 2004). However, recent studies in Greenland ( Estrada *et al*., 2018a; Rosa *et al*., 2016), Arctic Canada ( Estrada *et al*., 2018b), the Lomonosov Ridge ( Rekant *et al*., 2019), Svalbard ( Dallmeyer *et al*., 1990a; Faehnrich *et al*., 2020; Koglin *et al*., 2022; Majka *et al*., 2008; Majka *et al*., 2012; Manecki *et al*., 1998; Peucat *et al*., 1989), and the Barents Sea ( Klitzke *et al*., 2019; Koehl, 2020; Koehl *et al*., 2022a; Koehl *et al*., 2023a) show that the Timanian Orogeny extends over a much broader area ( Figure 1). These findings also indicate that the Svalbard Archipelago and the Barents Sea were already accreted to northern Norway in the late Neoproterozoic ( Koehl, 2020; Koehl *et al*., 2022a; Koehl *et al*., 2023a).

Most Timanian structures strike WNW–ESE to E–W and consist of asymmetric folds and mylonitic brittle–ductile thrusts and shear zones. These structures were later reworked into dome- and trough-shaped folds during Caledonian contraction, and reactivated and/or overprinted during Devonian–Carboniferous extension, early Cenozoic Eurekan contraction, and late Cenozoic rifting ( Faehnrich *et al*., 2020; Gabrielsen *et al*., 2022; Koehl & Rimando, 2023 submitted; Koehl *et al*., 2022a; Koehl *et al*., 2023a; Siedlecka & Siedlecki, 1971).

A structure of particular interest is the Kinnhøgda–Daudbjørnpynten fault zone, a 60 km wide, hundreds of kilometers long thrust system, which extends from the northern Barents Sea to Wedel Jarlsberg Land in southwestern Spitsbergen ( Figure 1). There, the Vimsodden–Kosibapasset Shear Zone ( Mazur *et al*., 2009) is believed to represent the onshore continuation of the southern edge of the thrust system ( Koehl *et al*., 2022a).

***Caledonian Orogeny*.** Caledonian contraction resulted in the formation of N–S-striking fabrics and structures, both in Svalbard and the Barents Sea ( Birkenmajer, 1975; Birkenmajer, 2004; Braathen *et al*., 1999; Dumais & Brönner, 2020; Gernigon *et al*., 2014; Gudlaugsson *et al*., 1987; Gudlaugsson *et al*., 1998; Hjelle, 1979; Johansson *et al*., 2004; Johansson *et al*., 2005; Koehl *et al*., 2022a; Koehl *et al*., 2023a; Manby, 1986; Witt-Nilsson *et al*., 1998). Major structures include a well-developed foliation ( Gee *et al*., 1992; Witt-Nilsson *et al*., 1998), brittle–ductile thrusts ( Birkenmajer, 1975; Birkenmajer, 2004; Manby, 1986), tens of kilometers wide, gently north-plunging folds and antiformal (thrust) stacks ( Dumais & Brönner, 2020; Gee & Hjelle, 1966; Witt-Nilsson *et al*., 1998; Figure 1), and blueschist and eclogite metamorphism ( Dallmeyer *et al*., 1990b; Horsfield, 1972; Ohta *et al*., 1995). In northwestern Spitsbergen (i.e., closest to the study area), basement rocks consist of Grenvillian metasedimentary and metaigneous rocks, which were later reworked and intruded by granitic plutons during the Caledonian Orogeny ( Gee & Hjelle, 1966; Hjelle, 1979; Myhre *et al*., 2008; Pettersson *et al*., 2009a; Pettersson *et al*., 2009b).

***Devonian–Carboniferous extension*.** Late- to post-orogenic extensional collapse of the Caledonides resulted in the deposition of thick (c. 9–10 km thick) Devonian sedimentary rocks ( Dallmann & Piepjohn, 2020; Friend, 1961; Friend *et al*., 1997; Friend & Moody-Stuart, 1972; Gernigon *et al*., 2014; Murascov & Mokin, 1979) along low-angle detachments ( Braathen *et al*., 2018; Chorowicz, 1992; Koehl *et al*., 2018; Maher *et al*., 2022; Roy, 2007; Roy, 2009) in northern Spitsbergen. In the Carboniferous, extension slowed down, and kilometre-thick sedimentary rocks of the Billefjorden and Gipsdalen groups were deposited in subsiding basins, both along inherited Timanian and Caledonian fabrics ( Cutbill & Challinor, 1965; Cutbill *et al*., 1976; Koehl *et al*., 2018; Koehl-Muñoz-Barrera, 2018; McCann & Dallmann, 1996; Samuelsberg *et al*., 2003; Smyrak-Sikora *et al*., 2018). Note that the Late Devonian Svalbardian Orogeny is now thought not to have occurred in Svalbard and will therefore not be discussed in the present contribution ( Koehl *et al*., 2022b and references therein).

***Early Cenozoic Eurekan contraction*.** In the Paleocene, the opening of the Labrador Sea and possibly of Baffin Bay was accompanied by an episode of contraction in northern Greenland and western Spitsbergen, which resulted in the formation of the West Spitsbergen Fold-and-Thrust Belt ( Dallmann *et al*., 1993; Gion *et al*., 2017; Jones *et al*., 2017; Oakey & Chalmers, 2012). Major folds and thrusts in the belt strike N–S to NNW–SSE, i.e., parallel to the coastline in western Spitsbergen ( Bergh & Grogan, 2003; Dallmann *et al*., 1993; Lyberis & Manby, 2001; Maher, 1988; Maher *et al*., 1986; Maher *et al*., 1989; Maher *et al*., 1997; Manby, 1986; Manby & Lyberis, 2001a; Manby & Lyberis, 2001b; Tessensohn *et al*., 2001a; Tessensohn *et al*., 2001b; Tessensohn *et al*., 2001c; von Gosen & Piepjohn, 2001; Welbon & Maher, 1992).

Contraction faded as rifting and seafloor spreading initiated in the northeastern Atlantic and Arctic oceans at ca. 56 Ma near the Paleocene–Eocene boundary. Svalbard and Greenland are then believed to have gradually sled past one another along a major paleo-transform fault, the De Geer Zone, which is thought to have accommodated c. 400 km of dextral strike-slip movement prior to breakup in the Fram Strait ( du Toit, 1937; Faleide *et al*., 1993; Harland, 1961; Harland, 1967; Harland, 1969; Horsfield & Maton, 1970; Piepjohn *et al*., 2016; Wegmann, 1948). The main segment of the De Geer Zone, the Hornsund Fault Complex, was mapped on seismic data as a series of steep, east- and west-dipping faults bounding N–S-trending basement ridges, e.g., Nansen Bank in the west, from sedimentary basins, e.g., the Danskøya Basin in the east ( Figure 1; Austegard *et al*., 1988; Bergh & Grogan, 2003; Eiken, 1994; Eiken & Austegard, 1987; Faleide *et al*., 1993; Gabrielsen *et al*., 1990; Geissler & Jokat, 2004; Grogan *et al*., 1999; Mann & Townsend, 1989; Myhre *et al*., 1982; Riis & Vollset, 1988; Vogt *et al*., 1981). However, indicators of strike-slip movements along the De Geer Zone and Hornsund Fault Complex are difficult to identify and are thought to have been overprinted by later extensional structures, which dominate the margin at present.

According to previous studies, the De Geer Zone and Hornsund Fault Complex are either one and the same structure (e.g., Bergh & Grogan, 2003; Faleide *et al*., 1993) or discrete structures (e.g., Kristoffersen *et al*., 2020; Piepjohn *et al*., 2016; Figure 1). However, should they be separate structures, there is no direct evidence of the western one (the De Geer Zone). Nevertheless, the lack of evidence supporting strike-slip movement along the Hornsund Fault Complex ( Austegard *et al*., 1988; Eiken, 1994; Riis & Vollset, 1988) generates a need for an extra tentative (yet to be observed) fault zone to the west, farther offshore.

***Late Cenozoic rifting*.** Breakup in the Fram Strait may have initiated at ca. 24 Ma (Chron 7; Engen *et al*., 2008), i.e., much later than the northeastern Atlantic and the Arctic oceans (at ca. 56 Ma; Faleide *et al*., 1993; Faleide *et al*., 2008). From then, transform movements are thought to have been accommodated by two, c. 200 km long transform faults, the Molloy and Spitsbergen fracture zones ( Crane *et al*., 1982; Johnson & Eckhoff, 1966; Myhre & Thiede, 1995; Thiede *et al*., 1990). At that time, N–S-striking faults such as the Hornsund Fault Complex were reactivated as normal faults, developed a listric geometry, and accommodated the deposition of thick mid–upper Cenozoic (Oligocene–?) Miocene–Quaternary sediments (e.g., Danskøya Basin; Eiken, 1993; Eiken, 1994; Geissler & Jokat, 2004; Geissler *et al*., 2011). The Forlandsundet Graben is generally thought to have formed during this stage although accurate age constraints for sediment deposition are still lacking ( Livshits, 1992; Manby, 1986; Schaaf *et al*., 2020). In addition, the relationship of the graben sediments with adjacent basement rocks (not necessarily faulted) indicates that a formation during the Eurekan event might be possible too ( Gabrielsen *et al*., 1992; Kleinspehn & Teyssier, 1992; Kleinspehn & Teyssier, 2016; Lepvrier, 1990). Rifting was accompanied by magmatism in the Miocene as documented by lava flows onshore northern Svalbard ( Prestvik, 1978; Skjelkvåle *et al*., 1989).

## Methods

Two-Way Time (TWT) 2D seismic data from the Norwegian National Data Repository for Petroleum Data (DISKOS database) and of the University of Bergen around Spitsbergen were analyzed to map a basement-seated structure west of Spitsbergen (see Extended data: Supplement S1 for an overview of the database used ( Koehl, 2023b)). Petrel (version 2021.3) was used to interpret the data, which may also be interpreted via OpendTect, a free open-source alternative software. The figures were designed using CorelDraw 2017 ( GIMP is a freely available open-source alternative). High-resolution versions of the figures and supplements are available on DataverseNO ( Koehl, 2023a; https://doi.org/10.18710/J98MLA). These are necessary to observe the described structures in their full resolution. Additional seismic sections are also available online as electronic supplements on DataverseNO ( Koehl, 2023b; https://doi.org/10.18710/KUQNII).

To interpret the data, our descriptions were compared to previous seismic studies around the Svalbard Archipelago and the Barents Sea and onshore field studies in Svalbard, as well as to other studies of seismic reflection data worldwide. Noteworthy, in order to be as conservative as possible, it was assumed that the De Geer Zone and the Hornsund Fault Complex are discrete structures, i.e., that the De Geer Zone might be located west of Prins Karls Forland although no tangible evidence supporting this has been found thus far ( Figure 1). This implies that the hereby drawn conclusions would also be valid, if not more resounding, should these two structures be one.

When analyzing seismic data, numerous examples of seismic artifacts were identified. These are indicated on the interpreted seismic sections but are not further discussed as they are not the focus of the present work. Notable artifacts include multiples, diffraction, and bottom-simulating reflections.

Phanerozoic sedimentary successions north of Spitsbergen were previously studied and extensively described by previous studies (among others, Eiken, 1993; Eiken, 1994; Eiken & Austegard, 1987; Geissler & Jokat, 2004) and are therefore not mentioned because they are out of scope of the present contribution.

## Results

### Description

**Figure 2.**
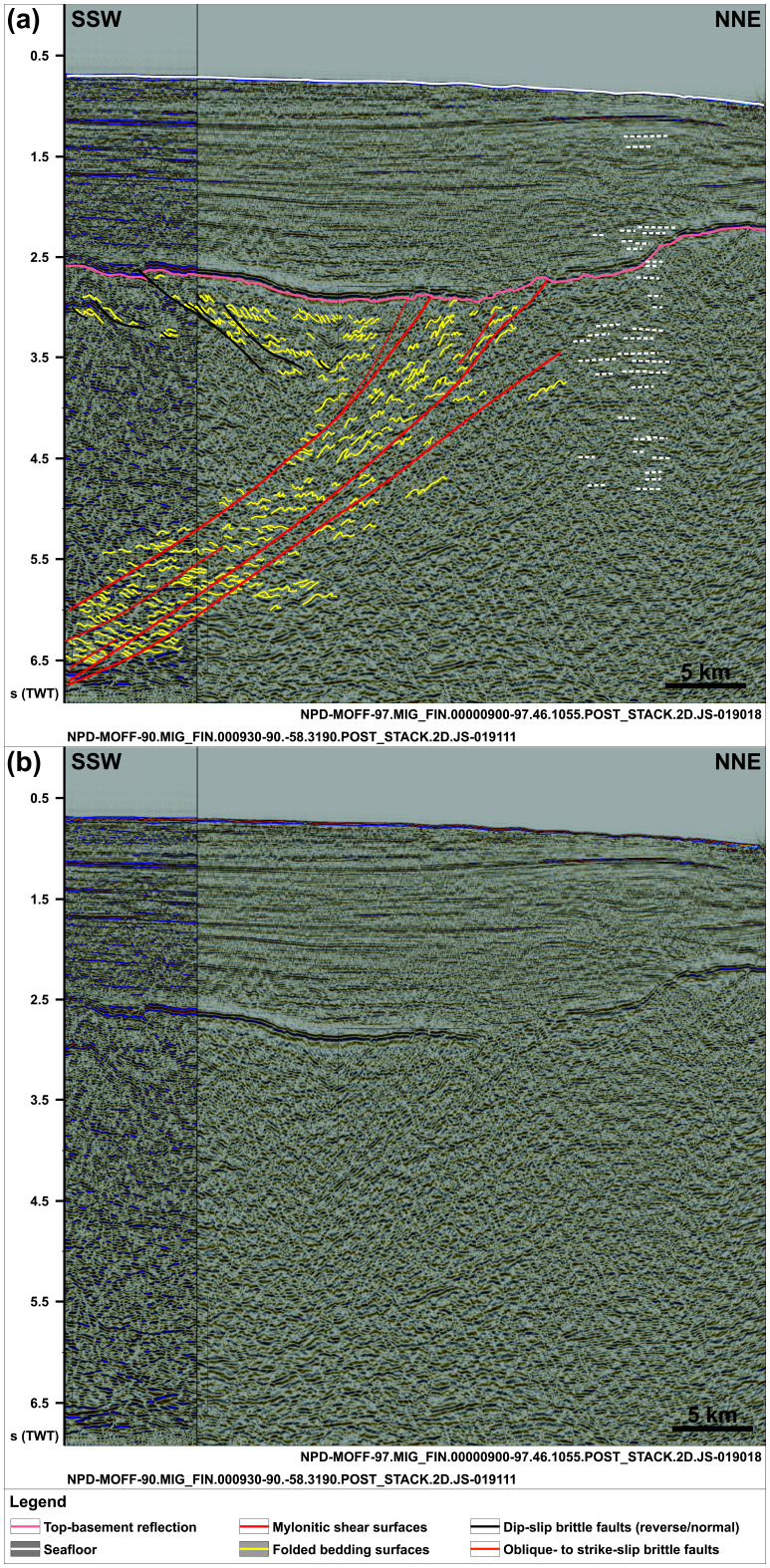
( **a**) Interpreted and ( **b**) uninterpreted N–S-oriented seismic section showing the south-dipping geometry of the Risen fault zone and that of internal north-verging folds, extensional duplexes, and mylonitic shear surfaces. Notice the reverse offset of the Top-basement reflection by a minor top-south thrust in the south and a number of seismic artifacts just north of the Risen fault zone (dashed white lines). Location is shown in Figure 1.

**Figure 3.**
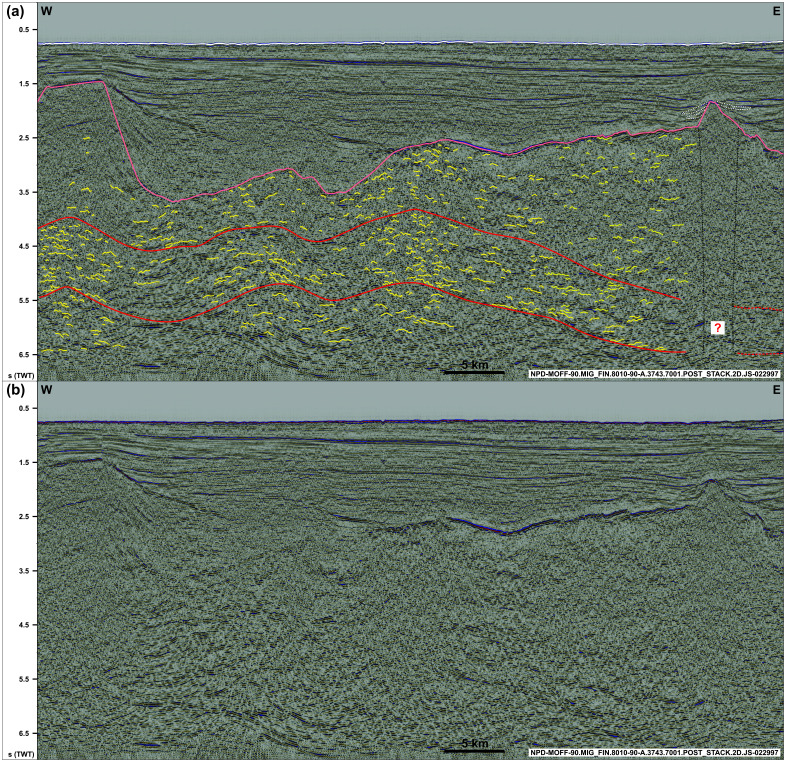
( **a**) Interpreted and ( **b**) uninterpreted along-strike seismic section showing the undulating, gently folded geometry of the Risen fault zone and related basement structures within and around the shear zone (dominantly symmetric open folds). Notice the washed-out zone below the conical ridge in the east (dotted black lines), which is possibly related to magmatic intrusions below a volcanic cone and associated lava flows (dotted white lines). See location in Figure 1 and legend in Figure 2.

**Figure 4.**
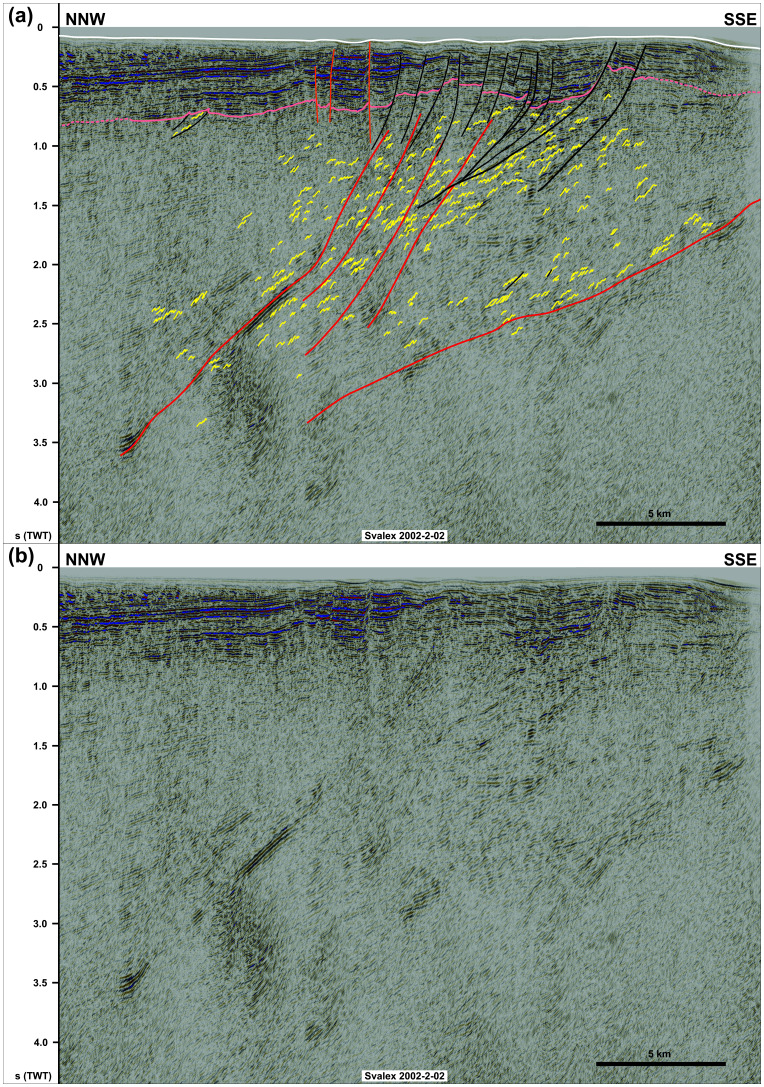
( **a**) Interpreted and ( **b**) uninterpreted NNW–SSE seismic section showing the northern flank of the NNE-dipping Kinnhøgda–Daudbjørnpynten fault zone and that of internal south-verging folds, duplexes, and mylonitic shear surfaces. Notice the steeply to moderately NNE-dipping geometry of the shallow brittle faults in the south indicating mostly dip-slip kinematics, whereas shallow brittle over the northernmost edge of the Kinnhøgda–Daudbjørnpynten fault zone in the north are subvertical thus indicating a strike-slip component.

**Figure 5.**
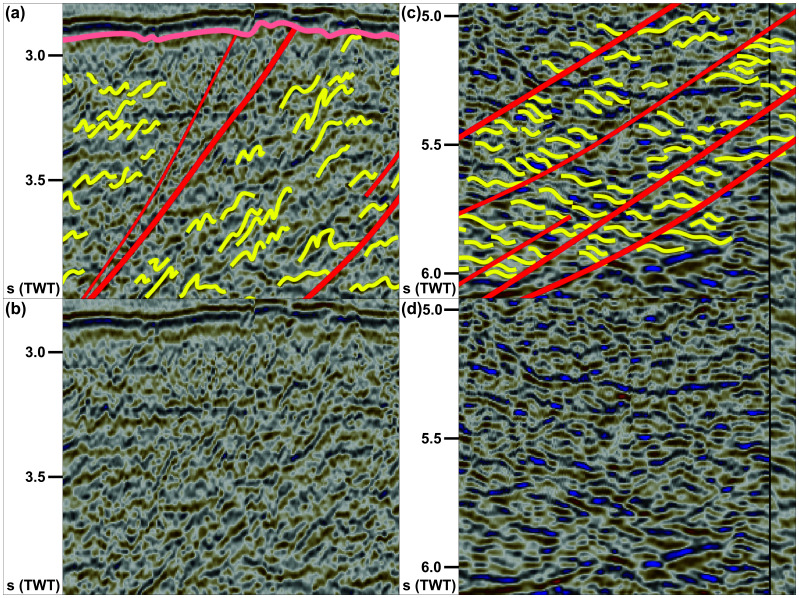
( **a**) Interpreted and ( **b**) uninterpreted zoom in the upper part of the Risen fault zone consisting of asymmetric, north-verging folds and mylonitic surfaces. Notice the rugose morphology of the Top-basement reflection above the Risen fault zone. ( **c**) Interpreted and ( **d**) uninterpreted zoom in the lower part of the Risen fault zone showing down-south extensional duplexes separated by mylonitic shear surfaces. See location and legend in Figure 2.

Seismic reflection data reveal the occurrence of two seismic packages of interest displaying moderate-amplitude, asymmetric seismic reflections separated by linear disruption surfaces within basement rocks north and west of Spitsbergen ( Figure 2, Figure 3, and Figure 4). The package north of Spitsbergen is 5–8 km wide and 1.0–1.5 second (TWT) thick and shows reflections and disruption surfaces dipping dominantly to the south ( Figure 2). The package west of Nordenskiöld Land ( Figure 1) is 5–12 km wide, 1.0–2.5 second (TWT) thick and displays a general dip to the north-northeast ( Figure 4). The package of south-dipping reflections extends from a depth of ca. 2.5 (locally 2.0) seconds up to 9.0 seconds in the west (TWT) and is bounded by two prominent disruption surfaces that truncate adjacent gently undulating basement reflections ( Figure 2 and Figure 5a–b and Supplement S2 ( Koehl, 2023a)). The package west of Nordenskiöld Land extends from a minimum depth of 0.5 second (TWT) just west of the coast to a depth of at least 5.5 second (TWT) in the west ( Figure 4 and Supplement S3 ( Koehl, 2023a)).

In N–S- to NNW–SSE-oriented seismic cross sections, the upper part of the package north of Spitsbergen (ca. 2.5 to 5.0 seconds TWT) and the entirety of the package west of Nordenskiöld Land show strongly curving, typically a few hundreds of meters wide reflections with large (curving) amplitude. Within both packages, the strongly curving reflections show a pronounced asymmetry. North of Spitsbergen, the reflections consist dominantly of long and gently dipping southern limbs and of narrower, more steeply dipping northern limbs as if “leaning” towards the north ( Figure 2 and Figure 5a–b and Supplement S2 ( Koehl, 2023a)). The opposite is true for the package west of Nordenskiöld Land, where asymmetric curving reflections show elongated, gently dipping northern limbs and shorter, steeply dipping southern limbs ( Figure 4 and Supplement S3 ( Koehl, 2023a)). Above the described packages, the Top-basement reflection displays a rugose geometry, which differs from its generally smooth character elsewhere ( Figure 2 and Figure 4). The lower part of the package of south-dipping reflections in the north (below 5.0 seconds TWT) displays dominantly Z-shaped reflections (yellow lines in Figure 5c–d) occurring in elongated aggregates, which parallel and are separated from one another by major disruption surfaces (red lines in Figure 2 and Figure 5c–d). The package west of Nordenskiöld Land also displays Z-shaped reflections in places (Supplement S3 ( Koehl, 2023a)), but reflections with S-shaped geometries are also observed ( Figure 4).

**Figure 6.**
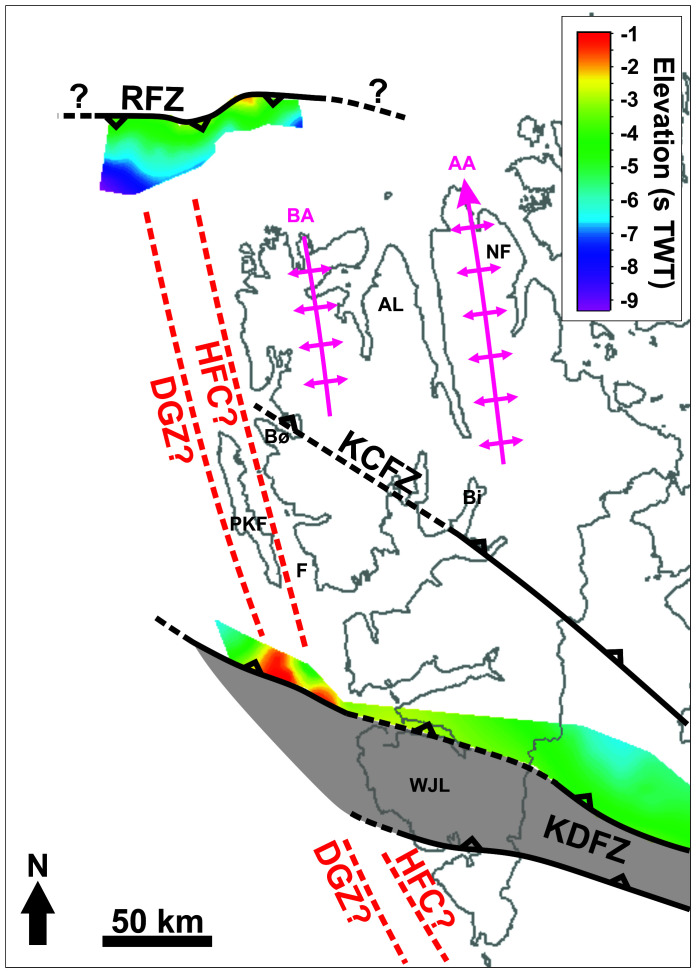
Depth map (in seconds TWT) of the south-dipping Risen fault zone lower envelope. The map shows that the Risen and Kinnhøgda–Daudbjørnpynten fault zones extend well across and past the location of the De Geer Zone and Hornsund Fault Complex west of Spitsbergen. Notice the similar strike and width of the south-plunging folds of the Risen fault zone and of the north-plunging fold of the Kinnhøgda–Daudbjørnpynten fault zone west of Spitsbergen to that of major Caledonian fold structures onshore Spitsbergen (e.g., Bockfjorden Anticline and Atomfjella Antiform). Abbreviations: AA: Atomfjella Antiform; AL: Andrée Land; BA: Bockfjorden Anticline; Bi: Billefjorden; Bø: Brøggerhalvøya: DGZ: De Geer Zone; F: Forlandsundet; HFC: Hornsund Fault Complex; KCFZ: Kongsfjorden–Cowanodden fault zone; KDFZ: Kinnhøgda–Daudbjørnpynten fault zone; NF: Ny-Friesland; PKF: Prins Karls Forland; RFZ: Risen fault zone; WJL: Wedel Jarlsberg Land.

In E–W-oriented (along-strike) seismic sections, both packages display a gently undulating geometry with a wavelength of c. 10–20 km (e.g., Figure 3). Internal reflections also show gently undulating, open, and rather symmetric geometries (locally slightly asymmetric) with wavelengths in the range of 0.5–1.0 km ( Figure 3). The overall undulating geometry of the two major packages also appears on the depth map, which shows that the 10–15 km wide folds are characterized by a south-plunging geometry for the package northwest of Spitsbergen and by a northern plunge for the package west of Nordenskiöld Land ( Figure 6). Both packages also shows a gently undulating geometry in map view and pinch out below the Top-basement reflection in places, with a dominant E–W strike alternating with ENE–WSW and WNW–ESE strikes locally for the northern package, and alternating WNW–ESE- and E–W-striking segments for the package west of Nordenskiöld Land ( Figure 1 and Figure 6).

Seismic cross sections also show the presence of asymmetric curving reflections in basement rocks adjacent to both packages at depth of 2.5 to 3.5 seconds (TWT) north of Spitsbergen ( Figure 2) and 0.7–3.0 seconds (TWT) west of Nordenskiöld Land ( Figure 4). In the north, a notable difference is the opposite vergence of the reflections, i.e., “southward-leaning” with large, gently dipping northern limbs and short, steeply dipping southern limbs ( Figure 2). These reflections are crosscut by gently north-dipping disruption surfaces across which they are offset and may, in places, be correlated with their offset counterpart. One of these disruption surfaces extends across the Top-basement reflection, showing a minor (a few hundreds of meters) top-south reverse offset, but does not extend into overlying seismic reflections. By contrast, several shallow disruption surfaces are seen to crosscut the Top-basement surface west of Nordenskiöld Land ( Figure 4). Above the major NNE-dipping package, these surfaces show steeply to moderately NNE-dipping, listric geometries, merge at depth with major disruption surfaces within the NNE-dipping package and are associated with reverse and normal offsets of the Top-basement reflection in the south (black lines in Figure 4). Just north of the major package, a few subvertical disruption surfaces are associated with narrow, triangular uplifts and minor vertical offsets of the Top-basement reflections (orange lines in Figure 4). Most of these disruption surfaces die out ca. 0.1 second (TWT) below the seafloor reflection except for one subvertical surface, which extends all the way to the seafloor ( Figure 4). Both the listric and the subvertical disruption surfaces correlate with gentle open folding of the seafloor ( Figure 4).

North of Spitsbergen, the southeastern part of the package of south-dipping reflections is seemingly crosscut by a zone with subvertical disruptions below a 3–5 km wide conical ridge (dotted black lines in Figure 3). Above the Top-basement reflection, the ridge is associated with ca. 0.1 second thick packages of moderate amplitude reflections, which pinch out within 5 km from the ridge giving the ridge and the related pinching out packages a Christmas-tree geometry (dotted white lines in Figure 3).

### Interpretation

The asymmetric and curving reflections in the upper part of the package of south-dipping reflections north of Spitsbergen in seismic cross sections are interpreted as tightly folded bedding surfaces in Precambrian–early Paleozoic metasedimentary rocks ( Figure 2 and Figure 5a–b). The “northward-leaning” geometry of individual reflections suggests that they correspond to north-verging folds reflecting top-north thrusting ( Figure 2 and Figure 5a–b and Supplement S2). Conversely, “southward-leaning” asymmetric reflections within the major NNE-dipping package west of Nordenskiöld Land are interpreted as SSW-verging folds ( Figure 4 and Supplement S3). The interpretation is supported by previous seismic studies in the Barents Sea reporting similar reflection geometries ( Koehl, 2020; Koehl *et al*., 2022a; Koehl *et al*., 2023a).

The S- and Z-shaped reflections in the lower part of the package of south-dipping reflections in the north and locally in the NNE-dipping package west of Nordenkiöld Land are interpreted as tightly folded bedding surfaces in metasedimentary rocks offset and stacked onto one another by minor brittle faults ( Figure 2 and Figure 5c–d). The resulting aggregates of S- and Z-shaped reflections are interpreted as duplex structures ( Boyer & Elliott, 1982; McClay, 1992). Such geometries are not unusual for pre-Caledonian basement rocks in the Barents Sea ( Koehl, 2020; Koehl *et al*., 2022a; Koehl *et al*., 2023a) and onshore Svalbard ( Bergh *et al*., 1997; Bergh *et al*., 2000; Braathen *et al*., 1999). The dominant Z-like shape for reflection aggregates in the lower part of the south-dipping package north of Spitsbergen and locally within the NNE-dipping package west of Nordenskiöld Land suggest a down-south and down-NNE component of extensional movement respectively ( Figure 2 and Supplement S3). On the contrary, S-shaped reflections within the package west of Nordenskiöld Land suggest top-SSW contractional movement ( Figure 4).

 Both the disruption surfaces bounding and within the two major packages truncate the interpreted duplexes and asymmetric folds. They are therefore interpreted as major faults ( Figure 2). Furthermore, the rugose geometry of the Top-basement reflection above these major packages indicates differential erosion of basement rocks within the two packages ( Figure 2 and Figure 4). This suggests the occurrence of significant rheological contrasts within the packages. A probable cause may be the presence of relatively strong mylonitic shear zones around major faults and slip surfaces alternating with weaker zones of non- to less-mylonitic zones (e.g., Fountain *et al*., 1984; Hurich *et al*., 1985), i.e., reflecting strain partitioning within a major shear zone. Thus, the 5–12 km wide packages are interpreted as major south- and NNE-dipping shear zones. This interpretation is consistent with basement subcrops above shear zones and with the geometry of major shear zone elsewhere ( Collanega *et al*., 2019; Fazlikhani *et al*., 2017; Fountain *et al*., 1984; Koehl *et al*., 2018; Koehl *et al*., 2022a; Koehl *et al*., 2023a; Lenhart *et al*., 2019; Phillips & McCaffrey, 2019; Phillips *et al*., 2016; Phillips *et al*., 2019). The package north of Spitsbergen is hereby named the Risen fault zone. The package west of Nordenskiöld Land is interpreted as the continuation of the northern flank of the Kinnhøgda–Daudbjørnpynten fault zone. This is supported by the alignment of the shear zone location and matching strike, dip and geometry with the northern edge of the Kinnhøgda–Daudbjørnpynten fault zone in Storfjorden just east of southern Spitsbergen ( Koehl *et al*., 2022a; Figure 1). As a result, the Kinnhøgda–Daudbjørnpynten fault zone is now believed to extend the entire width (along a N–S axis) of Wedel Jarlsberg Land ( Figure 1).

 Comparably, asymmetric, “southward-leaning” reflections within shallow basement rocks (depth of 2.5–3.5 seconds TWT) and truncating disruption surfaces south of the south-dipping shear zone north of Spitsbergen are interpreted as south-verging folds and top-south brittle (–ductile?) thrusts ( Figure 2). The truncation of the Top-basement reflection by the largest top-south thrust suggests a reactivation of this thrust during a subsequent event of contraction, possibly in the early Cenozoic during the Eurekan event as suggested by its truncation of the Top-basement reflection but not of overlying upper Cenozoic sedimentary strata ( Figure 2). Alternatively, this thrust might be younger than all the surrounding structures, but this is considered highly unlikely because the strong rheological discontinuities at and around the Risen fault zone and other north-dipping thrusts would certainly have been reactivated or overprinted. This would have probably resulted in the truncation of the Top-basement reflection elsewhere prior to the formation of a brand-new thrust.

 West of Nordenskiöld Land, the shallow disruption surfaces are interpreted as Cenozoic brittle faults because they crosscut overlying, probably lower Cenozoic ( Blinova *et al*., 2009; Gabrielsen *et al*., 1992) sedimentary rocks and coincide with mild folding of the seafloor ( Figure 4). The listric faults are associated with both reverse and normal offsets of the Top-basement reflection (black lines in Figure 4), therefore suggesting that they correspond to early Cenozoic Eurekan thrusts reactivated as normal faults during the opening of the Fram Strait. The merging geometry of these faults with mylonitic surfaces within the Kinnhøgda–Daudbjørnpynten fault zone suggest that the latter controlled the formation of the former ( Figure 4). By contrast, the subvertical geometry of the brittle faults just north of the Kinnhøgda–Daudbjørnpynten fault zone and the associated triangular uplift and minor or lack of vertical offset of seismic reflections across these faults suggest that they accommodated dominantly strike-slip movement (orange lines in Figure 4).

 The gently folded geometry of the Risen and Kinnhøgda–Daudbjørnpynten fault zones and internal symmetric (to mildly asymmetric) reflections in E–W-oriented (along-strike) seismic sections and their undulating geometry in map view suggest reworking of the shear zones into open folds during a subsequent tectonic event involving contraction (sub-) parallel or slightly oblique to the shear zones ( Figure 3 and Figure 6). This is further discussed in the first chapter of the discussion.

 The relationship of the conical ridge with pinching out reflection packages within mid-upper Cenozoic sedimentary rocks overlying basement rocks suggest that the ridge consists of material younger than the age of the local Precambrian–early Paleozoic basement rocks. The Christmas-tree geometry of the ridge and associated pinching-out packages suggest that it may represent a salt diapir with mass transport deposits and carbonate mounds on a diapir’s flanks (e.g., Giles & Rowan, 2012), or a volcanic cone with draping lava sequences ( Magee *et al*., 2019; Phillips & Magee, 2020). Based on the geology of nearby onshore areas, the presence of evaporites in metamorphosed pre-Caledonian basement rocks is considered highly unlikely. However, Miocene lava flows are found at various localities in northern Spitsbergen ( Prestvik, 1978; Skjelkvåle *et al*., 1989). It is therefore probable that the conical ridge and pinching-out packages reflect Miocene magmatism.

## Discussion

### Timing of formation of the margin-oblique shear zones and related structures

The E–W and WNW–ESE strikes and top-north and top-SSW kinematics of internal structures (e.g., north- and SSW-verging folds; Figure 2, Figure 4, and Figure 5a–b) of the two interpreted shear zones northwest and west of Spitsbergen suggest that they unlikely formed during the Caledonian or Eurekan events, which resulted in margin-parallel, N–S- to NNW–SSW-striking, dominantly east-verging folds and top-east thrusts (e.g., Bergh & Grogan, 2003; Birkenmajer, 1975; Birkenmajer, 2004; Dallmann *et al*., 1993; Dumais & Brönner, 2020; Gee *et al*., 1994; Hjelle, 1979; Johansson *et al*., 2004; Johansson *et al*., 2005; Lyberis & Manby, 1999; Lyberis & Manby, 2001; Maher, 1988; Maher *et al*., 1986; Maher *et al*., 1989; Maher *et al*., 1997; Manby, 1986; Manby & Lyberis, 2001a; Manby & Lyberis, 2001b; Tessensohn *et al*., 2001a; Tessensohn *et al*., 2001b; Tessensohn *et al*., 2001c; Welbon & Maher, 1992; Witt-Nilsson *et al*., 1998; von Gosen & Piepjohn, 2001). The shear zones strike (sub-) parallel to Timanian structures in northern Norway, northwestern Russia, the Barents Sea, and Svalbard ( Faehnrich *et al*., 2020; Gabrielsen *et al*., 2022; Herrevold *et al*., 2009; Klitzke *et al*., 2019; Koehl *et al*., 2022a; Koehl *et al*., 2023a; Koehl *et al*., 2023b; Korago *et al*., 2004; Kostyuchenko *et al*., 2006; Lopatin *et al*., 2001; Lorenz *et al*., 2004; Majka *et al*., 2008; Majka *et al*., 2012; Mazur *et al*., 2009; Olovyanishnikov *et al*., 2000; Siedlecka, 1975). In addition, they show a similar geometry (i.e., moderately dipping in seismic cross section and undulating geometry in map view and in along-strike seismic sections; Figure 3 and Figure 6), consist of similar structures (e.g., mylonitic fault surfaces, duplexes, asymmetric folds and thrusts), and are located at a similar depth (i.e., c. 0.5–9.0 s TWT) as most Timanian thrusts in the Barents Sea and Svalbard. It is therefore probable that both shear zones formed during the Timanian Orogeny in the late Neoproterozoic. This is notably supported by the alignment of the shear zone west of Nordenskiöld Land with the northern edge of the Kinnhøgda–Daudbjørnpynten fault zone in Storfjorden in the Barents Sea ( Figure 1 and Figure 6).

The undulating geometry of the shear zones both in map view and in along-strike seismic sections suggests folding during a post-Timanian event involving E–W-oriented contraction. This is consistent with the largely accepted occurrence of the Caledonian Orogeny in Svalbard, which partly reworked Timanian thrusts and shear zones into N–S-striking folds in northern Norway, the Barents Sea, and Svalbard ( Gabrielsen *et al*., 2022; Koehl *et al*., 2022a; Koehl *et al*., 2023a; Siedlecka & Siedlecki, 1971). Notice the coincidence along a N–S- to NNW–SSE-trending axis of the wide, south- and north-plunging anticlines respectively of the Risen and Kinnhøgda–Daudbjørnpynten fault zones and of Prins Karls Forland, which may very well be part of the same Caledonian anticline ( Figure 6). Further reworking and overprinting occurred during the Eurekan event in the early Cenozoic and in the late Cenozoic during rifting. Eurekan contractional deformation is suggested by the minor reverse offsets (a few hundreds of meters) of the Top-basement reflection by a north-dipping brittle thrust northwest of Spitsbergen ( Figure 2) and by listric reverse faults west of Nordenskiöld Land ( Figure 4). Late Cenozoic rift-related overprinting is supported by normal offsets by listric faults and strike-slip faulting west of Nordenskiöld Land ( Figure 4). The coincidence of the strike-slip faults with gentle folding of the seafloor and the propagation of one of them up to the seafloor reflection suggest very recent strike-slip movement. The location of these faults and their strike coincide and align with that of the Molloy Fracture Zone ( Figure 1). Since the minor strike-slip faults seem to die out to the west (Supplement S3), they are not directly linked with the Molloy Fracture Zone. Nevertheless, the 60 km wide, hundreds of kilometers long Kinnhøgda–Daudbjørnpynten fault zone represents a major discontinuity in the crust and it is therefore probable that it controlled the formation and NNE-dipping geometry (e.g., Koehl *et al*., 2021; Thiede *et al*., 1990) of the Molloy Fracture Zone in the late Cenozoic.

The study area north of Spitsbergen was previously suggested to consist of a U-shaped Devonian collapse basin based on seismic refraction data ( Ritzmann & Jokat, 2003). The northern flank of the basin (see their Figure 8) coincides with the location c. 50 km north of Spitsbergen and mimics the south-dipping geometry of the Risen fault zone (depth of ca. 2.5–3.0 seconds TWT; Figure 2). The E–W trend of the basin does not fit that of Devonian basins in Svalbard, e.g., N–S-striking Andrée Land and Raudfjorden basins in northern Spitsbergen ( Braathen *et al*., 2018; Braathen *et al*., 2020; Burov & Semevskij, 1979; Dallmann & Piepjohn, 2020; Friend & Moody-Stuart, 1972; Friend *et al*., 1966; Friend *et al*., 1997; Gee & Moody-Stuart, 1966; Manby & Lyberis, 1992; McCann, 2000; Murascov & Mokin, 1979). In addition, the overall U-shaped (folded?) geometry of the basin is not compatible with that of a Devonian basin in Svalbard because of the lack of a major N–S-oriented contractional event after the Timanian Orogeny. Notably, recent studies suggested that the Late Devonian Svalbardian Orogeny did not occur in Svalbard ( Berry & Marshall, 2015; Koehl *et al*., 2022b; Lindemann *et al*., 2013; Marshall *et al*., 2015; Newmann *et al*., 2019; Newmann *et al*., 2020; Newmann *et al*., 2021; Scheibner *et al*., 2012). It is therefore more probable that the basin north of Spitsbergen consists of pre-Caledonian metasedimentary rocks, which were folded during the Timanian and Caledonian orogenies. Nevertheless, post-Caledonian collapse may have occurred along the inherited Timanian Risen fault zone as indicated by the extensional duplexes within the shear zone ( Figure 2 and Figure 5c–d).

West of Nordenskiöld Land, part of the interpreted continuation of the Kinnhøgda–Daudbjørnpynten fault zone was previously interpreted as an extensional detachment crosscut by the Hornsund Fault Complex to the west ( Blinova *et al*., 2009). This is in agreement with the interpreted extensional reactivation of the shear zone (e.g., Z-shaped extensional duplexes; Figure 4). However, although previous studies did partly notice the uplift of the Top-basement reflection along the shear zone (see WNW–ESE-striking ridge within the Bellsund Graben in Blinova *et al*., 2009 their Figure 11), they did not recognize evidence of top-SSW contractional deformation within the shear zone ( Figure 4). In addition, although the shear zone is partly eroded to the west in the hinge of the major north-plunging anticline ( Figure 6), it continues westwards below Cenozoic sedimentary rocks (Supplement S3), across the location of the Hornsund Fault Complex and of the De Geer Zone ( Figure 1 and Figure 6). Blinova *et al*. (2009) also identified minor strike-slip faults in the area, although they ascribed them E–W rather than WNW–ESE strikes.

### Implications for the De Geer Zone and plate tectonics reconstructions

The De Geer Zone and its main segment, the Hornsund Fault Complex, are believed to run ≤ 50 km (presumably less) west of Spitsbergen and to continue farther north along the western edge of the Yermak Plateau (e.g., Faleide *et al*., 2008; Geissler & Jokat, 2004) or to step or bend to the east onto the Yermak Plateau ( Kristoffersen *et al*., 2020). The occurrence of two undisrupted, late Neoproterozoic, WNW–ESE- to E–W-striking shear zones (Risen and Kinnhøgda–Daudbjørnpynten fault zones) extending at least 80 km west of the coastline of northwest and west of Spitsbergen and not showing any sign of lateral or vertical offset ( Figure 1, Figure 2, Figure 4, and Figure 6, and Supplements S2 and S3) unambiguously indicates that hundreds of kilometers dextral movements along the De Geer Zone and related faults like the Hornsund Fault Complex did not occur. This suggests that the De Geer Zone, which was largely speculated from the N–S-trending and linear morphology of the western Barents Sea–Svalbard and conjugate northern Greenland margins ( De Geer, 1926; du Toit, 1937; Harland, 1961; Harland, 1967; Harland, 1969; Horsfield & Maton, 1970; Wegmann, 1948) does not exist, and that its main fault segment, the Hornsund Fault Complex, most likely accommodated vertical fault movements as suggested by its listric geometry ( Austegard *et al*., 1988; Eiken, 1994; Geissler & Jokat, 2004).

Notably, Austegard *et al*. (1988) reported that all the structures west of Svalbard are extensional and that there are only very few occurrences of strike-slip movements. In addition, the only sparse evidence potentially indicating lateral movement is conflicting. For example, the possible sinistral strike-slip sense of shear indicated by right stepping geometries of margin-parallel brittle faults ( Eiken & Austegard, 1987) contrast with the major component of dextral strike-slip tectonics required for the commonly proposed sheared margin model of the De Geer Zone ( du Toit, 1937; Faleide *et al*., 1993; Harland, 1961; Harland, 1967; Harland, 1969; Horsfield & Maton, 1970; Lepvrier, 1990; Lepvrier & Geyssand, 1985; Steel & Worsley, 1984; Steel *et al*., 1981; Steel *et al*., 1985; Wegmann, 1948). This is consistent with our interpretation of a general lack of lateral movement along N–S-striking structures and with that of most previous offshore studies along the western Barents Sea–Svalbard margin (e.g., Eiken, 1994; Riis & Vollset, 1988).

Another argument against the existence of the De Geer Zone or any N–S-striking Cenozoic paleo-transform fault along the western Barents Sea–Svalbard margin is the inferred displacement rate, which is way too high. The De Geer Zone is believed to have accommodated about 400 km from breakup in the northeastern Atlantic and the Arctic oceans at ca. 56 Ma to breakup in the Fram Strait at ca. 24 Ma ( du Toit, 1937; Faleide *et al*., 1993; Harland, 1961; Harland, 1967; Harland, 1969; Horsfield & Maton, 1970; Wegmann, 1948). This amounts to a rate of 125 mm per year, i.e., approximately three times more than the San Andreas fault in California. By comparison, the San Andreas fault is typically believed to have accommodated movements in the range of 30 to 50 mm per year for the past 10 million years, i.e., c. 40 km of total lateral displacement (e.g., Crowell, 1979; Grant Ludwig *et al*., 2019; Huffman, 1972; Molnar & Atwater, 1973). A major issue is that the San Andreas fault accommodates transform movements along a margin adjacent to a fast-spreading ridge (with matching half spreading rate and fault displacement), whereas the mid-ocean ridge in the northeastern Atlantic and Arctic oceans is a slow- to ultra-slow-spreading ridge and therefore cannot justify such a large lateral slip rate along any paleo-transform like the De Geer Zone (e.g., Müller *et al*., 2008). Note that the current rate of movement along the San Andreas fault is 20 to 35 mm per year based on the data of the Southern California Earthquake Data Center ( scedc.caltech.edu/earthquake/sanandreas.html), i.e., lower than the estimate used here for the past 10 million years. This is supported by many other studies (e.g., Murray *et al*., 2014; Titus *et al*., 2005; van der Woerd *et al*., 2006). This inconsistency further illustrates the incompatibility of a major, thousands of kilometers long, N–S-striking transform fault off the western coasts of Svalbard and northern Greenland with the geological history of the area and all the current datasets.

It must be said that hundreds of kilometers of lateral movements along the De Geer Zone are not required to explain the geometry of the Svalbard and Greenland margins and the opening of the Fram Strait. Firstly, half of the distance Svalbard moved away from Greenland in the Cenozoic (c. 200 km) was accommodated by lateral movements along the two, c. 200 km long, NW–SE-striking transform faults in the Fram Strait, the Molloy and Spitsbergen fracture zones ( Crane *et al*., 1982; Johnson & Eckhoff, 1966; Myhre & Thiede, 1995; Thiede *et al*., 1990). Secondly, other mechanisms may very well account for the remaining 200 km movements. Among others, the reactivation of dominantly top-SSW Timanian thrusts during the Eurekan event (e.g., Koehl, 2020; Koehl, 2021; Koehl *et al*., 2022a; present study, Figure 2 and Figure 4) and associated folding along a WNW–ESE-trending axis in the early Cenozoic, e.g., in the Sørvestnaget Basin ( Kristensen *et al*., 2017), north of the Loppa High ( Koehl *et al*., 2023a), and north of Svalbard ( Figure 2), support such a claim. Preliminary results indicate that at least 150 km of post-Caledonian N–S shortening was accommodated by Timanian thrusts ( Koehl, 2020). However, more work is needed to refine this early estimate as more Timanian thrusts and related margin-oblique structures are being discovered (e.g., Risen fault zone). Nonetheless, the present results suggest major revisions in all Phanerozoic plate reconstructions for Arctic regions (e.g., Faleide *et al*., 2008; Nemcok *et al*., 2016) also because it suggests that the continent–ocean boundary in the Fram Strait is located at least 80–90 km to the west of Spitsbergen.

The present study also shows the danger of using mostly local onshore structural fieldwork in deeply eroded Arctic areas like Svalbard to resolve regional tectonic issues. Such biases are illustrated in Koehl and Allaart (2021), whose work shows that the Billefjorden Fault Zone, although representing a major tectonic discontinuity at a local scale (tens of kilometers long with hundreds of meter-scale displacement), does not represent a major regional tectonic boundary as previous thought (e.g., Harland, 1969; Harland *et al*., 1992). Another example is the Wegener Fault, a thousand of kilometer-long sinistral strike-slip fault inferred between Ellesmere Island and northwestern Greenland in the Nares Strait, which was proposed solely based on the physiographic morphology of the area, i.e., the linear geometry of the Nares Strait and tentative lateral offset of rock units on either side of the strait ( Taylor, 1910). Convincing evidence from geophysical datasets (e.g., gravimetric and aeromagnetic anomaly maps) and field mapping show that the bedrock continues across the Nares Strait with no apparent lateral offset and that the Wegener Fault does not exist ( Oakey & Chalmers, 2012; Oakey & Damaske, 2006; Oakey & Stephenson, 2008; Rasmussen & Dawes, 2011; see also further references and arguments in Gion *et al*., 2017). Despite overwhelming evidence against the Wegener Fault, field geologists continue to take its existence as a fact and use it to discuss incomplete and/or sparse field observations and interpretations (e.g., Gilotti *et al*., 2018; von Gosen *et al*., 2019). This calls for strengthened collaborations between geophysicists and field geologists and further highlights the importance of interdisciplinary studies. It is also necessary to clearly segregate faults observed directly on specific datasets (e.g., during fieldwork and/or on geophysical datasets) from tentative faults (i.e., inferred and not directly observed on any specific dataset) for example by calling the latter “lineaments” or “zones” and by clearly reporting the amount and nature of the uncertainty associated with the interpretation of the involved datasets. This especially includes data collected and observations made during fieldwork, whose interpretation is no less subjective than that of geophysical datasets.

## Conclusions

Two several kilometers wide south- and NNE-dipping shear zones of probable late Neoproterozoic age, the Risen and Kinnhøgda–Daudbjørnpynten fault zones, extend past the presumed location of the De Geer Zone west of Spitsbergen. The shear zones geometry and kinematics are consistent with a formation during the Timanian Orogeny. Both fault zones are continuous and do not show any trace of lateral offset, thus suggesting that the De Geer Zone does not exist and that faults initially associated with the De Geer Zone accommodated dominantly vertical movements. The present results therefore suggest major revisions to all current Phanerozoic paleogeographic reconstructions for Arctic regions.

The present study shows the importance of interdisciplinary approaches when trying to resolve large-scale tectonics and calls for caution with the extrapolation of local fieldwork data from deeply eroded Arctic regions to larger areas without supporting regional (e.g., geophysical) evidence. An important task for future studies is to distinguish directly observed faults from indirectly inferred structures by using a discrete nomenclature for the latter (e.g., “lineament” or else) and further encourage the discussion of the uncertainty associated to new and past interpretations.

## Ethics and consent

Ethical approval and consent were not required.

## Data Availability

DataverseNO: Underlying data for ‘The myth of the De Geer Zone’, https://doi.org/10.18710/J98MLA ( Koehl, 2023a) This project contains the following underlying data: ReadMe.txt. Figure 1– Figure 6 (high resolution versions of the figures included in this manuscript, in jpg format. All copyright permissions granted). Supplement Figures 1–3 (high-resolution versions of the supplementary figures included in the extended dataset, Koehl, 2023b, in jpg format. All copyright permissions granted). The Two-Way Time seismic reflection data analyzed in the present contribution is from the DISKOS database (Norwegian National Data Repository for Petroleum Data) of the Norwegian Petroleum Directorate and from the University of Bergen. Access to the data for research purposes can be obtained by contacting the Norwegian Petroleum Directorate at https://www.npd.no/om-oss/kontakt-oss/ and Prof. Rolf Mjelde from the University of Bergen ( Rolf.Mjelde@uib.no). DataverseNO: Extended data for ‘The myth of the De Geer Zone’, https://doi.org/10.18710/KUQNII ( Koehl, 2023b) This project contains the following extended data: ReadMe.txt. Koehl_ *et*_ *al*._supplements.docx (supplementary information and data to the present contribution including an overview of the seismic reflection database used in the presebt study and supplementary interpreted and uninterpreted seismic sections. All copyright permissions granted). Koehl_ *et*_ *al*._supplements.pdf (pdf version of the above-described document). Data are available under the terms of the Creative Commons Zero "No rights reserved" data waiver (CC0 1.0 Public domain dedication).
