# The myth of the De Geer Zone: a change of paradigm for the opening of the Fram Strait

**DOI:** 10.12688/openreseurope.16791.2

**Published:** 2025-03-17

**Authors:** Jean-Baptiste P. Koehl

**Affiliations:** 1 Department of Earth and Planetary Sciences, McGill University, Montreal, Québec, H3A 0E8, Canada; 2 Department of Geosciences, Universitetet i Oslo, Oslo, 0371, Norway

**Keywords:** Svalbard, Fram Strait, transform fault, thrust fault, shear zone, Cenozoic, De Geer Zone, Hornsund Fault Complex

## Abstract

**Background:**

Cenozoic rifting in the Arctic and the resulting opening of the Labrador Sea and the Fram Strait are typically associated with the movement of the Svalbard Archipelago c. 400 km southwards and its separation from Greenland. Thus far, most of this tectonic displacement was ascribed to lateral movement along the N–S-striking De Geer Zone, a thousand-kilometer-long paleo-transform fault believed to extend from northwestern Norway to northern Greenland.

**Methods:**

The study presents a new interpretation of tectonic structures on seismic reflection data north and west of Svalbard.

**Results:**

The present study reports the presence of two km-thick, hundreds of kilometers long, E–W- to WNW–ESE-striking shear zones, northwest and west of the island of Spitsbergen, Svalbard, in the Norwegian Arctic. Contractional structures within the shear zones, their strike, the inferred transport direction, and the great depth at which they are found indicate that they formed during the Timanian Orogeny in the late Neoproterozoic (c. 650–550 Ma). These structures extend at least 80–90 km west of the coastline of Spitsbergen. The presence of continuous, late Neoproterozoic Timanian thrusts this far west of Spitsbergen invalidates the occurrence of c. 400 km lateral movements along the N–S-striking De Geer Zone along the western Barents Sea–Svalbard margin in the Cenozoic.

**Conclusions:**

The present results suggest that the De Geer Zone does not exist and that related fault complexes (e.g., Hornsund Fault Complex) did not accommodate any strike-slip movement. In addition, the formation of major NW–SE-striking transform faults in the Fram Strait was controlled by late Neoproterozoic Timanian thrust systems. The present results call for major revisions of all current plate tectonics models for the opening of the Fram Strait and Arctic tectonics in the Cenozoic and for critical reviews of major fault zones inferred from indirect observations.

## Plain language summary

Thus far, the Svalbard Archipelago and Greenland are thought to have started drifting away from one another during the opening of the Labrador Sea and subsequently of the Fram Strait in the last 60 million years. This resulted in the displacement of Svalbard c. 400 km to the south at its present location. Thus far, most of this lateral displacement was commonly believed to have occurred along the De Geer Zone, a major, N–S-striking, presently inactive fault that was proposed to run in a linear fashion along the coastline of western Svalbard and extending from northwestern Norway to northern Greenland. The present results indicate the presence of two, 650–550 million years old, several kilometers thick, hundreds of kilometers long, WNW–ESE-striking fault zones, which extend well past the speculated location of the <60 million years old De Geer Zone. The present study therefore suggest that the De Geer Zone does not exist and that the area’s tectonic history was largely controlled by WNW–ESE-striking fault zones, e.g., controlling the location of major active lateral fault zones in the Fram Strait (e.g., Molloy Fracture Zone). The study also suggests a need for a revision of the nomenclature related to fault zones.

## Introduction

### The paradigm

**Figure 1.**
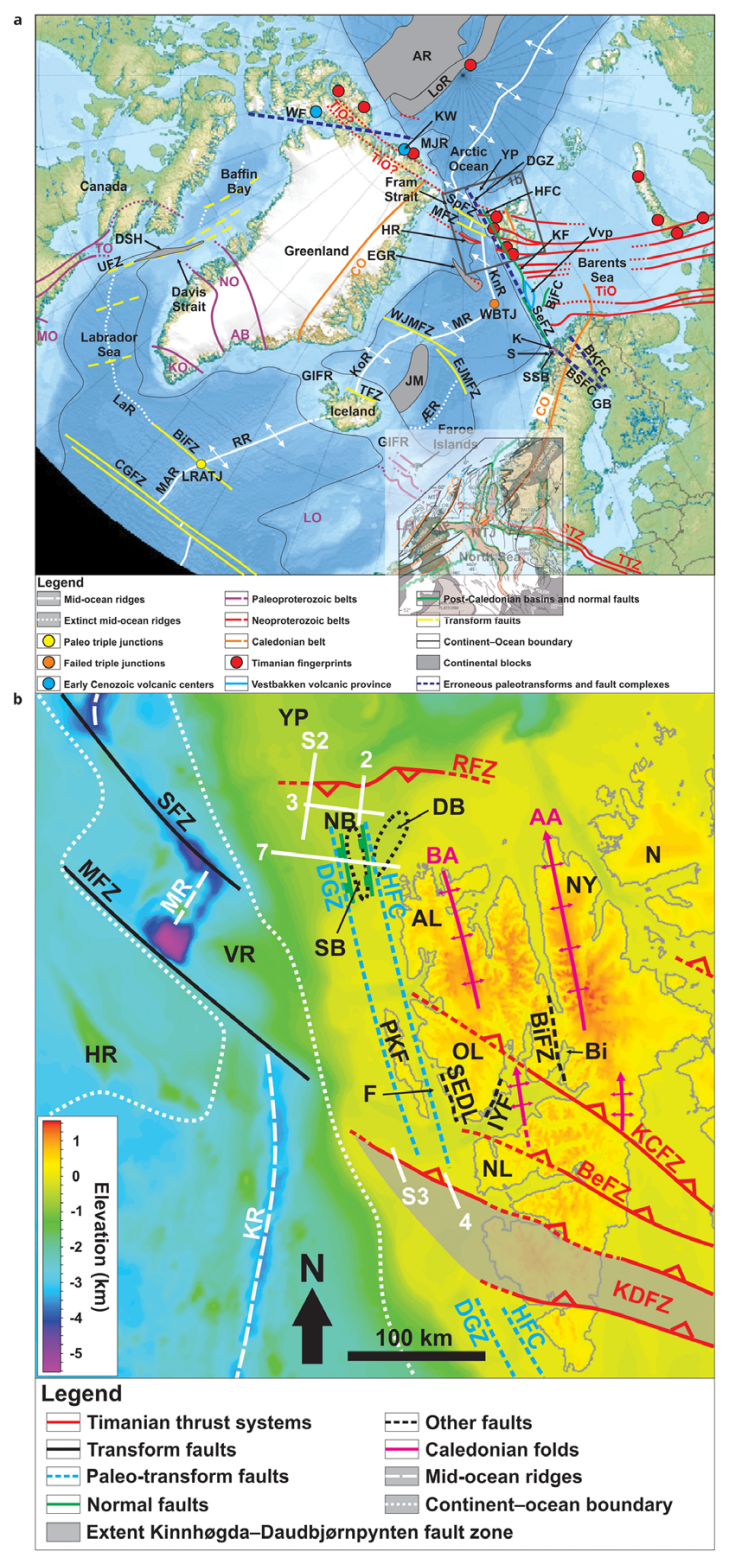
( **a**) International Bathymetric Map of the Arctic Ocean showing the continent–ocean boundary, continental blocks, major orogens and associated thrust faults, and major transform faults and rift basins and normal faults in Arctic regions. The De Geer Zone and its main three fault segments, the Hornsund Fault Complex, Knølegga Fault, and Senja Fracture Zone, are shown. Notice the apparent alignment along a NNW–SSE-trending axis of the De Geer Zone and its fault segments with onshore Precambrian structures (Senja Shear Belt and Bothnian–Senja Fault Complex). Modified after Koehl & Foulger (2024). Basemap is from Jakobsson *et al.* (2012). Abbreviations: AB: Ammassalik Belt; AR: Alpha–Mendeleev Ridge; BiFZ: Bight fault zone; BjFC: Bjørnøyrenna Fault Complex; BKFC: Bothnian–Kvænangen Fault Complex; BSFC: Bothnian–Senja Fault Complex; CGFZ: Charlie Gibbs fault zone; CO: Caledonian Orogen; DGZ: De Geer Zone; DSH: Davis Strait High; EGR: East Greenland Ridge; EJMFZ: East Jan Mayen fault zone; FB: Farsund Basin; GB: Gulf of Bothnia; HFC: Hornsund Fault Complex; HR: Hovgård Ridge; JM: Jan Mayen Microcontinent Complex; K: Kvaløya (island); KF: Knølegga Fault; KO: Ketilidian Orogen; KoR: Kolbeinsey Ridge; KnR: Knipovich Ridge; KW: Kapp Washington; LO: Laxfordian Orogen; LaR: Labrador paleo mid-ocean ridge; LoR: Lomonosov Ridge; LRATJ: Labrador–Reykjanes–Mid-Atlantic paleo triple junction; MAR: Mid-Atlantic Ridge; MF: Moray Firth; MFZ: Molloy Fault Zone; MJR: Morris Jesup Rise; MO: Makkovikian Orogen; MR: Mohns Ridge; NJT: North Sea failed triple junction; NO: Nagssugtoqidian Orogen; S: Senja (island); SeFZ; Senja Fracture Zone; SpFZ: Spitsbergen Fault Zone; SSB: Senja Shear Belt; STZ: Sorgenfrei–Tornquist Zone; TiO: Timanian Orogen; TO: Torngat Orogen; TTZ: Teisseyre–Tornquist Zone; UFZ: Ungava Fault Zone; Vvp: Vestbakken volcanic province; WBTJ: Western Barents Sea failed triple junction; WF: Wegener Fault; WJMFZ: West Jan Mayen fault zone; YP: Yermak Plateau; ÆR: Ægir Ridge. ( **b**) Location of the study area north and northwest of Spitsbergen (location shown as a brown frame in ( **a**)). The white lines show the location of the seismic sections discussed. The topographic–bathymetry map is from Jakobsson *et al*. (2012). The geology of the area is from Gee and Hjelle (1966), Johnson and Eckhoff (1966), Faleide *et al*. (1993), Myhre & Thiede (1995), Bergh *et al*. (1997), Maher *et al.* (1997), Witt-Nilsson *et al*. (1998), Bergh and Grogan (2003), Blinova *et al*. (2013), Braathen *et al*. (2018), Koehl and Allaart (2021), Kristoffersen *et al*. (2020), Koehl *et al*. (2022a). Abbreviations: AA: Atomfjella Antiform; AL: Albert I Land; BA: Bockfjorden Anticline; BeFZ: Bellsundbanken fault zone; Bi: Billefjorden; BiFZ: Billefjorden Fault Zone; DB: Danskøya Basin; DGZ: De Geer Zone; F: Forlandsundet; HFC: Hornsund Fault Complex; HR: Hovgård Ridge; IYF: Isfjorden–Ymerbukta Fault; KCFZ: Kongsfjorden–Cowanodden fault zone; KDFZ: Kinnhøgda–Daudbjørnpynten fault zone; KR: Knipovich Ridge; MFZ: Molloy fault zone; MR: Molloy Ridge; N: Nordaustlandet; NB: Nansen Bank; NF: Ny-Friesland; NL: Nordenskiöld Land; OL: Oscar II Land; PKF: Prins Karls Forland; RFZ: Risen fault zone; SEDL: Svartfjella–Eidembukta–Daudmannsodden Lineament; SFZ: Spitsbergen fault zone; VR: Vestnesa Ridge; YP: Yermak Plateau.

The De Geer Zone is a major structural element of the west Spitsbergen transform margin that is believed to have accommodated 400 kilometers of dextral strike-slip movement during the opening of the Northeast Atlantic and Arctic oceans and of the Fram Strait in the Cenozoic ( De Geer, 1926; du Toit, 1937; Faleide *et al*., 1993; Faleide *et al*., 2008; Harland, 1961; Harland, 1967; Harland, 1969; Holtedahl, 1936; Horsfield & Maton, 1970; Piepjohn *et al*., 2016; Wegmann, 1948; Figure 1a). This fault system is believed to have facilitated the movement of Svalbard towards the south in its present position.

It was first proposed based on the linear morphology of the northern coastline of Greenland and western Svalbard (e.g., De Geer, 1926; Holtedahl, 1936). Holtedahl (1936) notably described that the De Geer Zone is characterized by a series of submarine depressions west of Svalbard and the western Barents Sea extending into the fjords of northern Norway. Later on, a correlation was proposed between the southern offshore segment of the De Geer Zone, the Senja Fracture Zone, which runs along the edge of the southwestern Barents Sea margin (e.g., Myhre *et al.*, 1982), and the subvertical, late Paleoproterozoic, NW–SE-striking Senja Shear Belt onshore northwestern Norway ( Bergh *et al.*, 2010; Zwaan, 1995; Figure 1a). Major NE–SW-striking post-Caledonian fault complexes seem to change polarity (i.e., dip direction) across the Senja Shear Belt, which was thus interpreted to have been reactivated as a post-Caledonian transfer zone in the late Paleozoic–Cenozoic ( Indrevær *et al.*, 2013; Olesen *et al.*, 1993; Olesen *et al.*, 1997). Recent onshore studies have contributed evidence of strike-slip kinematics (e.g., slickensides) along post-Caledonian, NW–SE-striking brittle faults in the area, thus supporting the possible reactivation of the Senja Shear Belt as a strike-slip fault during the Cenozoic (e.g., Distelbrink, 2024). However, neither specific age constraints (e.g., geochronological analysis) nor precise estimates on the amount of strike-slip displacement accommodated by NW–SE-striking were obtained. Farther inland, the De Geer Zone and the Senja Shear Belt are believed to link up with the Bothnian–Senja Fault Complex, a presumed Proterozoic fault which extends to the Gulf of Bothnia ( Henkel, 1987; Henkel, 1991; Indrevær *et al.*, 2013).

Structural fieldwork onshore western Spitsbergen has shown that the area is dominated by N–S to NNE–SSW-striking faults, which show indications of up to 10 km dextral strike-slip displacement. Examples of such structures are the Isfjorden–Ymerbukta Fault ( Bergh *et al.*, 1997; Harland & Horsfield, 1974) and the Svartfjella–Eidembukta–Daudmannsodden Lineament ( Maher *et al.*, 1997; Figure 1b). However, the main paleo transform fault, which accommodated the missing hundreds km displacement between northern Greenland and western Svalbard was believed to run offshore west of Svalbard ( Maher *et al.*, 1997).

Other studies argued that Svalbard and northern Greenland must have been adjacent to one another prior to the Cenozoic based on similar rock units and tectono-magmatic episodes (e.g., Harland *et al.*, 1993; Jones *et al.*, 2016; Jones *et al.*, 2017; Majka *et al.*, 2021; Piepjohn *et al.*, 2016). Examples of tectonic episodes correlated on both margins include the presumed Late Devonian Ellesmerian–Svalbardian Orogen and the early Cenozoic Eurekan fold-and-thrust belt ( McClelland *et al.*, 2021; Piepjohn *et al.*, 2016; Tessensohn & Piepjohn, 2000). An example of magmatic episode is the link between Paleocene ash beds in central Svalbard with contemporaneous volcanism in northern Greenland and Arctic Canada ( Jones *et al.*, 2016; Jones *et al.*, 2017). Harland *et al.* (1993) also established regional correlations of Precambrian rock units in northern Greenland and Svalbard. These correlations were claimed as evidence of long-lived close proximity between the two regions.

A study of seismic reflection data along the western Barents Sea margin by Faleide *et al.* (1993) gave weight to the De Geer Zone hypothesis. The study notably suggested major dextral strike-slip movements along the Senja Fracture Zone and related faults such as the Bjørnøyrenna Fault Complex ( Figure 1a). Further plate tectonic modeling and paleogeographic reconstructions cemented the vision of the De Geer Zone by showing that the De Geer Zone hypothesis is plausible from a plate kinematic perspective (e.g., Faleide *et al.*, 2008; Shephard *et al.*, 2013).

### Cracks in the paradigm

The De Geer Zone strikes N–S and is thus oblique to the currently active NW–SE-striking Molloy and Spitsbergen fault zones ( Figure 1a), which implies a major change in plate kinematics at breakup (ca. 24 Ma; Engen *et al*., 2008). This change in spreading and plate movement direction is not explained in studies targeting the evolution of the Svalbard–Greenland transform margin (e.g., Doré *et al.*, 2015; Faleide *et al.*, 2008; Nemcok *et al.*, 2016), and its origin remains a mystery should such a change have occurred.

In addition, despite numerous studies both onshore and offshore along the western Svalbard margin, the actual trace of the De Geer Zone remains a matter of debate ( Bergh & Grogan, 2003; Geissler & Jokat, 2004; Myhre *et al*., 1982; Vogt *et al*., 1981). This is notably related to the striking lack of evidence of lateral movement on seismic reflection datasets (e.g., Austegard *et al*., 1988; Eiken, 1994; Gabrielsen *et al*., 1990; Lasabuda *et al*., 2018; Riis & Vollset, 1988), apart from a few pieces of evidence of minor sinistral strike-slip displacement ( Eiken & Austegard, 1987). This contrasts markedly with the dominant dextral component of movement along the De Geer Zone ( du Toit, 1937; Faleide *et al*., 1993; Harland, 1961; Harland, 1967; Harland, 1969; Horsfield & Maton, 1970; Lepvrier, 1990; Lepvrier & Geyssand, 1985; Steel & Worsley, 1984; Steel *et al*., 1981; Steel *et al*., 1985; Wegmann, 1948).

The study by Faleide *et al.* (1993) provided no robust evidence supporting the model of the De Geer Zone they proposed. In addition, their study did not include the seismic reflection data they used, only interpretation sketches. Furthermore, the fault zones they suggested to be major strike-slip and/or transform faults, e.g., Senja Fracture Zone and Bjørnøyrenna Fault Complex ( Figure 1a), show listric and moderately-dipping geometries (e.g., Gabrielsen *et al.*, 1997; Koehl *et al.*, 2023a), which are incompatible with large-scale strike-slip displacement.

Moreover, movement along NW–SE-striking faults in Senja and Kvaløya is yet to be accurately constrained as, as up to now, no geochronological constraints are available for these faults. Geochronological studies of NE–SW-striking brittle faults in the area (e.g., Davids *et al.*, 2013) and NW–SE-striking faults in northern Norway ( Koehl *et al.*, 2018a; Torgersen *et al.*, 2014) indicate that most brittle faults are late Paleozoic, which is not in line with previously proposed major strike-slip reactivation of onshore Precambrian shear zones and fault systems (e.g., Distelbrink, 2024; Olesen *et al.*, 1997).

Furthermore, there is no trace of major faulting or indicators of large (tens to hundreds of km) strike-slip displacement along NW–SE-striking faults in northwestern Norway (e.g., Indrevær *et al.*, 2013; Koehl *et al.*, 2019). In addition, the Bothnian–Senja and Bothnian–Kvænangen fault complexes were initially inferred from elongated NW–SE-striking magnetic anomalies, which are now known to reflect basement features such as late Paleoproterozoic Svecofennian–Svecokarelian folds and greenstone belts (e.g., Henderson *et al.*, 2015; Koehl *et al.*, 2019), thus further casting doubt on the occurrence of major strike-slip movements in northwestern Norway.

### Goals of the study

**Figure 2.**
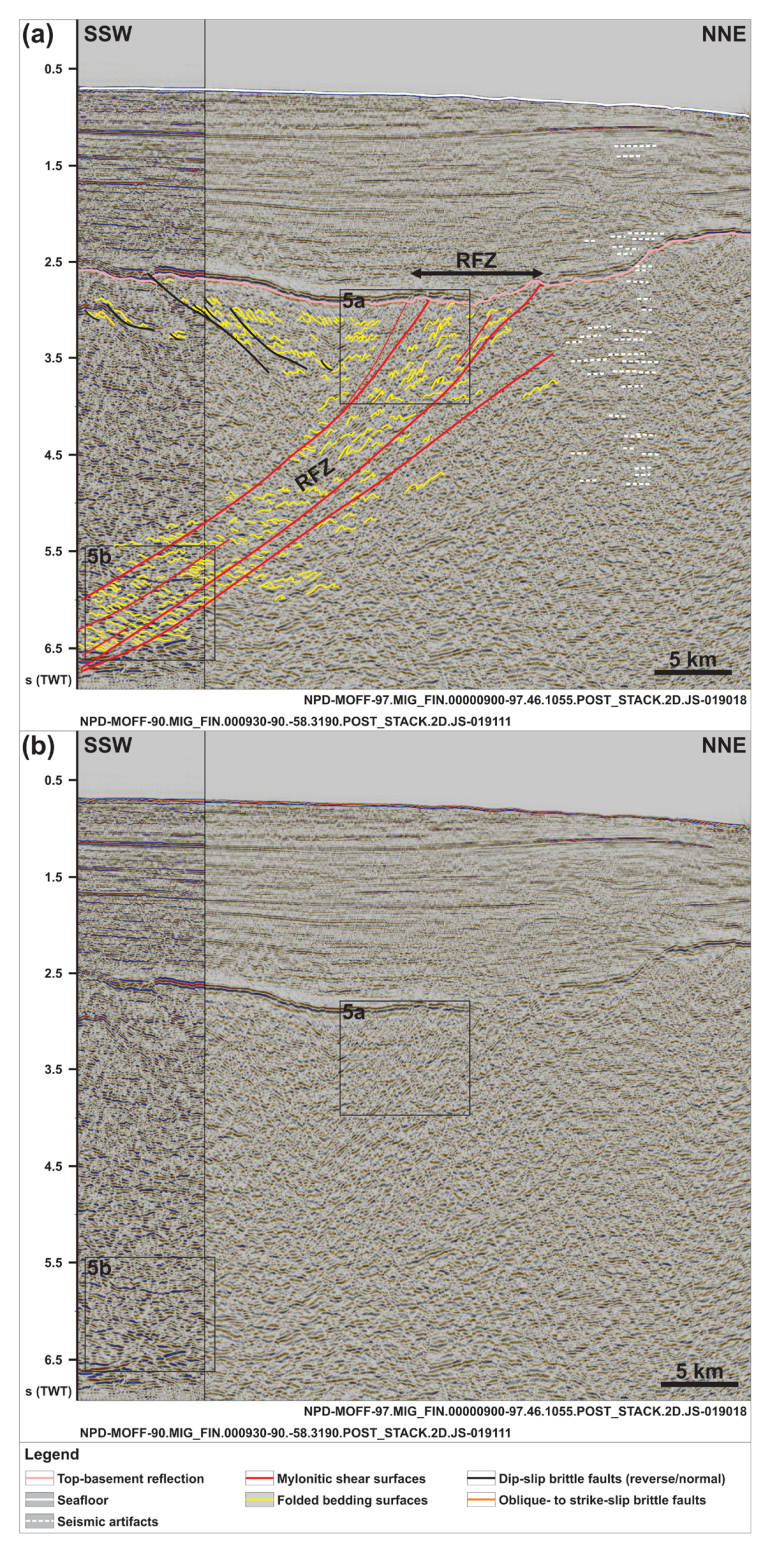
( **a**) Interpreted and ( **b**) uninterpreted N–S-oriented seismic section showing the south-dipping geometry of the Risen fault zone and that of internal north-verging folds, extensional duplexes, and mylonitic shear surfaces. Notice the reverse offset of the Top-basement reflection by a minor top-south thrust in the south and a number of seismic artifacts just north of the Risen fault zone (dashed white lines). The vertical black line in the data indicates a change of dataset (i.e., intersection of two seismic lines). Location is shown in Figure 1b. Abbreviation: RFZ: Risen fault zone.

**Figure 3.**
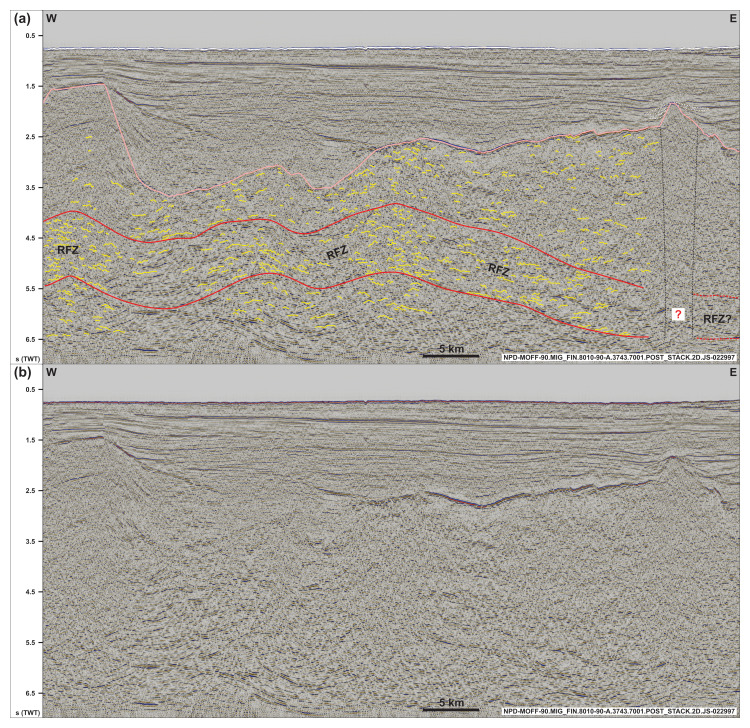
( **a**) Interpreted and ( **b**) uninterpreted along-strike seismic section showing the undulating, gently folded geometry of the Risen fault zone and related basement structures within and around the shear zone (dominantly symmetric open folds). Notice the washed-out zone below the conical ridge in the east (dotted black lines), which is possibly related to magmatic intrusions below a volcanic cone and associated lava flows (dotted white lines). See location in Figure 1b and legend in Figure 2. Abbreviation: RFZ: Risen fault zone.

**Figure 4.**
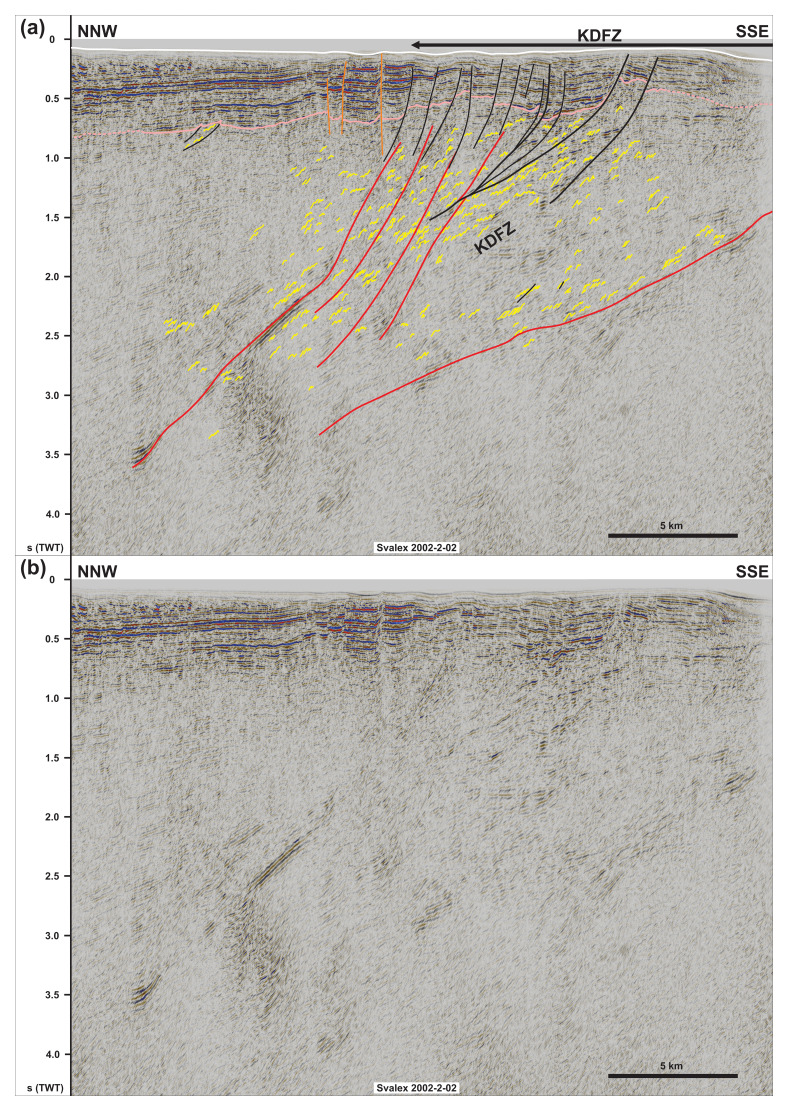
( **a**) Interpreted and ( **b**) uninterpreted NNW–SSE seismic section showing the northern flank of the NNE-dipping Kinnhøgda–Daudbjørnpynten fault zone and that of internal south-verging folds, duplexes, and mylonitic shear surfaces. Notice the steeply to moderately NNE-dipping geometry of the shallow brittle faults in the south indicating mostly dip-slip kinematics, whereas shallow brittle over the northernmost edge of the Kinnhøgda–Daudbjørnpynten fault zone in the north are subvertical thus indicating a strike-slip component. Abbreviation: KDFZ: Kinnhøgda–Daudbjørnpynten fault zone.

The present study targets the Hornsund Fault Complex northwest of Spitsbergen and two E–W- to WNW–ESE-striking structures northwest and west of Spitsbergen (the Risen and Kinnhøgda–Daudbjørnpynten fault zones), the latter of which extend c. 80–90 km west of the coastline of Spitsbergen, i.e., west of the presumed location of the De Geer Zone ( Figure 1b). The cross-section geometry of the former is interpreted in E–W-oriented 2D seismic sections, while the latter two structures appear on several, NNW–SSE- to NNE–SSW- as well as E–W-oriented 2D seismic lines analyzed in the present study ( Figure 2, Figure 3, and Figure 4). The present contribution describes the overall geometry of these structures and that of minor internal structures. Internal structures are further discussed to resolve the kinematics and reactivation history of the main faults and shear zones. The geometry and kinematics were then used to infer the possible timing of formation of the structures. The results have major implications for the opening of the Fram Strait, fault kinematics along sheared/transform margins, e.g., western Barents Sea–Svalbard margin and other transform margins worldwide, and for the interpretation of major paleo-transform faults, e.g., De Geer Zone and Wegener Fault.

### Geological setting

***Timanian Orogeny.*** The Timanian Orogeny is a major episode of overall top-SSW, late Neoproterozoic (650–550 Ma) contraction, during which continental lithosphere formed in the Arctic (e.g., Estrada *et al*., 2018a; Estrada *et al*., 2018b; Gee *et al*., 2000; Pease *et al*., 2004; Rekant *et al*., 2019; Rosa *et al*., 2016). This tectonic episode was initially thought to be restricted to northeastern Norway ( Dallmeyer & Reuter, 1989; Gorokhov *et al*., 2001; Siedlecka, 1975; Siedlecka & Siedlecki, 1971) and northwestern Russia ( Kostyuchenko *et al*., 2006; Kuznetsov *et al*., 2007; Larionov *et al*., 2004; Lorenz *et al*., 2004; Olovyanishnikov *et al*., 2000; Pease *et al*., 2004; Remizov, 2006; Remizov & Pease, 2004). However, recent studies in Greenland ( Estrada *et al*., 2018a; Rosa *et al*., 2016), Arctic Canada ( Estrada *et al*., 2018b), the Lomonosov Ridge ( Rekant *et al*., 2019), Svalbard ( Dallmeyer *et al*., 1990a; Faehnrich *et al*., 2020; Koglin *et al*., 2022; Majka *et al*., 2008; Majka *et al*., 2012; Manecki *et al*., 1998; Peucat *et al*., 1989), and the Barents Sea ( Klitzke *et al*., 2019; Koehl, 2020; Koehl *et al*., 2022a; Koehl *et al*., 2023a) show that the Timanian Orogeny extends over a much broader area ( Figure 1a). These findings also indicate that the Svalbard Archipelago and the Barents Sea were already accreted to northern Norway in the late Neoproterozoic ( Koehl, 2020; Koehl *et al*., 2022a; Koehl *et al*., 2023a).

Most Timanian structures strike WNW–ESE to E–W and consist of asymmetric folds and mylonitic brittle–ductile thrusts and shear zones. These structures were later reworked into dome- and trough-shaped folds during Caledonian contraction, and reactivated and/or overprinted during Devonian–Carboniferous extension, early Cenozoic Eurekan contraction, and late Cenozoic rifting ( Faehnrich *et al*., 2020; Gabrielsen *et al*., 2022; Koehl *et al*., 2022a; Koehl *et al*., 2023a; Koehl *et al.*, 2025b awaiting peer review; Siedlecka & Siedlecki, 1971).

A structure of particular interest is the Kinnhøgda–Daudbjørnpynten fault zone, a 60 km wide, hundreds of kilometers long thrust system, which extends from the northern Barents Sea to Wedel Jarlsberg Land in southwestern Spitsbergen ( Figure 1b). There, the Vimsodden–Kosibapasset Shear Zone ( Mazur *et al*., 2009) is believed to represent the onshore continuation of the southern edge of the thrust system ( Koehl *et al*., 2022a).

***Caledonian Orogeny.*** Caledonian contraction at ca. 465–425 Ma resulted in the formation of N–S-striking fabrics and structures, both in Svalbard and the Barents Sea ( Birkenmajer, 1975; Birkenmajer, 2004; Braathen *et al*., 1999a; Dallmeyer *et al.*, 1990b; Dumais & Brönner, 2020; Faehnrich *et al.*, 2020; Gernigon *et al*., 2014; Gudlaugsson *et al*., 1987; Gudlaugsson *et al*., 1998; Hjelle, 1979; Horsfield, 1972; Johansson *et al*., 2004; Johansson *et al*., 2005; Koehl *et al*., 2022a; Koehl *et al*., 2023a; Manby, 1986; Witt-Nilsson *et al*., 1998). Major structures include a well-developed foliation ( Gee *et al*., 1992; Witt-Nilsson *et al*., 1998), brittle–ductile thrusts ( Birkenmajer, 1975; Birkenmajer, 2004; Manby, 1986), tens of kilometers wide, gently north-plunging folds and antiformal (thrust) stacks ( Dumais & Brönner, 2020; Gee & Hjelle, 1966; Witt-Nilsson *et al*., 1998; Figure 1a–b), and blueschist and eclogite metamorphism ( Dallmeyer *et al*., 1990b; Horsfield, 1972; Ohta *et al*., 1995). In northwestern Spitsbergen (i.e., closest to the study area), basement rocks consist of Grenvillian metasedimentary and metaigneous rocks, which were later reworked and intruded by granitic plutons during the Caledonian Orogeny ( Gee & Hjelle, 1966; Hjelle, 1979; Myhre *et al*., 2008; Pettersson *et al*., 2009a; Pettersson *et al*., 2009b).

***Devonian–Carboniferous extension.*** Late- to post-orogenic extensional collapse of the Caledonides resulted in the deposition of thick (c. 9–10 km thick) Devonian sedimentary rocks ( Dallmann & Piepjohn, 2020; Friend, 1961; Friend *et al*., 1997; Friend & Moody-Stuart, 1972; Gernigon *et al*., 2014; Murascov & Mokin, 1979) along low-angle detachments ( Braathen *et al*., 2018; Chorowicz, 1992; Koehl *et al*., 2018b; Maher *et al*., 2022; Roy, 2007; Roy, 2009) and inverted Timanian and Caledonian thrusts in northern Spitsbergen ( Koehl *et al.*, 2022a; Koehl *et al.*, 2023b). In the Carboniferous, extension slowed down, and kilometer-thick sedimentary rocks of the Billefjorden and Gipsdalen groups were deposited in subsiding basins, both along inherited Timanian and Caledonian fabrics ( Cutbill & Challinor, 1965; Cutbill *et al*., 1976; Koehl *et al*., 2018b; Koehl & Muñoz-Barrera, 2018; McCann & Dallmann, 1996; Samuelsberg *et al*., 2003; Smyrak-Sikora *et al*., 2018). Note that the Late Devonian Svalbardian Orogeny (potentially 383–365 Ma) is now thought not to have occurred in Svalbard and will therefore not be discussed in the present contribution ( Koehl *et al*., 2022b and references therein).

***Early Cenozoic Eurekan contraction.*** In the Paleocene, the opening of the Labrador Sea and possibly of Baffin Bay was accompanied by an episode of contraction in northern Greenland and western Spitsbergen, which resulted in the formation of the West Spitsbergen Fold-and-Thrust Belt ( Dallmann *et al*., 1993; Gion *et al*., 2017; Jones *et al*., 2017; Oakey & Chalmers, 2012). Major folds and thrusts in the belt strike N–S to NNW–SSE, i.e., parallel to the coastline in western Spitsbergen ( Bergh & Grogan, 2003; Dallmann *et al*., 1993; Lyberis & Manby, 2001; Maher, 1988; Maher *et al*., 1986; Maher *et al*., 1989; Maher *et al*., 1997; Manby, 1986; Manby & Lyberis, 2001a; Manby & Lyberis, 2001b; Tessensohn *et al*., 2001a; Tessensohn *et al*., 2001b; Tessensohn *et al*., 2001c; von Gosen & Piepjohn, 2001; Welbon & Maher, 1992).

Contraction faded as rifting and seafloor spreading initiated in the northeastern Atlantic and Arctic oceans at ca. 56 Ma near the Paleocene–Eocene boundary. Svalbard and Greenland are then believed to have gradually slid past one another along a major paleo-transform fault, the De Geer Zone, which is thought to have accommodated c. 400 km of dextral strike-slip movement prior to breakup in the Fram Strait ( du Toit, 1937; Faleide *et al*., 1993; Harland, 1961; Harland, 1967; Harland, 1969; Horsfield & Maton, 1970; Piepjohn *et al*., 2016; Wegmann, 1948). The main segment of the De Geer Zone, the Hornsund Fault Complex, was mapped on seismic data as a series of steep, east- and west-dipping faults bounding N–S-trending basement ridges, e.g., Nansen Bank in the west, from sedimentary basins, e.g., the Danskøya Basin in the east ( Figure 1b; Austegard *et al*., 1988; Bergh & Grogan, 2003; Eiken, 1994; Eiken & Austegard, 1987; Faleide *et al*., 1993; Gabrielsen *et al*., 1990; Geissler & Jokat, 2004; Grogan *et al*., 1999; Mann & Townsend, 1989; Myhre *et al*., 1982; Riis & Vollset, 1988; Vogt *et al*., 1981). However, indicators of strike-slip movements along the De Geer Zone and Hornsund Fault Complex are difficult to identify and are thought to have been overprinted by later extensional structures, which dominate the margin at present.

According to previous studies, the De Geer Zone and Hornsund Fault Complex are either one and the same structure (e.g., Bergh & Grogan, 2003; Faleide *et al*., 1993) or discrete structures (e.g., Kristoffersen *et al*., 2020; Piepjohn *et al*., 2016; Figure 1b). However, should they be separate structures, there is no direct evidence of the western one (the De Geer Zone). Nevertheless, the lack of evidence supporting strike-slip movement along the Hornsund Fault Complex ( Austegard *et al*., 1988; Eiken, 1994; Riis & Vollset, 1988) generates a need for an extra tentative (yet to be observed) fault zone to the west, farther offshore.

***Late Cenozoic rifting.*** Breakup in the Fram Strait may have initiated at ca. 24 Ma (Chron 7; Engen *et al*., 2008), i.e., much later than the northeastern Atlantic and the Arctic oceans (at ca. 56 Ma; Faleide *et al*., 1993; Faleide *et al*., 2008; Gaina *et al.*, 2002; Rowley & Lottes, 1988; Srivastana, 1985). From then, transform movements are thought to have been accommodated by two, c. 200 km long transform faults, the Molloy and Spitsbergen fault zones ( Crane *et al*., 1982; Johnson & Eckhoff, 1966; Myhre & Thiede, 1995; Thiede *et al*., 1990). At that time, N–S-striking faults such as the Hornsund Fault Complex were reactivated as normal faults, developed a listric geometry, and accommodated the deposition of thick mid–upper Cenozoic (Oligocene–?) Miocene–Quaternary sediments (e.g., Danskøya Basin; Eiken, 1993; Eiken, 1994; Geissler & Jokat, 2004; Geissler *et al*., 2011). The Forlandsundet Graben is generally thought to have formed during this stage although accurate age constraints for sediment deposition are still lacking ( Livshits, 1992; Manby, 1986; Schaaf *et al*., 2020). In addition, the relationship of the graben sediments with adjacent basement rocks (not necessarily faulted) indicates that a formation during the Eurekan episode might be possible too ( Gabrielsen *et al*., 1992; Kleinspehn & Teyssier, 1992; Kleinspehn & Teyssier, 2016; Lepvrier, 1990). Rifting was accompanied by magmatism in the Miocene as documented by lava flows onshore northern Svalbard ( Prestvik, 1978; Skjelkvåle *et al*., 1989).

## Methods

Two-Way Time (TWT) 2D seismic data from the Norwegian National Data Repository for Petroleum Data (DISKOS database) and of the University of Bergen around Spitsbergen were analyzed to map a basement-seated structure west of Spitsbergen (see Extended data: Supplement S1 for an overview of the database used ( Koehl, 2025)). Petrel (version 2021.3) was used to interpret the data, which may also be interpreted via OpendTect, a free open-source alternative software. The figures were designed using CorelDraw 2017 ( GIMP is a freely available open-source alternative). High-resolution versions of the figures and supplements are available on DataverseNO ( Koehl, 2025; https://doi.org/10.18710/J98MLA). These are necessary to observe the described structures in their full resolution. Additional seismic sections are also available online as electronic supplements on DataverseNO ( Koehl, 2025; https://doi.org/10.18710/J98MLA).

Notable structures interpreted include asymmetric folds within mylonitic shear zones and brittle–ductile thrust systems. The seismic facies of such asymmetric folds was first described by Koehl (2021) in central Svalbard. This study correlated uppermost Devonian–Mississippian coal measures of the Billefjorden Group sheared during early Cenozoic Eurekan contraction to their offshore equivalent. The onshore–offshore tie was facilitated by the relatively lower density of coal measures, which appear as bright negative high-amplitude reflections in seismic reflection data. In addition, they could be traces along the same fault zone (Balliolbreen Fault segment of the Billefjorden Fault Zone). Further studies of 2D–3D seismic reflection data and ties to exploration wells, gravimetric, magnetic, and bathymetric data as well as onshore outcrops have confirmed the character of asymmetric folds in seismic reflection data ( Koehl *et al.*, 2022a; Koehl *et al.*, 2023a; Koehl *et al.*, 2023b; Koehl *et al.*, 2024: Koehl *et al.*, 2025b; Koehl & Stokmo, 2024). Asymmetric folds in seismic reflection data typically appear as moderate-amplitude upward-curving reflections with asymmetric flanks, including a relatively long and gently-dipping flank and a narrower and steeper flank. The narrower and steeper flank indicates the direction of tectonic transport (sense of shear).

Other important and related structures include mylonitic shear surfaces. These have been known for some time and typically appear as bright, high-positive-amplitude, moderately- to gently-dipping planar reflections (e.g., Fazlikhani *et al.*, 2017; Fountain *et al.*, 1984; Hurich *et al.*, 1985; Phillips *et al.*, 2016), owing their high positive amplitude to the relatively high density of mylonite ( Bell & Etheridge, 1973; Sibson, 1977). The seismic character of mylonitic shear surfaces may vary, e.g., to less bright amplitude reflections or to simple disruption surfaces where the mylonite is not sufficiently developed (e.g., Koehl *et al.*, 2022a).

Major fault surfaces (including mylonites) generally disrupt seismic reflections and corresponding rock units. However, faults do not necessarily show as through-going disruption surfaces, and some seismic reflections may locally appear undisrupted or only mildly affected. This may occur in areas where the fault rock (e.g., mylonite) is not sufficiently developed and where the rock units on either side of the fault have comparable density and seismic velocity, which may thus not produce a sufficient acoustic impedance contrast (e.g., Kelly *et al.*, 2017). The key to interpreting fault zones is thus the alignment of numerous disruptions/discontinuities (i.e., truncated reflections) and nearby related structures, which may also indicate proximity to a major fault.

To interpret the data, our descriptions were compared to previous seismic studies around the Svalbard Archipelago and the Barents Sea and onshore field studies in Svalbard, as well as to other studies of seismic reflection data worldwide. Noteworthy, in order to be as conservative as possible, it was assumed that the De Geer Zone and the Hornsund Fault Complex are discrete structures, i.e., that the De Geer Zone might be located west of Prins Karls Forland although no tangible evidence supporting this has been found thus far ( Figure 1b). This implies that the hereby drawn conclusions would also be valid, if not more resounding, should these two structures be one.

When analyzing seismic data, numerous examples of seismic artifacts were identified. These are indicated on the interpreted seismic sections but are not further discussed as they are not the focus of the present work. Notable artifacts include multiples, diffraction, and bottom-simulating reflections.

The vertical resolution of the NPD-MOFF-90, NPD-MOFF-93, and NPD-MOFF-97 surveys is ¼ of the wavelength and is computed from the velocity of basement rocks in the study area (6300 m.s ^-1^; Gernigon *et al.*, 2018) and frequency of the data (low-cut filter at 5 Hz and frequencies up to 40 Hz; Kristiansen, 1999). An estimate of the vertical resolution at high depth (using 40 Hz) is therefore c. 39 m (and minimum 315 m using the 5 Hz low-cut filter frequency). The bin size of the NPD-MOFF-90, NPD-MOFF-93, and NPD-MOFF-97 surveys is 12.5–25 m x 25 m (cable group length and shot point interval; Kristiansen, 1999), thus yielding a horizontal resolution of c. 313–625 m at shallow level ( Table 1). The horizontal resolution of the surveys at a depth of 5000 m using a frequency of 40 Hz is c. 627 m ( Table 1).

**Table 1.** Comparison of the size of investigated structures with the horizontal (for depths of 0 and 5000 m and using a frequency of 40 Hz) and vertical resolution (for a frequency of 40 Hz and at a depth of 5000 m) of the seismic datasets used.

Survey/structures	Horizontal resolution (0 m/5000 m depth)/ width		Vertical resolution (5 Hz/40 Hz)/height
**Asymmetric folds**	> 500 m		> 150 m
**NPD-MOFF-90/93/97**	312.5–625 m	627.495 m	39.375 m
**Svalex**	625 m	627.495 m	39.375 m

Similarly, the vertical resolution of the Svalex survey is c. 39 m (40 Hz frequency; ( Dinctürk, 2022). The bin size for the Svalex data is 12.5 m x 50 m, thus yielding a horizontal resolution of c. 625 m at shallow level ( Dinctürk, 2022; Table 1). At a 5000 m depth, the horizontal resolution is c. 627 m using a 40 Hz frequency ( Dinctürk, 2022; Table 1).

**Figure 5.**
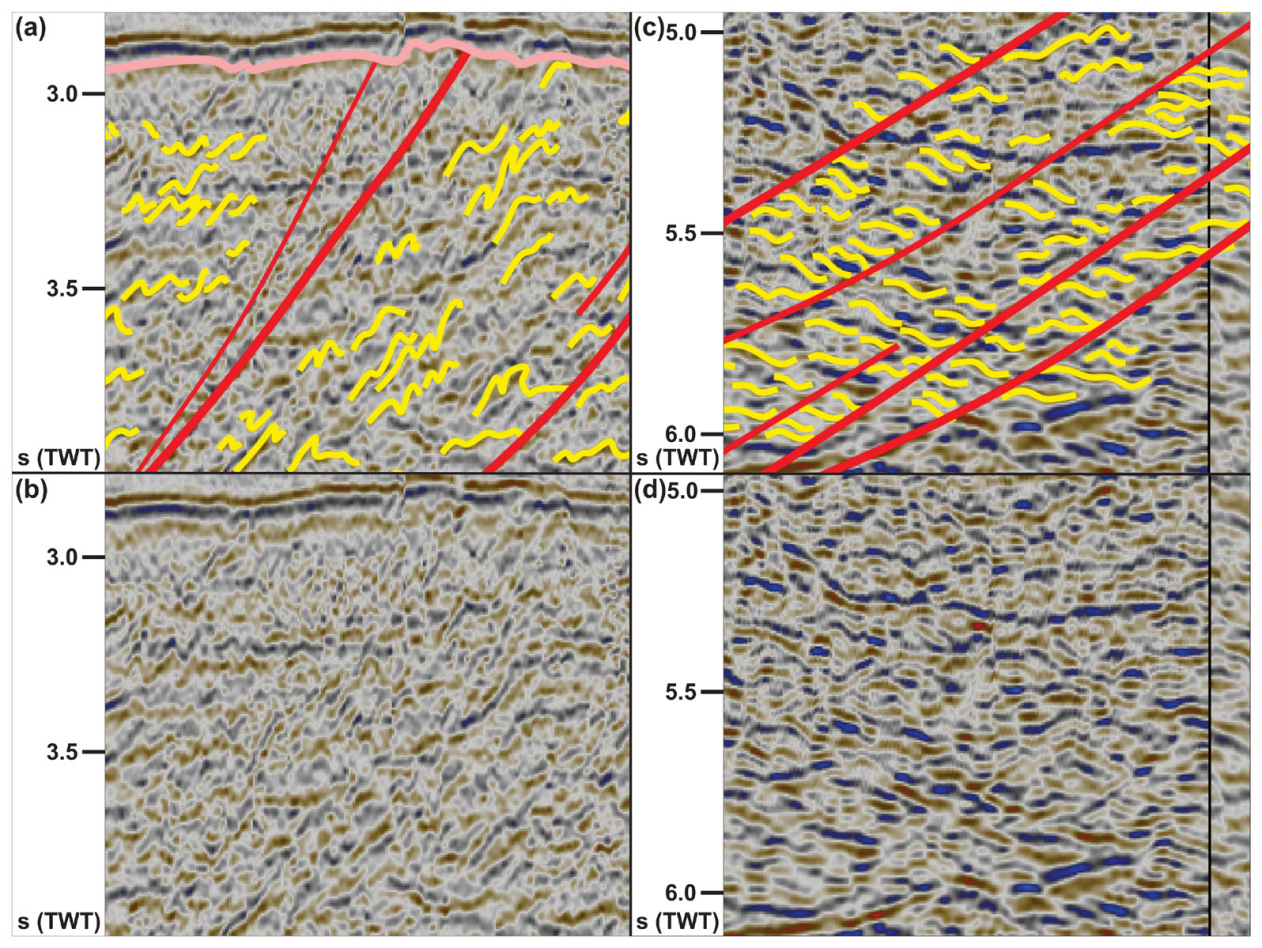
( **a**) Interpreted and ( **b**) uninterpreted zoom in the upper part of the Risen fault zone consisting of asymmetric, north-verging folds and mylonitic surfaces. Notice the rugose morphology of the Top-basement reflection above the Risen fault zone. ( **c**) Interpreted and ( **d**) uninterpreted zoom in the lower part of the Risen fault zone showing down-south extensional duplexes separated by mylonitic shear surfaces. See location and legend in Figure 2.

**Figure 6.**
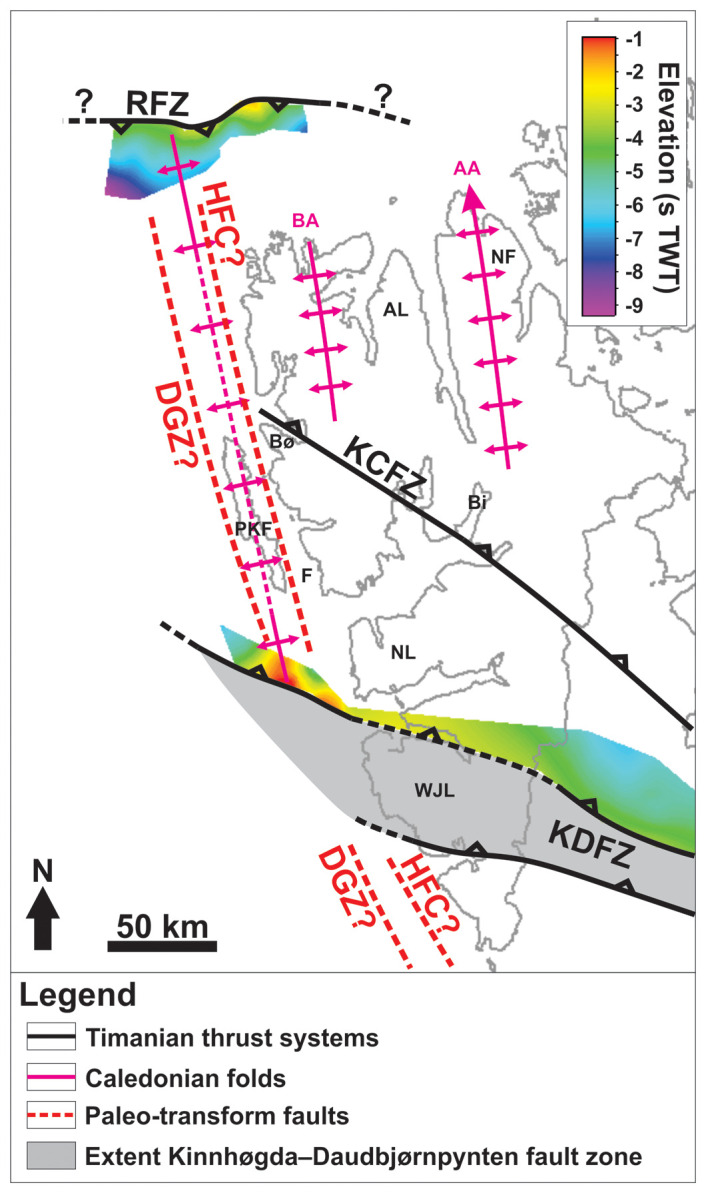
Depth map (in seconds TWT) of the south-dipping Risen fault zone lower envelope. The map shows that the Risen and Kinnhøgda–Daudbjørnpynten fault zones extend well across and past the location of the De Geer Zone and Hornsund Fault Complex west of Spitsbergen. Notice the similar strike and width of the south-plunging folds of the Risen fault zone and of the north-plunging fold of the Kinnhøgda–Daudbjørnpynten fault zone west of Spitsbergen to that of major Caledonian fold structures onshore Spitsbergen (e.g., Bockfjorden Anticline and Atomfjella Antiform). Abbreviations: AA: Atomfjella Antiform; AL: Andrée Land; BA: Bockfjorden Anticline; Bi: Billefjorden; Bø: Brøggerhalvøya: DGZ: De Geer Zone; F: Forlandsundet; HFC: Hornsund Fault Complex; KCFZ: Kongsfjorden–Cowanodden fault zone; KDFZ: Kinnhøgda–Daudbjørnpynten fault zone; NF: Ny-Friesland; NL: Nordenskiöld Land; PKF: Prins Karls Forland; RFZ: Risen fault zone; WJL: Wedel Jarlsberg Land.

**Figure 7.**
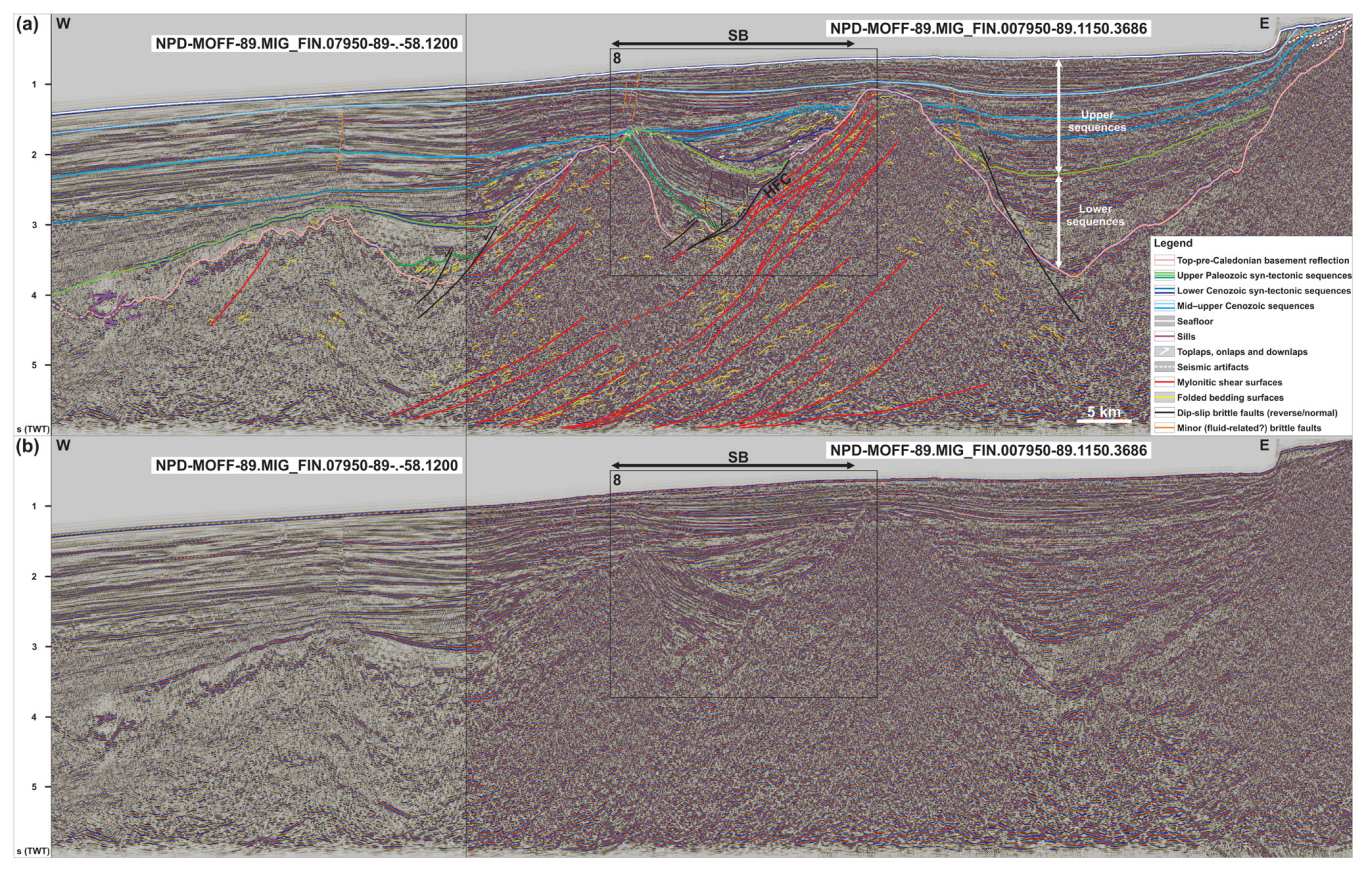
( **a**) Interpreted and ( **b**) uninterpreted E–W-oriented seismic section showing the west-dipping geometry of the Hornsund Fault Complex northwest of Spitsbergen, its relationship to probable top-east Caledonian mylonitic thrust surfaces, and its influence in the formation of post-Caledonian basins and faults. The vertical black line in the data indicates a change of dataset (i.e., intersection of two seismic lines). Location is shown in Figure 1b. Abbreviation: HFC: Hornsund Fault Complex; SB: Sjubrebanken basin.

The structures studied include > 500 m wide and > 150 m thick asymmetric folds, which are within the horizontal and vertical resolution of the data, both at shallow and high depth (≥ 5000 m; Table 1). The vertical resolution of seismic data may be down to 1/32 of the wavelength in places ( Kallweit & Wood, 1982; Li & Zhu, 2000), i.e., down to c. 5 m for 40 Hz frequency (= 6300/40/32), thus further supporting the interpreted structures. The water depth in the study area around Svalbard is about 200–400 m with little variations ( Jakobsson *et al.*, 2012) and, thus, has little influence on the interpreted structures. Seismic velocities for basement rocks in northern Norway from Gernigon *et al.* (2018) suggest that there is little to no vertical exaggeration in the basement rock interval ( Figure 2, Figure 3, Figure 4, Figure 5, Figure 6, and Figure 7). Thus, the geometry of the interpreted basement-seated structures is likely similar to their actual geometry.

Phanerozoic sedimentary successions north of Spitsbergen were studied and extensively described by previous studies (among others, Eiken, 1993; Eiken, 1994; Eiken & Austegard, 1987; Geissler & Jokat, 2004) and are therefore not discussed in details because they are out of scope for the present contribution. Nevertheless, they are briefly mentioned in conjunction with N–S-striking faults and shear zones for which the syn- or post-tectonic character of the strata has implications for the formation and reactivation of the interpreted structures.

The plate reconstruction presented in the discussion was performed using the open-source software GPlates. The rotation file used was updated after Domeier & Torsvik (2014) and Matthews *et al.* (2016).

## Results

### Description

***WNW–ESE- to E–W-striking structures.*** Seismic reflection data reveal the occurrence of two seismic packages of interest displaying moderate-amplitude, asymmetric seismic reflections separated by linear disruption surfaces (i.e., planes/surfaces along which seismic reflections are generally disrupted) within basement rocks north and west of Spitsbergen ( Figure 2, Figure 3, and Figure 4). The package north of Spitsbergen is 5–8 km wide and 1.0–1.5 second (TWT) thick and shows reflections and disruption surfaces dipping dominantly to the south ( Figure 2). The package west of Nordenskiöld Land ( Figure 1b) is 5–12 km wide, 1.0–2.5 second (TWT) thick and displays a general dip to the north-northeast ( Figure 4). The package of south-dipping reflections extends from a depth of ca. 2.5 (locally 2.0) seconds up to 9.0 seconds in the west (TWT) and is bounded by two prominent disruption surfaces that truncate adjacent gently undulating basement reflections ( Figure 2 and Figure 5a–b and Supplement S2 ( Koehl, 2025)). The package west of Nordenskiöld Land extends from a minimum depth of 0.5 second (TWT) just west of the coast to a depth of at least 5.5 second (TWT) in the west ( Figure 4 and Supplement S3 ( Koehl, 2025)).

In N–S- to NNW–SSE-oriented seismic cross sections, the upper part of the package north of Spitsbergen (ca. 2.5 to 5.0 seconds TWT) and the entirety of the package west of Nordenskiöld Land show curving, typically a few hundreds of meters wide reflections with large (curving) amplitude. Within both packages, the curving reflections are asymmetric. North of Spitsbergen, the reflections consist dominantly of long and gently dipping southern limbs and of narrower, more steeply dipping northern limbs as if leaning towards the north ( Figure 2 and Figure 5a–b and Supplement S2 ( Koehl, 2025)). The opposite is true for the package west of Nordenskiöld Land, where asymmetric curving reflections show elongated, gently dipping northern limbs and shorter, steeply dipping southern limbs ( Figure 4 and Supplement S3 ( Koehl, 2025)). Above the described packages, the Top-basement reflection displays a rugose geometry, which differs from its generally smooth character elsewhere ( Figure 2 and Figure 4). The lower part of the package of south-dipping reflections in the north (below 5.0 seconds TWT) displays dominantly Z-shaped reflections (yellow lines in Figure 5c–d) occurring in elongated aggregates, which parallel and are separated from one another by major disruption surfaces (red lines in Figure 2 and Figure 5c–d). The package west of Nordenskiöld Land also displays Z-shaped reflections in places (Supplement S3 ( Koehl, 2025)), but reflections with S-shaped geometries are also observed ( Figure 4).

In E–W-oriented (along-strike) seismic sections, both packages display a gently undulating geometry with a wavelength of c. 10–20 km (e.g., Figure 3). Internal reflections also show gently undulating, open, and rather symmetric geometries (locally slightly asymmetric) with wavelengths in the range of 0.5–1.0 km ( Figure 3). The overall undulating geometry of the two major packages also appears on the depth map, which shows that the 10–15 km wide folds are characterized by a south-plunging geometry for the package northwest of Spitsbergen and by a northern plunge for the package west of Nordenskiöld Land ( Figure 6). Both packages also show a gently undulating geometry in map view and pinch out below the Top-basement reflection in places, with a dominant E–W strike alternating with ENE–WSW and WNW–ESE strikes locally for the northern package, and alternating WNW–ESE- and E–W-striking segments for the package west of Nordenskiöld Land ( Figure 1b and Figure 6).

Seismic cross sections also show the presence of asymmetric curving reflections in basement rocks adjacent to both packages at depth of 2.5 to 3.5 seconds (TWT) north of Spitsbergen ( Figure 2) and 0.7–3.0 seconds (TWT) west of Nordenskiöld Land ( Figure 4). In the north, a notable difference is the opposite vergence of the reflections, i.e., southward-leaning with large, gently dipping northern limbs and short, steeply dipping southern limbs ( Figure 2). These reflections are crosscut by gently north-dipping disruption surfaces across which they are offset and may, in places, be correlated with their offset counterpart. One of these disruption surfaces extends across the Top-basement reflection, showing a minor (a few hundreds of meters) top-south reverse offset, but does not extend into overlying seismic reflections. By contrast, several shallow disruption surfaces are seen to crosscut the Top-basement surface west of Nordenskiöld Land ( Figure 4). Above the major NNE-dipping package, these surfaces show steeply to moderately NNE-dipping, listric geometries, merge at depth with major disruption surfaces within the NNE-dipping package and are associated with reverse and normal offsets of the Top-basement reflection in the south (black lines in Figure 4). Just north of the major package, a few subvertical disruption surfaces are associated with narrow, triangular uplifts and minor vertical offsets of the Top-basement reflections (orange lines in Figure 4). Most of these disruption surfaces die out ca. 0.1 second (TWT) below the seafloor reflection except for one subvertical surface, which extends all the way to the seafloor ( Figure 4). Both the listric and the subvertical disruption surfaces correlate with gentle open folding of the seafloor ( Figure 4).

North of Spitsbergen, the southeastern part of the package of south-dipping reflections is seemingly crosscut by a zone with subvertical disruptions below a 3–5 km wide conical ridge (dotted black lines in Figure 3). Above the Top-basement reflection, the ridge is associated with ca. 0.1 second thick packages of moderate amplitude reflections, which pinch out within 5 km from the ridge giving the ridge and the related pinching out packages a Christmas-tree geometry (dotted white lines in Figure 3).

**Figure 8.**
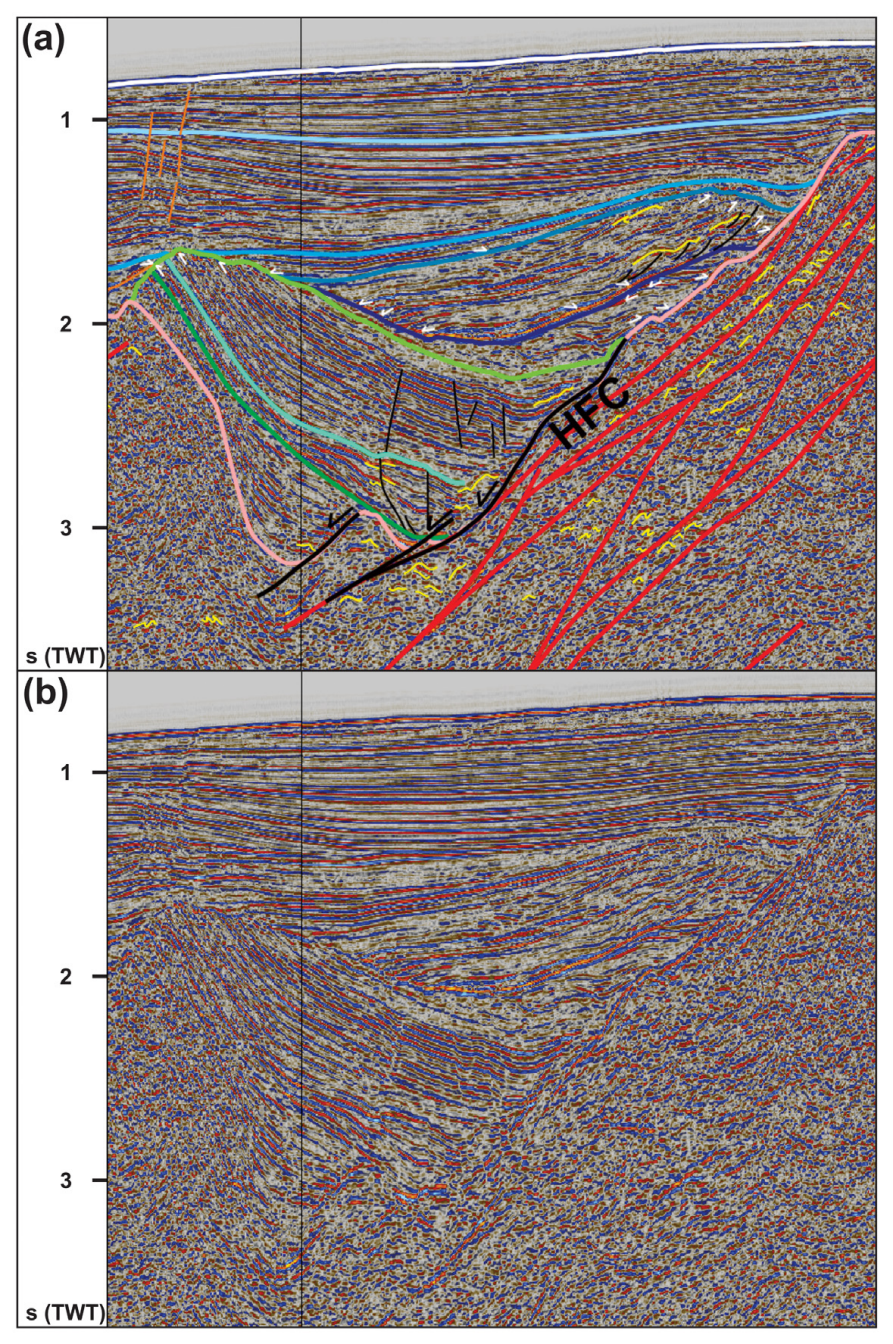
( **a**) Interpreted and ( **b**) uninterpreted zoom in the upper part of the Hornsund Fault Complex consisting of asymmetric, east-verging folds and mylonitic surfaces probably of Caledonian age, and its relationship to the overlying post-Caledonian Sjubrebanken basin. Notice the syn-tectonic (upper Paleozoic?) sedimentary sequences in the lower half of the basin (green lines) and their erosional truncation above a basement high to the west (toplaps symbolized by white half arrows) and onlapping syn-Eurekan early Cenozoic and post-tectonic late–mid-Cenozoic sequences (blue lines). See legend in Figure 7.

***N–S- to NNW–SSE-striking structures.*** E–W-striking seismic cross sections west of Albert I Land in northwestern Spitsbergen show a series of four N–S- to NNW–SSE-striking basins and highs, three of which are fully covered by the data ( Figure 7). The eastern basement high shallows up to near sea level and seismic reflections there are challenging to interpret due to multiples ( Figure 7). Similarly to the south-dipping packages described in the previous sub-section, basement rocks at depths of 1.5–6.0 seconds (TWT) in the two central highs consist of a c. 50 km wide system of numerous moderate-amplitude, curving, asymmetric reflections and associated linear, moderately-dipping disruption surfaces. Both the curving asymmetric reflections and disruption surfaces are dominantly found on the western flank of the two central basement highs ( Figure 7 and Figure 8). There, the curving reflections lean dominantly to the east and the disruption surfaces dip moderately to the west, which roughly parallel the irregular Top-basement reflection ( Figure 7). Some curving reflections with opposite geometries (i.e., leaning towards the west) are observed locally on the eastern flank of the two central basement highs ( Figure 7). The data also show a few small packages of Z-shaped reflections in between major disruption surfaces ( Figure 7). The westernmost basement high displays a rugose Top-basement reflection and is relatively challenging due to a relatively chaotic facies related to noise artifacts, which appear both in basement rocks and overlying sedimentary successions in the western part of the seismic transect ( Figure 7). Nevertheless, some asymmetric curving reflections and disruption surfaces are present ( Figure 7).

Of the basins overlying basement rocks in this area, the easternmost displays an U- to V-like shape. Intra-basinal reflections can be traced for tens of km and are gently curved downwards into a several tens km sag ( Figure 7). While most intra-basinal reflections onlap adjacent basement rocks on the basin flanks, sedimentary units are thicker in the basin center and pinch out towards the basin flanks. In the west, this basin is truncated by a steeply east-dipping disruption surface between depths of 2.0–4.5 seconds, which terminates into basement rocks (TWT).

The central basin is asymmetric and intra-basinal sedimentary units can be mapped in great detail ( Figure 8). This basin is hereby named Sjubrebanken basin. The lower reflection packages consist of dominantly continuous, high-amplitude, gently to steeply east-dipping reflections and thicken eastward towards disruption surfaces localized along the Top-basement reflection (green lines in Figure 7 and Figure 8). In the west, the lower reflection packages steepen and terminate as toplaps truncated unconformably by overlying sedimentary units ( Figure 8). Above their western termination, a few minor, steeply west-dipping disruption surfaces across which seismic reflections are only mildly offset are found ( Figure 8). Most of the upper reflection packages can be traced throughout the entire seismic section and display gentle thickness variations, generally thickening towards the center of basins (blue lines in Figure 7 and Figure 8). In both lower and upper reflection packages, a few curving reflections and small disruption surfaces are found ( Figure 8). The westernmost basin only partly shows in the data and consists of westward-thickening reflection packages ( Figure 7). Locally, high-amplitude positive reflections with U- and X-shaped geometries crosscut reflections in the lowermost part of the basin ( Figure 7).

### Interpretation

The asymmetric and curving reflections in the upper part of the package of south-dipping reflections north of Spitsbergen in seismic cross sections are interpreted as tightly folded bedding surfaces in Precambrian–lower Paleozoic metasedimentary rocks ( Figure 2 and Figure 5a–b). The northward-leaning geometry of individual reflections suggests that they correspond to north-verging folds reflecting top-north thrusting ( Figure 2 and Figure 5a–b and Supplement S2). Conversely, southward-leaning asymmetric reflections within the major NNE-dipping package west of Nordenskiöld Land are interpreted as SSW-verging folds ( Figure 4 and Supplement S3) and east-leaning reflections west of Albert I Land as east-verging folds ( Figure 7). The interpretation is supported by previous seismic studies in the Barents Sea reporting similar reflection geometries ( Koehl, 2020; Koehl *et al*., 2022a; Koehl *et al*., 2023a).

The S- and Z-shaped reflections in the lower part of the package of south-dipping reflections north of Spitsbergen ( Figure 2 and Figure 5c–d) and locally in the NNE-dipping package west of Nordenskiöld Land ( Figure 4) and in the west-dipping system west of Albert I Land ( Figure 7) are interpreted as tightly folded bedding surfaces in metasedimentary rocks offset and stacked onto one another by minor brittle faults. The resulting aggregates of S- and Z-shaped reflections are interpreted as duplex structures ( Boyer & Elliott, 1982; McClay, 1992). Such geometries are not unusual for pre-Caledonian basement rocks in the Barents Sea ( Koehl, 2020; Koehl *et al*., 2022a; Koehl *et al*., 2023a; Koehl & Stokmo, 2024) and onshore Svalbard ( Bergh *et al*., 1997; Bergh *et al*., 2000; Braathen *et al*., 1999a). The dominant Z-like shape for reflection aggregates in the lower part of the south-dipping package north of Spitsbergen and locally within the NNE-dipping package west of Nordenskiöld Land and west-dipping system west of Albert I Land suggest a down-south, down-NNE, and down-west component of extensional movement respectively ( Figure 2, Figure 4, Figure 7, and Supplement S3). In contrast, S-shaped reflections within the package west of Nordenskiöld Land suggest top-SSW contractional movement ( Figure 4).

The disruption surfaces bounding and within the two major packages north of Spitsbergen and west of Nordenskiöld Land truncate the interpreted duplexes and asymmetric folds. They are therefore interpreted as major faults ( Figure 2, Figure 4, and Figure 5). The rugose geometry of the Top-basement reflection above these major packages indicates differential erosion of basement rocks within the two packages ( Figure 2 and Figure 4). This suggests the occurrence of significant rheological contrasts within the packages. A probable cause may be the presence of relatively strong mylonitic shear zones around major faults and slip surfaces alternating with weaker zones of non- to less-mylonitic zones (e.g., Fountain *et al*., 1984; Hurich *et al*., 1985), i.e., reflecting strain partitioning within a major shear zone. Thus, the 5–12 km wide packages are interpreted as major south- and NNE-dipping shear zones. This interpretation is consistent with basement subcrops above shear zones and with the geometry of major shear zone elsewhere ( Collanega *et al*., 2019; Fazlikhani *et al*., 2017; Fountain *et al*., 1984; Koehl *et al*., 2018a; Koehl *et al*., 2022a; Koehl *et al*., 2023a; Lenhart *et al*., 2019; Phillips & McCaffrey, 2019; Phillips *et al*., 2016; Phillips *et al*., 2019). The package north of Spitsbergen is hereby named the Risen fault zone. The package west of Nordenskiöld Land is interpreted as the continuation of the northern flank of the Kinnhøgda–Daudbjørnpynten fault zone. This is supported by the alignment of the shear zone location and matching strike, dip and geometry with the northern edge of the Kinnhøgda–Daudbjørnpynten fault zone in Storfjorden just east of southern Spitsbergen ( Koehl *et al*., 2022a; Figure 1b). As a result, the Kinnhøgda–Daudbjørnpynten fault zone is now believed to extend the entire width (along a N–S axis) of Wedel Jarlsberg Land ( Figure 1b).

The west-dipping disruption surfaces west of Albert I Land show a comparable truncate east-verging asymmetric folds and duplexes ( Figure 7). They are therefore interpreted as east-verging, possibly mylonitic thrusts. Their dominant vergence to the east is comparable to that of Caledonian thrusts onshore western Spitsbergen (e.g., Ohta *et al.*, 1986; Ohta *et al.*, 1995). Thus, they are interpreted as Caledonian thrusts and shear zones.

Comparably, asymmetric, southward-leaning reflections within shallow basement rocks (depth of 2.5–3.5 seconds TWT) and truncating disruption surfaces south of the south-dipping shear zone north of Spitsbergen are interpreted as south-verging folds and top-south brittle (–ductile?) thrusts ( Figure 2). The truncation of the Top-basement reflection by the largest top-south thrust suggests a reactivation of this thrust during a subsequent episode of contraction, possibly in the early Cenozoic during the Eurekan episode as suggested by its truncation of the Top-basement reflection but not of overlying upper Cenozoic sedimentary strata ( Figure 2). Alternatively, this thrust might be younger than all the surrounding structures, but this is considered unlikely because the strong rheological discontinuities at and around the Risen fault zone and other north-dipping thrusts would certainly have been reactivated or overprinted. This would have probably resulted in the truncation of the Top-basement reflection elsewhere prior to the formation of a brand-new thrust.

West of Nordenskiöld Land, the shallow disruption surfaces are interpreted as Cenozoic brittle faults because they crosscut overlying, probably lower Cenozoic ( Blinova *et al*., 2009; Gabrielsen *et al*., 1992) sedimentary rocks and coincide with mild folding of the seafloor ( Figure 4). The listric faults are associated with both reverse and normal offsets of the Top-basement reflection (black lines in Figure 4), therefore suggesting that they correspond to early Cenozoic Eurekan thrusts reactivated as normal faults during the opening of the Fram Strait. The merging geometry of these faults with mylonitic surfaces within the Kinnhøgda–Daudbjørnpynten fault zone suggest that the latter controlled the formation of the former ( Figure 4). By contrast, the subvertical geometry of the brittle faults just north of the Kinnhøgda–Daudbjørnpynten fault zone and the associated triangular uplift and minor or lack of vertical offset of seismic reflections across these faults suggest that they accommodated dominantly strike-slip movement (orange lines in Figure 4).

The gently folded geometry of the Risen and Kinnhøgda–Daudbjørnpynten fault zones and internal symmetric (to mildly asymmetric) reflections in E–W-oriented (along-strike) seismic sections and their undulating geometry in map view suggest reworking of the shear zones into open folds during a subsequent tectonic episode involving contraction (sub-) parallel or slightly oblique to the shear zones ( Figure 3 and Figure 6). This is further discussed in the first chapter of the discussion.

Asymmetric folds also occur in sedimentary rocks west of Albert I Land where they verge to the east, except in the uppermost three sedimentary packages (i.e., up to the grey blue reflection in Figure 7 and Figure 8). Since the only episode of post-Caledonian contraction known in Svalbard is the early Cenozoic Eurekan episode, these east-verging folds and related minor brittle thrusts are interpreted to have formed during this episode. Thus, sedimentary successions above the grey blue reflection in Figure 7 and Figure 8 are interpreted as mid–upper Cenozoic (probably post-Eocene) strata. This is consistent with previous work north of Svalbard (e.g., Geissler & Jokat, 2004) and in agreement with mid–late Cenozoic ages for the shallow sedimentary successions in the area from IODP campaigns ( Hull *et al.*, 1996; Thiede *et al.*, 1995).

The lower reflection packages in the asymmetric sedimentary basins west of Albert I Land (e.g., Sjubrebanken basin) typically thicken towards disruption surfaces localized along the flanks of basement highs. These are interpreted as syn-tectonic sedimentary strata deposited along half-graben-bounding normal faults ( Figure 7 and Figure 8). In the Sjubrebanken basin, these syn-tectonic deposits steepen to the west where they are erosionally truncated at a high angle above the adjacent basement high ( Figure 8). This abrupt termination, the absence or thin character of sedimentary deposits of the lower three sedimentary packages of the upper sequences (i.e., between the light green and common blue reflections in Figure 7 and Figure 8), and the (early Cenozoic Eurekan) folding of the lower two successions of the upper sedimentary sequences suggest a relationship between exhumation of the basement high, folding in sedimentary rocks, and sedimentation as well as a probable significant hiatus between the lower and upper sedimentary sequences ( Figure 7). It is thus proposed that the lower two folded successions of the upper sedimentary sequences were deposited coevally with exhumation of the basement high during early Cenozoic Eurekan contraction (i.e., Paleocene–Eocene). These successions may correlate with successions YP-1 and DB-1 of Geissler & Jokat (2004), which were not penetrated by IODP wellbores.

In contrast, the strata of the lower sequences are bounded by normal faults and were tilted and erosionally truncated prior to deposition of the upper sequences. The Sjubrebanken basin displays a geometry characteristic of extensional half-grabens, including several minor half-graben-bounding normal faults and associated depocenters at the base of the basin (“Initiation” stage), a major early syn-tectonic sequence that thickens towards the main fault (“Interaction and linkage” stage), and a thick, late/post-tectonic sequence (“Through-going fault zone” stage; Gawthorpe & Leeder, 2000). Thus, the lower sequences are interpreted as possible upper Paleozoic, collapse-related strata (probably Devonian–Carboniferous). This differs from the results from Geissler & Jokat (2004) in nearby areas north of Spitsbergen (Yermak Plateau; Figure 1a), who derived the age of the successions from average estimates of the sedimentation rate and thus obtained mid–late Cenozoic ages of all successions. The present interpretation is supported by the reactivation–overprinting of preexisting, basement-seated Caledonian thrusts and shear zones by the interpreted late Paleozoic, half-graben-bounding faults and traces of extensional reactivation of the sub-basin Caledonian thrust system (e.g., Z-shaped duplexes; Figure 7 and Figure 8). It is also supported by the steep tilting and extensive erosion of the lower sedimentary sequences over the basement high ( Figure 7 and Figure 8). It is further supported by the few minor, steeply west-dipping faults in flat-lying mid–upper Cenozoic sedimentary rocks above the erosional truncation of these strata. The local occurrence and limited normal offsets across these faults suggest that they are related to fluid flow rather than tectonic processes. Possible candidates are hydrocarbons from uppermost Devonian–Mississippian coals of the Billefjorden Group ( Figure 8; Cutbill & Challinor, 1965; Cutbill *et al.*, 1976), which crop out onshore western Spitsbergen ( Fairchild, 1982). This local succession may represent the equivalent to the YP-0 succession of Geissler & Jokat ( 2004 their figure 3a) in adjacent areas on the Yermak Plateau. Nonetheless, a connection of the Sjubrebanken basin with the Forlandsundet Graben, which also was eroded, displays a half-graben geometry, and is bounded by a west-dipping normal fault (e.g., Gabrielsen *et al.*, 1992; Schaaf *et al.*, 2020), cannot be completely ruled out. More work on both basins is required to further constrain their formation and development.

It is possible that Devonian (and/or) mid Cenozoic core complex exhumation played a minor role exhuming the basement high and overlying upper Paleozoic strata, as noted for other basement culminations onshore northern and western Spitsbergen (e.g., Braathen *et al.*, 2018; Schaaf *et al.*, 2020). However, there is no trace of typical, core-complex-related, bowed mylonitic detachment near the Top-basement reflection below the upper Paleozoic sedimentary strata. Further work is therefore needed to establish whether such processes were at work or not. By contrast, evidence of Eurekan folds and thrusts in lower Cenozoic sedimentary strata indicate Eurekan contraction as a major driver for exhumation of the basement high west of the Sjubrebanken basin ( Figure 7 and Figure 8).

The easternmost basin west of Albert I Land is not bounded by any major tectonic structure. The V-like geometry of the lowermost part of the basin suggests that basement rocks were eroded possibly by glaciers and/or fluvial systems and that the basin was then passively filled by sediments. It is possible that a normal fault bounds the eastern flank of this basin, the interpretation of which is complicated by multiples in the easternmost basement high ( Figure 7). However, the eastward pinching-out character of the sedimentary rock units in this basin suggests that this is not the case and that the basin simply subsided. It is also possible that an east-dipping normal fault along the Top-basement reflection bounds the basin to the west. However, the lowermost basin strata rather thicken towards the basin center ( Figure 7). Core-complex-related detachment faulting is therefore unlikely for this basin.

The absence of any major structure in the easternmost basin indicates that the only possible candidates to correspond to the Hornsund Fault Complex are the moderately west-dipping normal faults on the western flank of the two central basement highs ( Figure 7 and Figure 8). None of them is steep enough to have accommodated significant strike-slip movement and both are relatively minor faults extending for only a few tens km ( Figure 1b). This is in agreement with previous studies west of Spitsbergen, which remarked that the Hornsund Fault Complex is not imaged as a prominent structure on seismic reflection data ( Austegard *et al.*, 1988; Mann & Townsend, 1989).

In the westernmost basin west of Albert I Land, the high-amplitude positive reflections with X- and U-shaped geometries truncate both basement rocks and upper Paleozoic sedimentary rocks ( Figure 7). The high-positive contrast in acoustic impedance suggests the occurrence of relatively denser, possibly mafic igneous rocks. Their singular X- and U-shaped geometries are typical of saucer-shaped sills, which are common among Early Cretaceous intrusions of the High Arctic Large Igneous Province in central and eastern Spitsbergen (e.g., Senger *et al.*, 2013). The sills are thus interpreted to be Early Cretaceous. It is also possible that they are in fact mid–late Cenozoic and related to the rifting of the Fram Strait. However, igneous rocks related to this tectonic episode are scarce and only a few occurrences of lava flows and plugs have thus far bee reported onshore northern Spitsbergen (e.g., Amundsen *et al.*, 1987; Burov, 1965; Gjelsvik, 1963; Griffin *et al.*, 2012; Hansen *et al.*, 2003; Skjelkvåle *et al.*, 1989). It is therefore more probable that the sills are Early Cretaceous in age.

The relationship of the conical ridge with pinching-out reflection packages within mid-upper Cenozoic sedimentary rocks overlying basement rocks suggest that the ridge consists of material younger than the age of the local Precambrian–lower Paleozoic basement rocks. The Christmas-tree geometry of the ridge and associated pinching-out packages suggest that it may represent a salt or shale diapir with mass transport deposits and carbonate mounds on a diapir’s flanks (e.g., Giles & Rowan, 2012; van Rensbergen *et al.*, 1999), or a volcanic cone with draping lava sequences ( Magee *et al*., 2019; Phillips & Magee, 2020). Based on the geology of nearby onshore areas, the presence of evaporites in metamorphosed pre-Caledonian basement rocks is considered unlikely. However, Miocene lava flows are found at various localities in northern Spitsbergen ( Prestvik, 1978; Skjelkvåle *et al*., 1989). It is therefore probable that the conical ridge and pinching-out packages reflect Miocene magmatism.

## Discussion

### Timing of formation of the margin-oblique shear zones and related structures

The E–W and WNW–ESE strikes and top-north and top-SSW kinematics of internal structures (e.g., north- and SSW-verging folds; Figure 2, Figure 4, and Figure 5a–b) of the two interpreted shear zones north of Spitsbergen and west of Nordenskiöld Land suggest that they unlikely formed during the Caledonian or Eurekan episodes, which resulted in margin-parallel, N–S- to NNW–SSW-striking, dominantly east-verging folds and top-east thrusts (e.g., Bergh & Grogan, 2003; Birkenmajer, 1975; Birkenmajer, 2004; Dallmann *et al*., 1993; Dumais & Brönner, 2020; Gee *et al*., 1994; Hjelle, 1979; Johansson *et al*., 2004; Johansson *et al*., 2005; Lyberis & Manby, 1999; Lyberis & Manby, 2001; Maher, 1988; Maher *et al*., 1986; Maher *et al*., 1989; Maher *et al*., 1997; Manby, 1986; Manby & Lyberis, 2001a; Manby & Lyberis, 2001b; Tessensohn *et al*., 2001a; Tessensohn *et al*., 2001b; Tessensohn *et al*., 2001c; Welbon & Maher, 1992; Witt-Nilsson *et al*., 1998; von Gosen & Piepjohn, 2001; e.g., Figure 7). The shear zones strike (sub-) parallel to Timanian structures in northern Norway, northwestern Russia, the Barents Sea, and Svalbard ( Faehnrich *et al*., 2020; Gabrielsen *et al*., 2022; Herrevold *et al*., 2009; Klitzke *et al*., 2019; Koehl *et al*., 2022a; Koehl *et al*., 2023a; Koehl *et al*., 2023b; Korago *et al*., 2004; Kostyuchenko *et al*., 2006; Lopatin *et al*., 2001; Lorenz *et al*., 2004; Majka *et al*., 2008; Majka *et al*., 2012; Mazur *et al*., 2009; Olovyanishnikov *et al*., 2000; Siedlecka, 1975). In addition, they show a similar geometry (i.e., moderately dipping in seismic cross section and undulating geometry in map view and in along-strike seismic sections; Figure 3 and Figure 6), consist of similar structures (e.g., mylonitic fault surfaces, duplexes, asymmetric folds and minor thrusts), and are located at a similar depth (i.e., c. 0.5–9.0 s TWT) as most Timanian thrusts in the Barents Sea and Svalbard. It is therefore probable that both shear zones formed during the Timanian Orogeny in the late Neoproterozoic. This is notably supported by the alignment of the shear zone west of Nordenskiöld Land with the northern edge of the Kinnhøgda–Daudbjørnpynten fault zone in Storfjorden in the Barents Sea ( Figure 1b and Figure 6). It is also supported by their dominantly ductile character suggesting that they formed at deeper crustal level than they are currently located, and contrasts with the brittle character of Eurekan thrust faults in the area ( Figure 2) and nearby onshore areas ( Bergh *et al.*, 2000; Piepjohn *et al.*, 2001).

The undulating geometry of the Timanian shear zones both in map view and in along-strike seismic sections suggests folding during a post-Timanian episode involving E–W-oriented contraction. This is consistent with the largely accepted occurrence of the Caledonian Orogeny in Svalbard, which partly reworked Timanian thrusts and shear zones into N–S-striking folds in northern Norway, the Barents Sea, and Svalbard ( Gabrielsen *et al*., 2022; Koehl *et al*., 2022a; Koehl *et al*., 2023a; Siedlecka & Siedlecki, 1971). Notice the coincidence along a N–S- to NNW–SSE-trending axis of the wide, south- and north-plunging anticlines respectively of the Risen and Kinnhøgda–Daudbjørnpynten fault zones and of Prins Karls Forland, which may very well be part of the same or a nearby related Caledonian anticline ( Figure 6). This is further supported by the presence of a comparably large (c. 50 km wide) Caledonian thrust system within the two central basement highs west of Albert I Land ( Figure 7). Further reworking and overprinting occurred during the Eurekan episode in the early Cenozoic and in the late Cenozoic during rifting. Eurekan contractional deformation is suggested by the minor reverse offsets (a few hundreds of meters) of the Top-basement reflection by a north-dipping brittle thrust northwest of Spitsbergen ( Figure 2) and by listric reverse faults west of Nordenskiöld Land ( Figure 4). Late Cenozoic rift-related overprinting is supported by normal offsets by listric faults and strike-slip faulting west of Nordenskiöld Land ( Figure 4). There, the coincidence of the strike-slip faults with gentle folding of the seafloor and the propagation of one of them up to the seafloor reflection suggest recent strike-slip movement. The location of these faults and their strike coincide and align with that of the Molloy fault zone ( Figure 1a–b). Since the minor strike-slip faults die out to the west (Supplement S3), they are not directly linked with the Molloy fault zone. Nevertheless, the 60 km wide, hundreds of kilometers long Kinnhøgda–Daudbjørnpynten fault zone represents a major discontinuity in the crust and it is therefore probable that it controlled the formation and NNE-dipping geometry (e.g., Koehl *et al*., 2021; Thiede *et al*., 1990) of the Molloy fault zone in the late Cenozoic. A similar controlling relationship was recently proposed for the Timanian Kongsfjorden–Cowanodden fault zone and the Spitsbergen fault zone based on new earliest Oligocene U–Pb ages for syn-tectonic carbonate cement along strike-slip fault segments of the former in west Spitsbergen ( Koehl & Mottram, 2024) and on recent earthquakes along the former ( Koehl *et al.*, 2025a awaiting peer review; Figure 1a–b).

The study area north of Spitsbergen was previously suggested to consist of a U-shaped Devonian collapse basin based on seismic refraction data ( Ritzmann & Jokat, 2003). The northern flank of the basin (see their Figure 8) coincides with the location c. 50 km north of Spitsbergen and mimics the south-dipping geometry of the Risen fault zone (depth of ca. 2.5–3.0 seconds TWT; Figure 2). The E–W trend of the basin does not fit that of Devonian basins in Svalbard, e.g., N–S-striking Andrée Land and Raudfjorden basins in northern Spitsbergen ( Braathen *et al*., 2018; Braathen *et al*., 2020; Burov & Semevskij, 1979; Dallmann & Piepjohn, 2020; Friend & Moody-Stuart, 1972; Friend *et al*., 1966; Friend *et al*., 1997; Gee & Moody-Stuart, 1966; Manby & Lyberis, 1992; McCann, 2000; Murascov & Mokin, 1979). In addition, the overall U-shaped (folded?) geometry of the basin is not compatible with that of a Devonian basin in Svalbard because of the lack of a major N–S-oriented contractional episode after the Timanian Orogeny. Notably, recent studies suggested that the Late Devonian Svalbardian Orogeny did not occur in Svalbard ( Berry & Marshall, 2015; Koehl *et al*., 2022b; Lindemann *et al*., 2013; Marshall *et al*., 2015; Newman *et al*., 2019; Newman *et al*., 2020; Newman *et al*., 2021; Scheibner *et al*., 2012). It is therefore more probable that the basin north of Spitsbergen consists of pre-Caledonian metasedimentary rocks, which were folded during the Timanian and Caledonian orogenies. Nevertheless, post-Caledonian collapse may have occurred along the inherited Timanian Risen fault zone as indicated by the extensional duplexes within the shear zone ( Figure 2 and Figure 5c–d).

West of Nordenskiöld Land, part of the interpreted continuation of the Kinnhøgda–Daudbjørnpynten fault zone was previously interpreted as an extensional detachment crosscut by the Hornsund Fault Complex to the west ( Blinova *et al*., 2009). This is in agreement with the interpreted extensional reactivation of the shear zone (e.g., Z-shaped extensional duplexes; Figure 4). However, although previous studies did partly notice the uplift of the Top-basement reflection along the shear zone (see WNW–ESE-striking ridge within the Bellsund Graben in Blinova *et al*., 2009 their figure 11), they did not recognize evidence of top-SSW contractional deformation within the shear zone ( Figure 4). In addition, although the shear zone is partly eroded to the west in the hinge of the major north-plunging anticline ( Figure 6), it continues westwards below Cenozoic sedimentary rocks (Supplement S3), across the location of the Hornsund Fault Complex and De Geer Zone ( Figure 1b and Figure 6). Blinova *et al*. (2009). Previous works also identified minor strike-slip faults in the area, but they ascribed them E–W rather than WNW–ESE strikes ( Blinova *et al.*, 2009).

### Implications for the De Geer Zone and plate tectonic reconstructions

The De Geer Zone and its main segment, the Hornsund Fault Complex, are believed to run ≤ 50 km west of Spitsbergen and to continue farther north along the western edge of the Yermak Plateau (e.g., Faleide *et al*., 2008; Geissler & Jokat, 2004) or to step or bend to the east onto the Yermak Plateau ( Kristoffersen *et al*., 2020). The present study places the Hornsund Fault Complex c. 50 km west of the coastline of Albert I Land in northwestern Spitsbergen ( Figure 7 and Figure 8). However, this fault and other nearby N–S- to NNW–SSE-striking faults and basins (e.g., Sjubrebanken basin) extend only a few tens km and die out north of Prins Karls Forland and south of the Risen fault zone ( Figure 1b). Such a limited extent indicates that they are local structures and accommodated limited movement.

The occurrence of two undisrupted, late Neoproterozoic, WNW–ESE- to E–W-striking shear zones (Risen and Kinnhøgda–Daudbjørnpynten fault zones) extending at least 80 km west of the coastline of northwest and west of Spitsbergen and not showing any sign of lateral or vertical offset ( Figure 1b, Figure 2, Figure 4, and Figure 6, and Supplements S2 and S3) unambiguously indicates that hundreds of kilometers dextral movements along the De Geer Zone and related faults like the Hornsund Fault Complex did not occur. This suggests that the De Geer Zone, which was largely speculated from the N–S-trending and linear morphology of the western Barents Sea–Svalbard and conjugate northern Greenland margins ( De Geer, 1926; du Toit, 1937; Harland, 1961; Harland, 1967; Harland, 1969; Holtedahl, 1936; Horsfield & Maton, 1970; Wegmann, 1948) does not exist, and that its main fault segments, the Hornsund Fault Complex, Knølegga Fault, and Senja Fracture Zone ( Figure 1a), most likely accommodated vertical fault movements.

This is supported by the listric, moderately dipping geometry of the segment Hornsund Fault Complex west of Albert I Land ( Figure 7 and Figure 8) and elsewhere west of Svalbard ( Austegard *et al*., 1988; Eiken, 1994; Geissler & Jokat, 2004). Notably, Austegard *et al*. (1988) reported that all the structures west of Svalbard are extensional and that there are only very few occurrences of strike-slip movements. It is also supported by the lateral disconnection and/or segmentation of the Hornsund Fault Complex west of Svalbard as shown by the limited (a few tens km) N–S extent of the segment mapped west of Albert I Land ( Figure 1b). In addition, the only sparse evidence potentially indicating lateral movement is conflicting. For example, the possible sinistral strike-slip sense of shear indicated by right stepping geometries of margin-parallel brittle faults ( Eiken & Austegard, 1987) contrast with the major component of dextral strike-slip tectonics required for the commonly proposed sheared/transform margin model of the De Geer Zone ( du Toit, 1937; Faleide *et al*., 1993; Harland, 1961; Harland, 1967; Harland, 1969; Horsfield & Maton, 1970; Lepvrier, 1990; Lepvrier & Geyssand, 1985; Steel & Worsley, 1984; Steel *et al*., 1981; Steel *et al*., 1985; Wegmann, 1948). This is consistent with our interpretation of a minor extent and general lack of lateral movement along N–S-striking structures and with that of most previous offshore studies along the western Barents Sea–Svalbard margin (e.g., Eiken, 1994; Riis & Vollset, 1988).

Moreover, previous studies on other fault segments of the presumed De Geer Zone show that the Knølegga Fault accommodated exclusively normal movements in the Cenozoic ( Gabrielsen *et al.*, 1990; Lasabuda *et al.*, 2018). For example, the negative flower structure along the Hornsund Fault Complex off southern Spitsbergen ( Bergh & Grogan, 2003; Lasabuda *et al.*, 2018 their figure 8) corresponds to a zone with low reflectivity and diffraction, probably related to the presence of magmatic intrusions (e.g., saucer-shaped sills and dykes) related to the nearby Vestbakken volcanic province ( Figure 1a). On both sides of this zone, all brittle faults are listric and bound graben and horst structures ( Bergh & Grogan, 2003; Lasabuda *et al.*, 2018), and Bergh & Grogan (2003) argued for dominantly extensional movements and local strike-slip movements along the Hornsund Fault Complex (see line 761230-93 in their figure 11).

Farther south, the Senja Fracture Zone is listric and, thus, rather resembles a normal fault instead of a transform fault (e.g., Indrevær *et al.,* 2013 their figure 7a–b; Kristensen *et al.*, 2017 their figure 8). Furthermore, recent analysis of high-resolution magnetic data suggests that the Proterozoic Bothnian–Senja Fault Complex, which was thought to have been reactivated as a major strike-slip fault by the Senja Fracture Zone, instead corresponds to major late Paleoproterozoic folds with no trace of tens–hundreds km strike-slip movement ( Indrevær *et al.*, 2013; Koehl *et al.*, 2019).

Hundreds of kilometers of lateral movements along the De Geer Zone are not required to explain the geometry of the Svalbard and Greenland margins and the opening of the Fram Strait. Firstly, half of the distance Svalbard moved away from Greenland in the Cenozoic (c. 200 km) was accommodated by lateral movements along the two, c. 200 km long, NW–SE-striking transform faults in the Fram Strait, the Molloy and Spitsbergen fault zones ( Crane *et al*., 1982; Johnson & Eckhoff, 1966; Myhre & Thiede, 1995; Thiede *et al*., 1990). Secondly, other mechanisms may very well account for the remaining 200 km movements. Among others, the reactivation of dominantly top-SSW Timanian thrusts during the Eurekan episode (e.g., Koehl, 2020; Koehl, 2021; Koehl *et al*., 2022a; present study, Figure 2 and Figure 4) and associated folding along a WNW–ESE-trending axis in the early Cenozoic, e.g., in the Sørvestnaget Basin ( Kristensen *et al*., 2017), north of the Loppa High ( Koehl *et al*., 2023a), and north of Svalbard ( Figure 2), support such a claim. Preliminary results indicate that at least 150 km of post-Caledonian N–S shortening may have been accommodated by reactivated/reworked Timanian thrusts ( Koehl, 2020). However, more work is needed to refine this early estimate as more Timanian thrusts and related margin-oblique structures are being discovered (e.g., Risen fault zone). Nonetheless, the present results suggest major revisions in all Phanerozoic plate reconstructions for Arctic regions (e.g., Faleide *et al*., 2008; Nemcok *et al*., 2016) also because it suggests that the continent–ocean boundary in the Fram Strait is located at least 80–90 km to the west of Spitsbergen.

**Figure 9.**
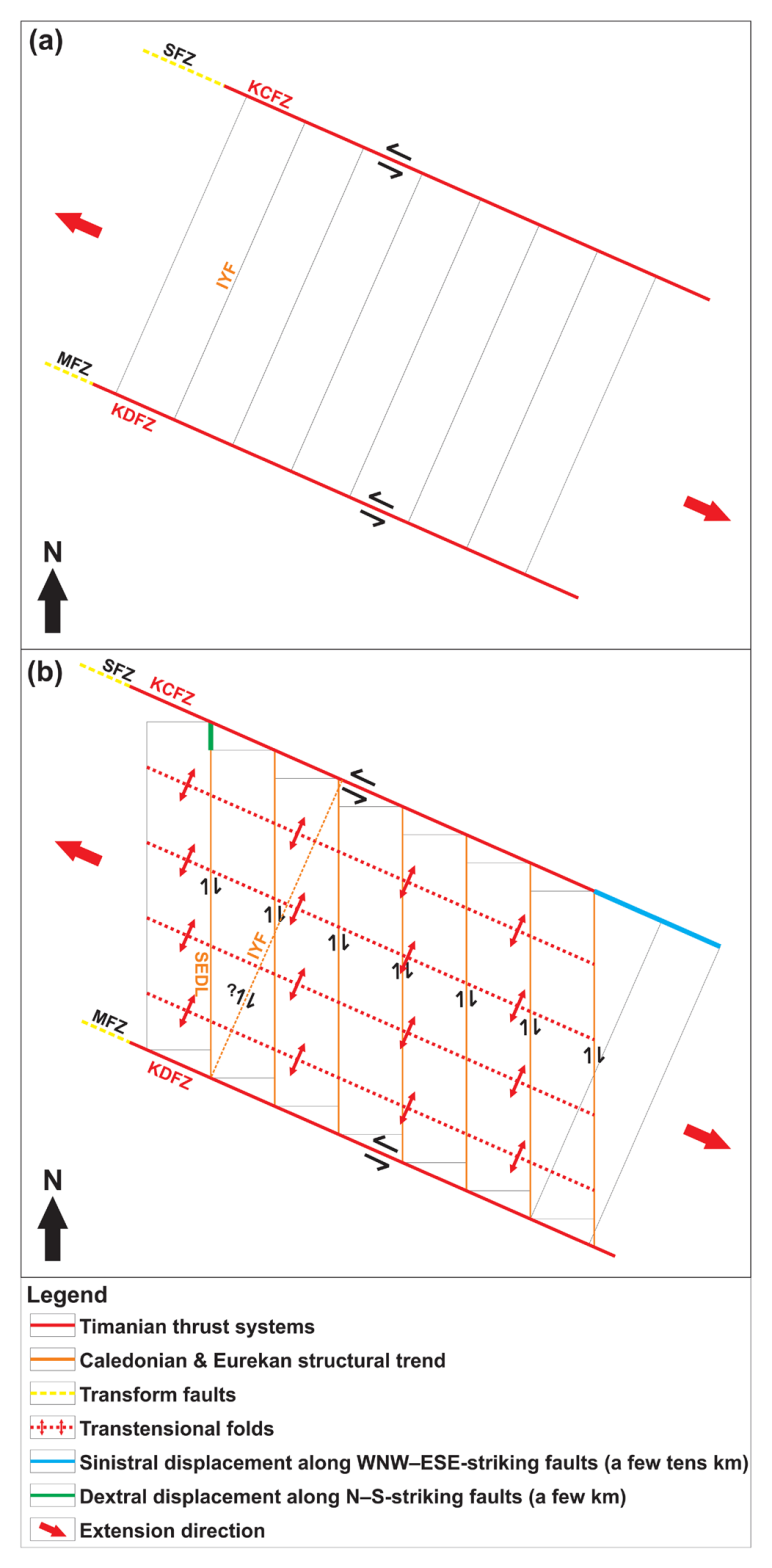
Schematic model of the interaction of WNW–ESE-striking Timanian faults and N–S-striking Caledonian and Eurekan faults in Svalbard during the opening of the Fram Strait. ( **a**) Inherited, late Neoproterozoic Timanian thrust systems such as the Kinnhøgda–Daudbjørnpynten and Kongsfjorden–Cowanodden fault zones (KDFZ and KCFZ; red lines) are reactivated as sinistral strike-slip faults during oblique extension and localize the formation of major transform faults, the Spitsbergen and Molloy fault zones (SPZ and MFZ; dashed yellow lines). ( **b**) Preexisting NNE–SSW-striking (e.g., Caledonian and Eurekan) structural grain and fabrics are reactivated as local dextral strike-slip faults (orange lines) to accommodate tectonic adjustments during NW–SE-oriented extension and NW–SE-striking transform faulting. WNW–ESE-striking transtensional folds form parallel to the extension direction and to reactivated Timanian thrust systems. The green line shows the amount of dextral movement along N–S-striking faults, i.e., a few km up to 10 km, and the blue line sinistral movement along WNW–ESE-striking faults, which is in the order of a few tens of km. Notice how this tectonic setting may give the illusion that the main transform fault strikes N–S instead of NW–SE due to the relatively larger number of N–S-dextral faults in the area. This impression is reinforced if the main few NW–SE-striking faults do not crop out or if related outcrops are of poor quality. Abbreviations: IYF: Isfjorden–Ymerbukta Fault; KCFZ: Kongsfjorden–Cowanodden fault zone; KDFZ: Kinnhøgda–Daudbjørnpynten fault zone; MFZ: Molloy fault zone; SEDL: Svartfjella–Eidembukta–Daudmannsodden Lineament; SFZ: Spitsbergen fault zone.

**Figure 10.**
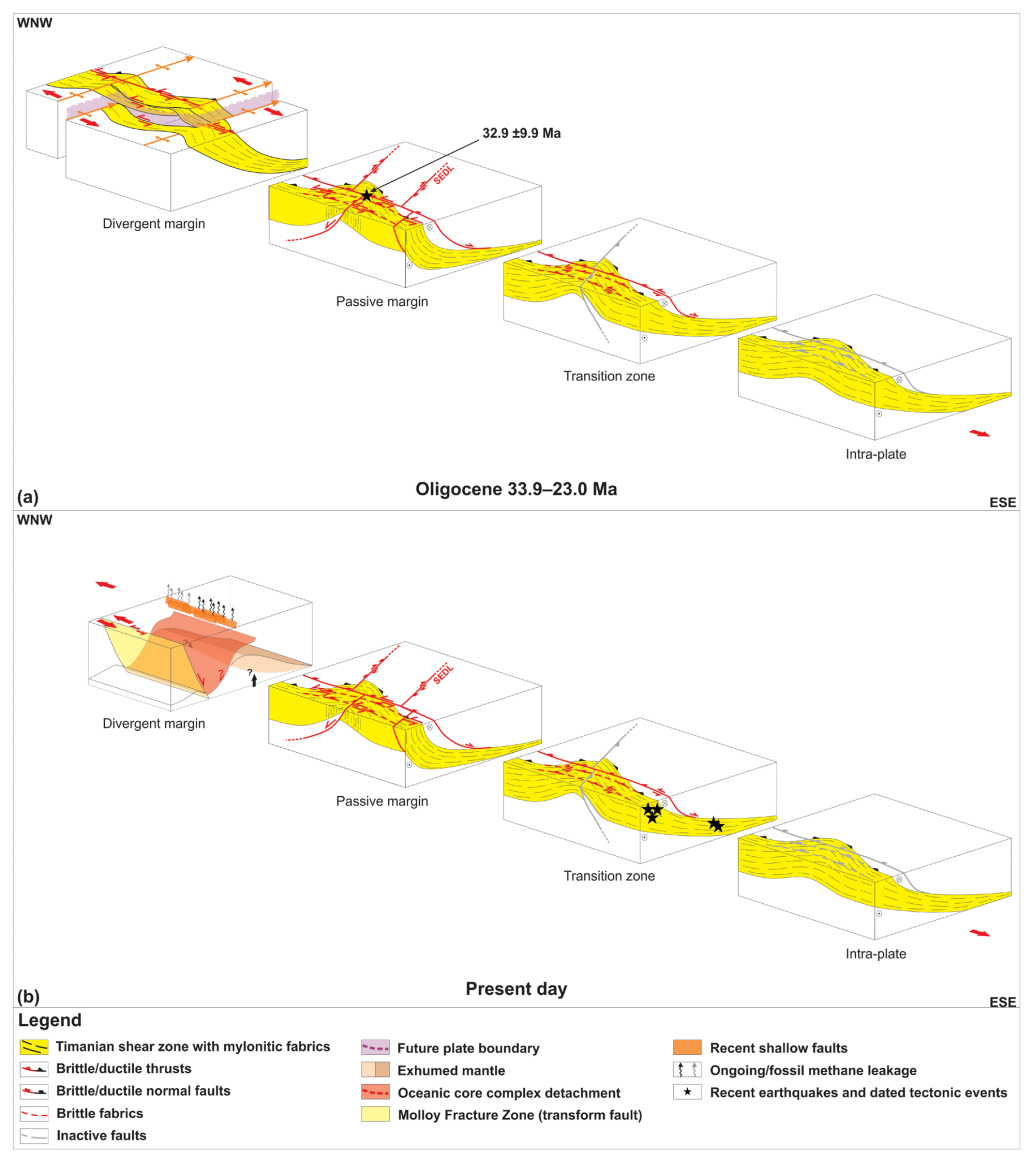
Schematic model of the breakup of the Fram Strait and tectonic evolution of the Svalbard transform margin. ( **a**) WNW–ESE-striking Timanian thrust systems area reactivated as normal–sinistral strike-slip, proto-transform faults (e.g., 32.9 ±9.9 Ma U–Pb on synkinematic carbonate from Koehl & Mottram, 2024). Timanian thrust systems control the breakup and localize mantle exhumation and the formation of margin-oblique core complexes and related detachments near the future plate boundary. ( **b**) NW–SE-striking transform faults form along major Timanian thrust systems and margin-oblique core complexes are only mildly active or inactive. Continued normal–sinistral strike-slip movements occur along inherited Timanian thrust systems in nearby areas (e.g., recent earthquakes in transition zone; Koehl *et al.*, 2025a awaiting peer review). Modified after Koehl *et al.* ( 2025 awaiting peer review). Abbreviations: SEDL: Svartfjella–Eidembukta–Daudmannsodden Lineament.

The newly mapped Timanian thrust systems north and west of Svalbard ( Figure 1b, Figure 2, Figure 3, Figure 4, Figure 7) probably controlled the formation of currently active NW–SE-striking transform faults in the Fram Strait, the Spitsbergen and Molloy fault zones ( Figure 1a–b; Koehl *et al.*, 2021; Koehl & Mottram, 2024). Recent earthquakes around Svalbard indicate that Timanian thrust systems are currently accommodating normal–sinistral movements ( Koehl *et al.*, 2025a awaiting peer review). A new tectonic model in which Timanian thrust systems accommodate most of the transform is therefore proposed ( Figure 9a–b and Figure 10a–b). As a result of sinistral movements along WNW–ESE-striking Timanian faults, N–S-striking Caledonian and Eurekan are reactivated as minor dextral strike-slip faults ( Figure 9b and Figure 10a–b). This model is supported by calcite slickenfibers indicating sinistral-normal movement along WNW–ESE-striking faults and dextral movement along NE–SW-striking faults in the mid–late Cenozoic (ca. 41–13 Ma) in Precambrian basement and lower–mid Cenozoic sedimentary rocks western Spitsbergen ( Haaland *et al.*, 2024; Kleinspehn & Teyssier, 2016). This is consistent with structural field mapping onshore southern Spitsbergen of NNE–SSW- to NNW–SSE-striking faults with exclusively normal and reverse sense of shear and margin-oblique, ENE–WSW- to WNW–ESE-striking strike-slip faults ( Bergh & Grogan, 2003 their figures 3a and 5). In addition, open folds in lower–mid Cenozoic sedimentary rocks interpreted as transtensional folds strike dominantly WNW–ESE ( Kleinspehn & Teyssier, 2016; Schaaf *et al.*, 2020), i.e., parallel to the extension direction proposed in the present model and parallel to the sinistrally-reactivated Timanian thrust systems ( Figure 9b). The present model is also supported by the repeated reactivation of Timanian thrust systems (partly) as sinistral strike-slip faults in the Phanerozoic (e.g., Koehl, 2020; Koehl *et al.*, 2022a; Koehl *et al.*, 2023a; Koehl & Mottram, 2024; Mazur *et al.*, 2009; von Gosen & Piepjohn, 2001; Ziemniak *et al.*, 2022) and by indications of dextral movements along N–S- to NNE–SSW-striking faults onshore Spitsbergen (e.g., Bergh *et al.*, 1997; Harland & Horsfield, 1974; Maher *et al.*, 1997) and Prins Karls Forland (e.g., Lepvrier, 1990).

**Figure 11.**
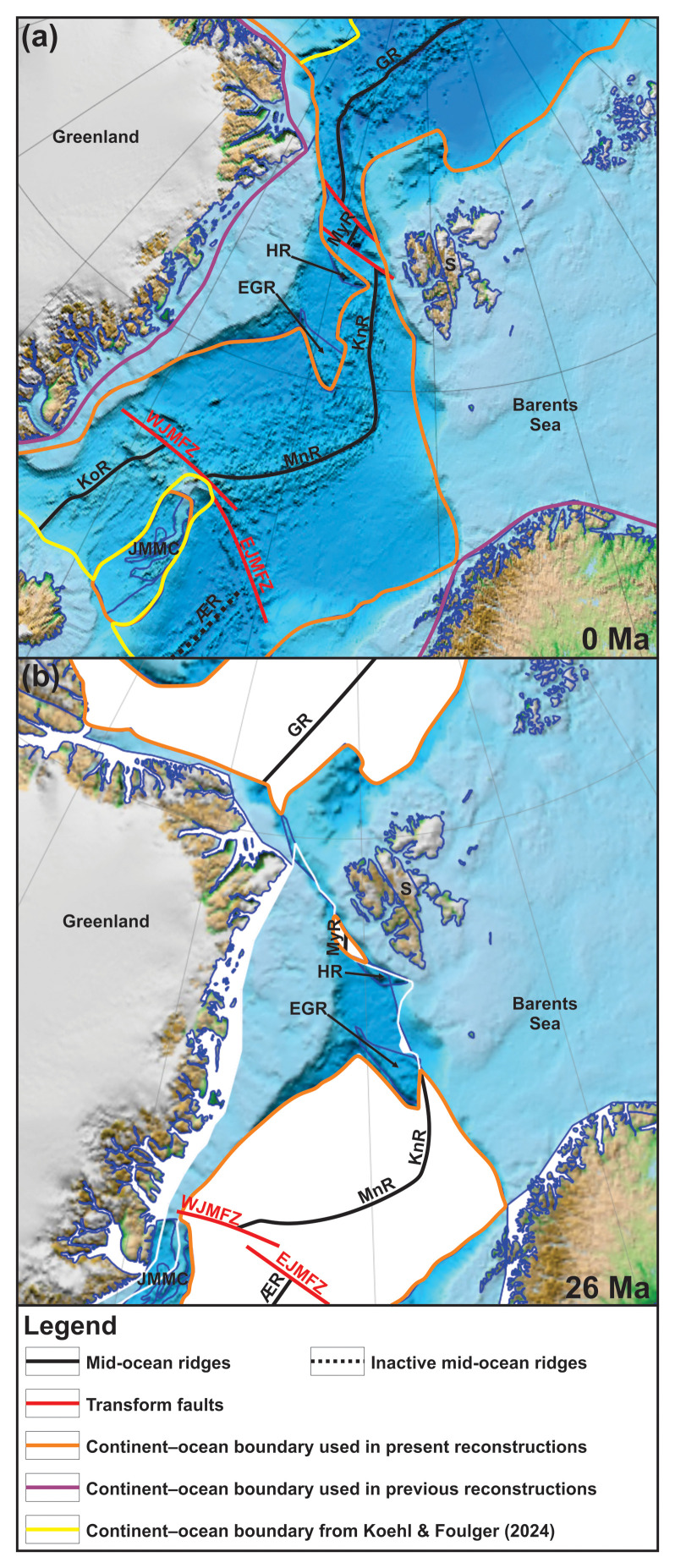
Plate tectonic reconstruction of the opening of the Fram Strait. ( **a**) Current configuration showing the contrast between the continent–ocean boundary used in the present reconstruction (orange lines) and that used in previous reconstructions (purple lines). The present continent–ocean boundary includes blocks previously assumed to be microcontinents, the Hovgård Ridge and East Greenland Ridge, which are now known to be fully attached to Greenland ( Dumais *et al.*, 2021). ( **b**) Reconstruction at the onset of breakup in the Fram Strait (ca. 24–26 Ma) showing how the North Atlantic–Arctic rift stepped sideways and bypassed the thickened continental crust of the oblique Timanian Orogen in Svalbard and the Barents Sea. Basemap in ( **a**) and ( **b**) is from Amante & Eakins ( 2009; CC-0). The rotation file was modified after Domeier & Torsvik (2014) and Matthews *et al.* (2016) and is available from the Extended data on DataverseNO ( Koehl, 2025). Abbreviations: EGR: East Greenland Ridge; EJMFZ: East Jan Mayen fault zone; GR: Gakkel Ridge; HR: Hovgård Ridge; JMMC: Jan Mayen Microcontinent Complex; KoR: Kolbeinsey Ridge; KnR: Knipovich Ridge; MoR: Mohns Ridge; MyR: Molloy Ridge; S: Svalbard; WJMFZ: West Jan Mayen fault zone; ÆR: Ægir Ridge.

Extrapolating the schematic fault-block model from Figure 9 to Spitsbergen, the maximum amount of lateral movement along N–S-striking faults is in the order of 10 km. This is not cumulative because, in this model, N–S-striking faults are simply accommodating local tectonic adjustments in between major WNW–ESE-striking discontinuities in the crust (Timanian thrust systems) and are segmented (e.g., Hornsund Fault Complex west of Spitsbergen, which dies out north of Prins Karls Forland and south of the Risen fault zone; Figure 1b). Since the blocks in Figure 9 are schematic and rocks deform more complexly, i.e., partitioning deformation and distributing displacement along more structures, this is an upper estimate and dextral offsets of a few km are more likely. This is in agreement with estimates from previous studies along N–S-striking dextral faults in western Spitsbergen, e.g., N–S-striking Svartfjella–Eidembukta–Daudmannsodden Lineament in Oscar II Land ( Maher *et al.*, 1997; Figure 1b) and NNE–SSW-striking Isfjorden–Ymerbukta Fault ( Bergh *et al.*, 1997; Braathen *et al.*, 1999a; Harland & Horsfield, 1974).

This model does not require hundreds km dextral transform movements between Greenland and Svalbard. It does not require a (thus far unexplained) change in plate kinematics at breakup at ca. 24 Ma, when the NW–SE-striking Molloy and Spitsbergen fault zones are supposed to have taken over transform motions for the N–S-striking De Geer Zone (e.g., Doré *et al.*, 2015; Faleide *et al.*, 2008). Instead, the main tectonic stress direction may have remained the same ( Figure 9a–b and Figure 10a–b). Restoring Greenland and Svalbard prior to the opening of the Fram Strait would thus come down to closing the oceanic crustal domain at the Knipovich Ridge (and possibly at the Molloy Ridge) along the two NW–SE-striking transform faults, the Spitsbergen and Molloy fault zones ( Figure 1a). Since new high-resolution magnetic data have shown that the oceanic crustal domain between Greenland and Svalbard is relatively narrow (c. 100–200 km; Dumais *et al.*, 2021; see ocean–continent boundary in Figure 1a and Figure 11a), the first step would be relatively simple and involves restoring the Hovgård Ridge ( Figure 1a), where a comparable Timanian thrust was mapped on seismic reflection data ( Koehl, 2020), to just south of Spitsbergen ( Figure 11b). To proceed with closing the Arctic Ocean and northeastern Atlantic Ocean, one would then restore the Timanian Orogen in the Fram Strait to its pre-rift crustal thickness (≥ 40 km), while closing the remaining oceanic crustal domains at the Gakkel and Mohns ridges ( Figure 11b) using deformable plates. The position of Svalbard relative to Greenland would however not have significantly changed from the opening of the Fram Strait at ca. 24 Ma to the end of the Timanian Orogeny at ca 550 Ma ( Figure 11b).

As a result of the proposed restoration, the Svalbard Archipelago would have lain c. 200 km closer to Greenland prior to the opening of the Fram Strait, i.e., east rather than north of Greenland as suggested by previous correlations ( Harland, 1967; Harland, 1969; Jones *et al.*, 2016; Jones *et al.*, 2017; Majka *et al.*, 2021; Piepjohn *et al.*, 2016; Figure 11a–b). This configuration ( Figure 11b) is likely inherited from the Timanian Orogen, traces of which have been found in northern Greenland ( Estrada *et al.*, 2018a; Rosa *et al.*, 2016), and has, thus, likely persisted since the end of the Timanian Orogeny in the latest Neoproterozoic to mid–late Cenozoic extension. Jones *et al.* (2016) have argued that the occurrence of thin volcanic ash layers probably erupted in Kapp Washington in northern Greenland and Ellesmere Island in Arctic Canada ( Figure 1a) in lower Cenozoic strata in central Spitsbergen suggested close proximity of Svalbard with these two volcanic centers. However, volcanic ash may travel over large distances (> 2000 km) and a single ash bed may cover broad areas. For example, the Lava Creek ash bed was erupted from Yellowstone in the Pleistocene and is found all over Texas and western Louisiana, i.e., up to 2300 km from the volcanic center ( Izett & Wilcox, 1982). This distance is larger than that between the volcanic centers in Ellesmere Island and northern Greenland and the ash layers in central Spitsbergen at present (respectively 1900 and 1200 km). In the present model, Svalbard is interpreted to have been located some 200 km closer to both volcanic centers in the early Cenozoic, thus further reducing the distance of Svalbard with the volcanic centers. The proposed model is therefore in agreement with an origin in northern Greenland and Ellesmere Island for the ash layers in central Spitsbergen.

The results of the present study thus suggest that the current plate-tectonic models for the opening of the Fram Strait should be updated with new fault lines and kinematics. The present study shows the danger of using mostly local onshore structural fieldwork in deeply eroded Arctic areas like Svalbard to resolve regional tectonic issues. Such biases are illustrated in Koehl & Allaart (2021), whose work shows that the Billefjorden Fault Zone, although representing a major tectonic discontinuity at a local scale (tens of kilometers long fault with hundreds of meter-scale displacement), does not represent a major regional tectonic boundary as previous thought (e.g., Harland, 1969; Harland *et al*., 1992). This also applies to the De Geer Zone, which was largely supported by local structural field data onshore Spitsbergen (e.g., Bergh *et al.*, 1997; Braathen *et al.*, 1999a; Harland & Horsfield, 1974; Maher *et al.*, 1997), but not by regional seismic studies ( Austegard *et al*., 1988; Eiken, 1994; Riis & Vollset, 1988).

The study also calls for a serious reconsideration of all major faults inferred from indirect observations, generally as necessities to make up for paleogeographic reconstructions shortcomings, rather than observed on specific datasets. An example is the Wegener Fault, a thousand of kilometer-long sinistral strike-slip fault inferred between Ellesmere Island and northwestern Greenland in the Nares Strait, which was proposed solely based on the physiographic morphology of the area, i.e., the linear geometry of the Nares Strait and tentative lateral offset of rock units on either side of the strait ( Taylor, 1910). Convincing evidence from both geophysical datasets (e.g., gravimetric and aeromagnetic anomaly maps) and field mapping show that the bedrock continues across the Nares Strait with no apparent lateral offset and that the Wegener Fault does not exist ( Oakey & Chalmers, 2012; Oakey & Damaske, 2006; Oakey & Stephenson, 2008; Rasmussen & Dawes, 2011; see also further references and arguments in Gion *et al*., 2017). Despite overwhelming evidence against the Wegener Fault, field geologists continue to take its existence as a fact and use it to discuss local field observations and interpretations (e.g., Gilotti *et al*., 2018; von Gosen *et al*., 2019).

This calls for strengthened collaborations between geophysicists and field geologists. It also further highlights the importance of interdisciplinary studies when mapping and interpreting major faults. Interdisciplinary studies should include at least some regional (e.g., geophysical) datasets, which are becoming more broadly available and user-friendly, rather than exclusively local fieldwork data. It is necessary to establish a methodology for the classification of faults in order to clearly segregate beyond-reasonable-doubts faults observed directly on specific datasets, e.g., during fieldwork and/or on geophysical datasets (e.g., San Andreas fault – Crowell, 1979; Grant Ludwig *et al*., 2019; Huffman, 1972; Molnar & Atwater, 1973; – and Timanian thrusts systems in the Norwegian Barents Sea and Fram Strait – Klitzke *et al*., 2019; Koehl, 2020; Koehl *et al*., 2022a; Koehl *et al*., 2023a; Koehl & Mottram, 2024) from tentative faults (i.e., inferred and thus not directly observed on any specific dataset; e.g., Wegener Fault and De Geer Zone). For example, the latter may be called “lineaments” or “zones” rather than “faults”. In addition, it is necessary to clearly report the amount and nature of the uncertainty associated with (1) the interpretation of the involved datasets and (2) each individual fault. This especially includes data collected and observations made during fieldwork, whose interpretation is no less subjective than that of geophysical datasets.

The new model gives further weight to Orogenic Bridge Theory and the global correlation of all major (≥ a few tens km offset) transform faults with rift-orthogonal orogens on adjacent margins ( Koehl & Foulger, 2024). The theory suggests that all major transform faults initiate along preexisting, rift-oblique thrust systems, as suggested for the Molloy and Spitsbergen fault zones along Timanian thrust system ( Figure 9a–b and Figure 10a–b).

## Conclusions

Two several kilometers wide south- and NNE-dipping shear zones of probable late Neoproterozoic age, the Risen and Kinnhøgda–Daudbjørnpynten fault zones, extend past the presumed location of the De Geer Zone west of Spitsbergen. The shear-zone geometries and kinematics are consistent with a formation during the Timanian Orogeny. Both fault zones are continuous and do not show any trace of lateral offset. In addition, the fault segments of the presumed De Geer Zone west of Spitsbergen developed along inherited, moderately west-dipping Caledonian thrust systems and show exclusively normal kinematic indicators, minor Eurekan contractional reworking, and limited, tens of km extent inconsistent with hundreds of km transform movements. Thus, the De Geer Zone does not exist and the faults presumably associated with the De Geer Zone accommodated dominantly vertical movements. The present results therefore suggest major revisions to all current Phanerozoic paleogeographic reconstructions for Arctic regions.

The present study shows the importance of interdisciplinary approaches when trying to resolve large-scale tectonics and calls for caution with the extrapolation of local fieldwork data from deeply eroded Arctic regions to larger areas without supporting regional (e.g., geophysical) evidence. An important task for future studies is to distinguish directly observed faults from indirectly inferred structures by using a discrete nomenclature for the latter (e.g., “lineament” or else) and further encourage the discussion of the uncertainty associated to new and past interpretations.

## Ethics and consent

Ethical approval and consent were not required.

## Data Availability

Supplement rotation file for the presented plate reconstruction in Figure 11. The Two-Way Time seismic reflection data analyzed in the present contribution is from the DISKOS database (Norwegian National Data Repository for Petroleum Data) of the Norwegian Offshore Directorate and from the University of Bergen. Access to the data for research purposes can be obtained by contacting the Norwegian Offshore Directorate at https://www.npd.no/om-oss/kontakt-oss/ and Prof. Rolf Mjelde from the University of Bergen ( Rolf.Mjelde@uib.no). DataverseNO: Extended data for ‘The myth of the De Geer Zone: a change of paradigm for the opening of the Fram Strait’, https://doi.org/10.18710/J98MLA ( Koehl, 2025) This project contains the following extended data: ReadMe.txt. Replication_data_for_Koehl_2024.zip (high resolution versions of figures 1–11 included in this manuscript, in jpg format. All copyright permissions granted) Supplements_for_Koehl_2024.zip (high-resolution versions of the supplementary figure 1–3 in jpg format and rotation file for the presented plate reconstruction in Figure 11. All copyright permissions granted) Data are available under the terms of the Creative Commons Zero "No rights reserved" data waiver (CC0 1.0 Public domain dedication).
