# Supplementary material for: The myth of the De Geer Zone: a change of paradigm for the opening of the Fram Strait
Source: Open Res Eur. 2025 Mar 17;4:1. Originally published 2024 Jan 3. [Version 2] doi: 10.12688/openreseurope.16791.2 (PMC12033982; doi:10.12688/openreseurope.16791.2)
Supplement: Peer review history

# Reviewer report: Reviewer response for version 2

**DOI:** 10.21956/openreseurope.21354.r53116

**Published:** 2025-04-26

**Authors:** Filippo Carboni

**Affiliations:** 1 Albert-Ludwigs-University Freiburg, Freiburg, Germany

This interesting study aims at challenging the prevailing paradigm of the De Geer Zone, while supporting and suggesting alternative geological interpretations of Fram Strait and Arctic openings. The manuscript is of particular interest for regional geologists mainly focused on the study of these areas.

 I believe that the author well addressed the suggestions, critics and comments made during the first round of review, and that the geological reasoning and interpretations are supported by the data. However I would suggest further comments and possible edits.

 1.  “Structural fieldwork onshore western Spitsbergen has shown that the area is dominated by N–S to NNE–SSW-striking faults, which show indications of **up to 10 km dextral strike-slip displacement**.” 

 For a single fault or in total?

 2.  “The De Geer Zone is a major structural element of the west Spitsbergen transform margin that is believed to have accommodated 400 kilometers of dextral strike-slip movement during the opening of the **Northeast Atlantic and Arctic** **oceans and of the Fram Strait **in the Cenozoic”.

 I would suggest to modify in “… , the Arctic oceans and the Fram Strait…“

 3. Although the author well displayed the structures in Fig. 1, along with their easily understandable acronyms, I would suggest to add the acronyms of the different structures also in the text. At least the first time a name appears. This would make the link between text and figure easier.

 4. “…  NW–SE-striking brittle faults in the area, thus supporting the possible reactivation of the Senja Shear Belt as a **strike-slip** fault during the Cenozoic ( **e.g.,** Distelbrink, 2024).”

 Please add the sense of slip if possible. If not, this could be stated in the following sentence related to the lack of knowledge about the fault's kinematics

 Writing "recent studies" the reader would expect to have more than one reference, even if presented with e.g.,. Would it be possible to add further important  reference(s)?

 5. I would suggest a few edits of Figure 1

 Orient this figure horizontally instead of vertically. Such to have the two panels side by side.

 There is a refusal transparent map, which should be removed.

 It is not clear what is the difference between line 2 and S2 or 3 and S3...and so on. There is no correlation between the names of the lines in this figure and the profiles shown in Figs. 2, 3, 4.

 Please state the source for the bathymetric data. Is that the EBWBL 115m from EMODnet?

 6.  “… e.g., western Barents Sea–Svalbard margin and other transform margins worldwide, and for the interpretation of major paleo-transform faults, e.g., De Geer Zone and Wegener Fault.”

 I believe it is no completely clear what the author means. How this work would help in the interpretation of paleo-transform faults, especially if the Wegener Fault is cited, being very far from the AOI? Methodologically? This could be introduced slightly more.

 7.  “overall **top-SSW**”. I believe top-to-SSW would be better

 8. “ Moreover, movement along NW–SE-striking faults in Senja and Kvaløya is yet to be accurately constrained as, **as up to now**, no geochronological constraints **are available** for these faults.”

 I would modify the sentence in:  “Moreover, movement along NW–SE-striking faults in Senja and Kvaløya is yet to be accurately constrained as no geochronological constraints are currently available for these faults.”

 9.  I believe that the Vimsodden–Kosibapasset Shear Zone does not appear in any figure. Please add to Fig. 1 i.e.

 10. I would not refer to Figures 2, 3 and 4 already in the “Goals of the study” but I would suggest to keep the figures for the presentation of the results in the following parts of the manuscript.

 11. “ zones. The geometry and kinematics were then used to infer the possible timing of formation of the structures.”

 For inferring the fault timing the author should add the seismic stratigraphy as well

 12. The author should describe how he interpreted and defined these seismic artifacts (Fig. 2).

 13. It is not clear from where the time map shown in Fig. 6 come from. Did the author used other profiles covering the entire area of the time map?

 14. As previously commented by reviewer 1 (Comment 30) I would avoid the terms S-shaped and Z-shaped. I would keep using the term irregular of curved (curvy) reflections. Especially since Z and S have a particular meaning in structural geology, as parasitic folds; in addition I believe that such a specific description does not look really important in the description of the profiles.

 15. I would suggest to show these multiple artifacts in the profile (Fig. 7)

 16. When describing the location of the profiles and features within them, I would suggest to avoid stating the locations, but giving the name of the line (number) as shown in Fig. 1b. I believe there is a major lack of link between the seismic profiles, the overall text and Fig. 1b.

 17. I would suggest to add the toponyms cited in the text during the description of the profiles, in the figures and use the name of the seismic line in the text.

 18. “The newly mapped Timanian thrust systems north and west of Svalbard ( **Figure 1b**, Figure 2, Figure 3, Figure 4, Figure 7) probably…”

 I would suggest to use "previous" tectonic interpretation map(s) and keep you novel interpretation for i.e., Fig. 6

 19. In Figure 19 the author could add specific time range per each panel

Is the study design appropriate and does the work have academic merit?

Yes

Is the work clearly and accurately presented and does it cite the current literature?

Partly

If applicable, is the statistical analysis and its interpretation appropriate?

Not applicable

Are all the source data underlying the results available to ensure full reproducibility?

No source data required

Are the conclusions drawn adequately supported by the results?

Yes

Are sufficient details of methods and analysis provided to allow replication by others?

Yes

Reviewer Expertise:

NA

I confirm that I have read this submission and believe that I have an appropriate level of expertise to confirm that it is of an acceptable scientific standard, however I have significant reservations, as outlined above.

# Reviewer report: Reviewer response for version 2

**DOI:** 10.21956/openreseurope.21354.r52234

**Published:** 2025-04-02

**Authors:** Tuncay Taymaz

**Affiliations:** 1 Istanbul Technical University, Maslak, Turkey

This is an interesting and well-written study challenges the prevailing paradigm regarding the De Geer Zone near Spitsbergen. The study includes high quality illustrations that streamline the author's message right. The authors addressed nicely the earlier revisions and comments. However, CONCLUSIONS could be better styled and succinctly referring to the firm results obtained in the current study.  Hence, I have no more further remarks to append as I am happy as it is at the current revision. I recommend accept.

Is the study design appropriate and does the work have academic merit?

Yes

Is the work clearly and accurately presented and does it cite the current literature?

Yes

If applicable, is the statistical analysis and its interpretation appropriate?

Yes

Are all the source data underlying the results available to ensure full reproducibility?

Yes

Are the conclusions drawn adequately supported by the results?

Yes

Are sufficient details of methods and analysis provided to allow replication by others?

Yes

Reviewer Expertise:

Active Tectonics, Geophysics, Seismology, Seismotectonics, Geodynamics, Marine Geophysics, Seismic Tomography, Tsunamis

I confirm that I have read this submission and believe that I have an appropriate level of expertise to confirm that it is of an acceptable scientific standard.

# Reviewer report: Reviewer response for version 1

**DOI:** 10.21956/openreseurope.18142.r45796

**Published:** 2024-11-26

**Authors:** Peter D Clift

**Affiliations:** 1 University College London, London, England, UK

This is an interesting and at least regionally provocative study of the Svalbard offshore that should be important to those working on the tectonics of the North Atlantic and Arctic Ocean. The study examines two transects offshore Svalbard that cut across the strike of two major structures known as the De Geer Zone and which is widely considered as the transform continental margin related to the opening of the oceanic basin and the southeast displacement of Svalbard relative to Greenland. The paper does a pretty convincing job of showing that earlier structures dating from a Precambrian origin essentially not set across the linear and that therefore it cannot be a significant transform structured during the Cenozoic. I provide a number of specific comments below here about my primary concern is that although the old model is shown to be inconsistent with the cross structure? The author doesn’t do a very good job of proposing a new revised model. For example, the Malloy Fracture Zone which offsets the Mid Atlantic Ridge substantially west of the island would be expected to align with a transform continental margin. If that is not the De Geer Zone then I think it’s only reasonable to ask where the transform margin is located. I think that a model figure showing the preferred evolution of the margin should be provided and at the very least we need to see where the continent ocean boundary is on the figure one map so that we can understand the relationship between the structures and the old continental crust underlying small bar and the structures in the oceanic lithosphere. At the moment the reader has left perplexed as to what the author believes regarding the opening history of the North Atlantic beyond the fact that he doesn’t like the existing preferred models

 Comments on Introduction

 “apart from a few evidence of” – should be “apart from a few pieces of evidence for”

 “which extend c. 80–90 km west of the coastline of Spitsbergen”  - and is that West or east of the De Geer Zone?

 e.g., Wegener Fault  - Where is that?

 Timanian thrusts systems -  I don’t know what Timanian means or how old that is. Please at least add ages here.

 Comments on Geological setting

 Kinnhøgda–Daudbjørnpynten fault zone – Should capitalize proper names - Kinnhøgda–Daudbjørnpynten Fault Zone

 Caledonian contraction - contraction is an odd phrase. Compression?

 Figure 1 – What do the white numbers on the seismic lines refer to? Put the continent-ocean boundary on the figure.

 during the Caledonian Orogeny – Which was when?

 Late Devonian Svalbardian Orogeny – Provide age in Ma too

 gradually sled past one another -  gradually slid past one another

 Comments on Results

 The package north of Spitsbergen -  I am not sure what you’re talking about. Can you label this better on Figure 2? Likewise “The package west of Nordenskiöld Land”. Do you mean the thrust sheet?

 Labels on Figures 2-5 are too small

 Figure 3 – I’m rather sceptical of your interpretation of the shallow is pink reflector which seems to dive down rather deeply towards the left side of the interpreted section. It looks to me like it could be running much shallow and therefore avoiding the rather steep inclination on the left side of the profile.

 Figure 5 – I only see a and c not b and d. This means I’m unable to judge whether your interpretation is appropriate or not.

 ca. 2.5 to 5.0 seconds TWT  - you were abbreviating “approximate” as “c.” not “ca.” Earlier in the manuscript. Do you need to be consistent.

 Nordenskiöld Land ( Figure 6) - Nordenskiöld Land is not labelled on this figure.

 Both packages also shows - Both packages also show

 Comments on Interpretation

 Precambrian–early Paleozoic metasedimentary rocks -  Lower Paleozoic. Early is for time not stratigraphy

 “northward-leaning”   - “northward-verging” or ““northward-dipping”?

 down-south  - down to the south

 On the contrary  - In contrast

 The package north of Spitsbergen is hereby named the Risen fault zone – The whole package is the fault zone?  Capitalize the proper name. Likewise, Kinnhøgda–Daudbjørnpynten fault zone

 event of contraction  - event implies a short duration which you don’t know. “compressional episode” would be better

 pinching out reflection packages -  reflection packages with pinching out geometries

 salt diapir – How about a shale diapir?

 Comments on Discussion

 verging folds and top-east thrusts (e.g., Bergh & Grogan, 2003; Birkenmajer, 1975; Birkenmajer, 2004; Dallmann et al., 1993; Dumais & Brönner, 2020; Gee et al., 1994; Hjelle, 1979; Johansson et al., 2004; Johansson et al., 2005; Lyberis & Manby, 1999; Lyberis & Manby, 2001; Maher, 1988; Maher et al., 1986; Maher et al., 1989; Maher et al., 1997; Manby, 1986; Manby & Lyberis, 2001a; Manby & Lyberis, 2001b; Tessensohn et al., 2001a; Tessensohn et al., 2001b; Tessensohn et al., 2001c; Welbon & Maher, 1992; Witt-Nilsson et al., 1998; von Gosen & Piepjohn, 2001) -  This is an excessive number of citations for a single point.

 NNE-dipping geometry -  Oceanic fracture zones are generally vertical and are in any case formed within new lithosphere. I don’t see how the dip of the earlier structure in the older continental crust would influence the transform faults in the new crust.

 post-Caledonian collapse  -  In what direction?

 Comments on “Implications for the De Geer Zone and plate tectonics reconstructions”

 N–S-striking Cenozoic paleo-transform fault -  surely the transform should be NW-SE trending like the Molloy Fracture Zone.

 approximately three times more than the San Andreas fault in California – This is not a strong argument because it’s a different setting and plate motion rates could be very different. Also the San Andreas fault is not a transform passive margin but an intra-continental transform.

 half of the distance Svalbard moved away from Greenland in the Cenozoic (c. 200 km) was accommodated by lateral movements along the two, c. 200 km long, NW–SE-striking transform faults in the Fram Strait, the Molloy and Spitsbergen fracture zones -  OK but your map Figure does not show how these offset the rifted margin. Where are the transform passive margins in your revised model?

 continent–ocean boundary in the Fram Strait is located at least 80–90 km to the west of Spitsbergen  - Ok then show that.

 Wegener Fault, a thousand of kilometer-long sinistral strike-slip fault inferred between Ellesmere Island and northwestern Greenland -  Need to show this on a map. Also Nares Strait

Is the study design appropriate and does the work have academic merit?

Yes

Is the work clearly and accurately presented and does it cite the current literature?

Yes

If applicable, is the statistical analysis and its interpretation appropriate?

Not applicable

Are all the source data underlying the results available to ensure full reproducibility?

Partly

Are the conclusions drawn adequately supported by the results?

No

Are sufficient details of methods and analysis provided to allow replication by others?

Yes

Reviewer Expertise:

NA

I confirm that I have read this submission and believe that I have an appropriate level of expertise to state that I do not consider it to be of an acceptable scientific standard, for reasons outlined above.

# Response

**Published:** 2025-02-18

**Authors:** Jean-Baptiste Koehl

**Affiliations:** Geosciences, Universitetet i Oslo, Oslo, Oslo, Norway

Dear Prof. Clift, thank you very much for your input on the manuscript, it is highly appreciated. Here is our reply to your comments. We hope the changes we implemented improve the shortcomings of the manuscript highlighted by your comments and suggestions. Please do not hesitate to contact us shall this not be the case for some comments.  

**Comments by the reviewer**

**Comment 1:** This is an interesting and at least regionally provocative study of the Svalbard offshore that should be important to those working on the tectonics of the North Atlantic and Arctic Ocean. The study examines two transects offshore Svalbard that cut across the strike of two major structures known as the De Geer Zone and which is widely considered as the transform continental margin related to the opening of the oceanic basin and the southeast displacement of Svalbard relative to Greenland. The paper does a pretty convincing job of showing that earlier structures dating from a Precambrian origin essentially not set across the linear and that therefore it cannot be a significant transform structured during the Cenozoic. **Response:** Agreed.

**Changes:** None required.  

**Comment 2:** I provide a number of specific comments below here about my primary concern is that although the old model is shown to be inconsistent with the cross structure? The author doesn’t do a very good job of proposing a new revised model. For example, the Malloy Fracture Zone which offsets the Mid Atlantic Ridge substantially west of the island would be expected to align with a transform continental margin. If that is not the De Geer Zone then I think it’s only reasonable to ask where the transform margin is located. I think that a model figure showing the preferred evolution of the margin should be provided and at the very least we need to see where the continent ocean boundary is on the figure one map so that we can understand the relationship between the structures and the old continental crust underlying small bar and the structures in the oceanic lithosphere. At the moment the reader has left perplexed as to what the author believes regarding the opening history of the North Atlantic beyond the fact that he doesn’t like the existing preferred models

**Response:** Agreed. The currently active NW–SE-striking Molloy and Spitsbergen fault zones align with NW–SE- to WNW–ESE-striking Timanian thrust systems, which were reactivated as sinistral strike-slip/transform faults in the mid–late Cenozoic (see new U–Pb ages for synkinematic carbonates from Koehl and Mottram, 2024).

**Changes:** Added a new Figure 9 to show the schematics of the proposed model, which involves local dextral strike-slip movements along N–S-striking faults (i.e., parallel to the presumed De Geer Zone) in the order of a few km, and tens of km sinistral movements along inherited Timanian thrust systems. In addition, added a few paragraphs to the second section of the “Discussion” chapter to further detail the proposed evolution of the transform margin: “The newly mapped Timanian thrust systems north and west of Svalbard ( Figure 1b, Figure 2, Figure 3, Figure 4, Figure 7) probably controlled the formation of currently active NW–SE-striking transform faults in the Fram Strait, the Spitsbergen and Molloy fault zones ( Figure 1a–b; Koehl *et al.*, 2021; Koehl & Mottram, 2024). A new tectonic model in which Timanian thrust systems accommodate most of the transform is proposed ( Figure 9a–b). As a result of sinistral movements along WNW–ESE-striking Timanian faults, N–S-striking Caledonian and Eurekan are reactivated as minor dextral strike-slip faults ( Figure 9b). This model is supported by the repeated reactivation of Timanian thrust systems as sinistral strike-slip faults in the Phanerozoic (e.g., Koehl, 2020; Koehl *et al.*, 2022a; Koehl *et al.*, 2023a; Mazur *et al.*, 2009; von Gosen & Piepjohn, 2001; Ziemniak *et al.*, 2022) and by indications of dextral movements along N–S- to NNE–SSW-striking faults onshore Spitsbergen (e.g., Bergh *et al.*, 1997; Harland & Horsfield, 1974; Maher *et al.*, 1997) and Prins Karls Forland (e.g., Lepvrier, 1990).

**Figure 9. Schematic model of the interaction of WNW–ESE-striking Timanian faults and N–S-striking Caledonian and Eurekan faults in Svalbard during the opening of the Fram Strait.** ( a) Inherited, late Neoproterozoic Timanian thrust systems such as the Kinnhøgda–Daudbjørnpynten and Kongsfjorden–Cowanodden fault zones (KDFZ and KCFZ; red lines) are reactivated as sinistral strike-slip faults during oblique extension and localize the formation of major transform faults, the Spitsbergen and Molloy fault zones (SPZ and MFZ; dashed yellow lines). ( b) Preexisting NNE–SSW-striking (e.g., Caledonian and Eurekan) structural grain and fabrics are reactivated as local dextral strike-slip faults (orange lines) to accommodate tectonic adjustments during NW–SE-striking transform faulting. The green line shows the amount of dextral movement along N–S-striking faults, i.e., a few km up to 10 km, and the blue line sinistral movement along WNW–ESE-striking faults, which is in the order of a few tens of km. Notice how this tectonic setting may give the illusion that the main transform fault strikes N–S instead of NW–SE due to the relatively larger number of N–S-dextral faults in the area. This impression is reinforced if the main few NW–SE-striking faults do not crop out or if related outcrops are of poor quality. Abbreviations: IYF: Isfjorden–Ymerbukta Fault; KCFZ: Kongsfjorden–Cowanodden fault zone; KDFZ: Kinnhøgda–Daudbjørnpynten fault zone; MFZ: Molloy fault zone; SEDL: Svartfjella–Eidembukta–Daudmannsodden Lineament; SFZ: Spitsbergen fault zone. Extrapolating the schematic fault-block model from Figure 9 to Spitsbergen, the maximum amount of lateral movement along N–S-striking faults is in the order of 10 km. This is not cumulative because, in this model, N–S-striking faults are simply accommodating local tectonic adjustments in between major WNW–ESE-striking discontinuities in the crust (Timanian thrust systems) and are segmented (e.g., Hornsund Fault Complex west of Spitsbergen, which dies out north of Prins Karls Forland and south of the Risen fault zone; Figure 1b). Since the blocks in Figure 9 are schematic and rocks deform more complexly, i.e., partitioning deformation and distributing displacement along more structures, this is an upper estimate and dextral offsets of a few km are more likely. This is in agreement with estimates from previous studies along N–S-striking dextral faults in western Spitsbergen, e.g., N–S-striking Svartfjella–Eidembukta–Daudmannsodden Lineament in Oscar II Land ( Maher *et al.*, 1997; Figure 1b) and NNE–SSW-striking Isfjorden–Ymerbukta Fault ( Bergh *et al.*, 1997; Braathen *et al.*, 1999; Harland & Horsfield, 1974). This model does not require hundreds km dextral transform movements between Greenland and Svalbard. It does not require a sudden (and thus far unexplained) change in plate kinematics at breakup at ca. 24 Ma either, when the NW–SE-striking Molloy and Spitsbergen fault zones are supposed to have taken over transform motions for the N–S-striking De Geer Zone (e.g., Faleide *et al.*, 2008; Doré *et al.*, 2015). Restoring Greenland and Svalbard prior to the opening of the Fram Strait would thus come down to closing the oceanic crustal domain at the Knipovich Ridge (and possibly at the Molloy Ridge) along the two NW–SE-striking transform faults, the Spitsbergen and Molloy fault zones ( Figure 1a). Since new high-resolution magnetic data have shown that the oceanic crustal domain between Greenland and Svalbard is relatively narrow (c. 100–200 km; Dumais *et al.*, 2020; see ocean–continent boundary in Figure 1a), the first step would be relatively simple and involves matching the northern edge of the Kinnhøgda–Daudbjørnpynten fault zone shows in Figure 1b and Figure 4 with its counterpart at the Hovgård Ridge, where a comparable Timanian thrust was mapped on seismic reflection data ( Koehl, 2020; Figure 1a). To proceed with closing the Arctic Ocean and northeastern Atlantic Ocean, one would need to restore the Timanian Orogen in the Fram Strait to its original crustal thickness (> 40 km), while closing the remaining oceanic crustal domains at the Gakkel and Mohns ridges. As a result of the proposed restoration, the Svalbard Archipelago would have lain c. 200 km closer to Greenland prior to the opening of the Fram Strait, i.e., east rather than north of Greenland as suggested by previous correlations ( Harland, 1967; Harland, 1969; Jones *et al.*, 2016; Jones *et al.*, 2017; Majka *et al.*, 2021; Piepjohn *et al.*, 2016). This configuration is likely inherited from the Timanian Orogen and has, thus, likely persisted from the end of the Timanian Orogeny in the latest Neoproterozoic until mid–late Cenozoic extension. Jones *et al.*, (2016) have argued that the occurrence of thin volcanic ash layers probably erupted in Kapp Washington in northern Greenland and Ellesmere Island in Arctic Canada ( Figure 1a) in lower Cenozoic strata in central Spitsbergen suggested close proximity of Svalbard with these two volcanic centers. However, volcanic ash may travel over large distances (> 2000 km) and a single ash bed may cover broad areas. For example, the Lava Creek ash bed was erupted from Yellowstone in the Pleistocene and is found all over Texas and western Louisiana, i.e., up to 2300 km from the volcanic center ( Izett & Wilcox, 1982). This distance is larger than that between the volcanic centers in Ellesmere Island and northern Greenland and the ash layers in central Spitsbergen at present (respectively 1900 and 1200 km). In the present model, Svalbard is interpreted to have been located some 200 km closer to both volcanic centers in the early Cenozoic, thus further reducing the distance of Svalbard with the volcanic centers. The proposed model is therefore in agreement with an origin in northern Greenland and Ellesmere Island for the ash layers in central Spitsbergen.”. Finally, added a new Figure 10 showing the tectonic development of the transform margin.  

**Comment 3:** Comments on Introduction “apart from a few evidence of” – should be “apart from a few pieces of evidence for”

**Response:** Agreed.

**Changes:** Corrected accordingly.  

**Comment 4:** “which extend c. 80–90 km west of the coastline of Spitsbergen”  - and is that West or east of the De Geer Zone?

**Response:** This is west of the presumed location of the De Geer Zone. The author of the present manuscript concedes that this should be specified.

**Changes:** Added “, i.e., west of the presumed location of the De Geer Zone” to the targeted sentence.  

**Comment 5:** e.g., Wegener Fault  - Where is that?

**Response:** Agreed.

**Changes:** Added a new Figure 1a  

**Comment 6:** Timanian thrusts systems -  I don’t know what Timanian means or how old that is. Please at least add ages here.

**Response:** Agreed.

**Changes:** Added “late Neoproterozoic” to the abstract.  

**Comment 7:** Comments on Geological setting: Kinnhøgda–Daudbjørnpynten fault zone – Should capitalize proper names - Kinnhøgda–Daudbjørnpynten Fault Zone

**Response:** Disagreed. See response to comment 24.

**Changes:** See response to comment 24.  

**Comment 8:** Caledonian contraction - contraction is an odd phrase. Compression?

**Response:** “Compression” involves a volume reduction, whereas “contraction” simply means to become smaller. Since tectonic stresses do not necessarily involve volume reduction, it is best to use “contraction”.

**Changes:** None.  

**Comment 9:** Figure 1 – What do the white numbers on the seismic lines refer to? Put the continent-ocean boundary on the figure.

**Response:** Agreed with the comment on the continent–ocean boundary. The white numbers at the end of the white lines symbolizing the location of the seismic sections displayed in the manuscript correspond to the figure number in which the respective seismic lines are shown. **Changes:** Added the continent–ocean boundary in Figure 1b and in figure legend.  

**Comment 10:** during the Caledonian Orogeny – Which was when?

**Response:** Agreed.

**Changes:** Added “at ca. 465–425 Ma” and reference to Horsfield (1972), Dallmeyer et al. (1990a), and Faehnrich et al. (2020) in the targeted sentence.  

**Comment 11:** Late Devonian Svalbardian Orogeny – Provide age in Ma too

**Response:** Agreed.

**Changes:** Added “(potentially 383–365 Ma)”.  

**Comment 12:** gradually sled past one another -  gradually slid past one another

**Response:** Agreed.

**Changes:** Corrected “sled” into “slid”.  

**Comment 13:** Comments on Results: The package north of Spitsbergen -  I am not sure what you’re talking about. Can you label this better on Figure 2? Likewise “The package west of Nordenskiöld Land”. Do you mean the thrust sheet?

**Response:** Yes, this is what is meant but cannot be said yet because still in the “Description” sub-section.

**Changes:** None.  

**Comment 14:** Labels on Figures 2-5 are too small

**Response:** The readers are recommended to download the figures in their full resolution from Dataverse NO (see Extended Data section), which is necessary to identify the targeted structures (e.g., asymmetric folds) throughout the presented seismic sections. Throughout the manuscript, the reader is referred 8 times to the relevant Open Access dataset (Koehl, 2023). **Changes:** None.  

**Comment 15:** Figure 3 – I’m rather sceptical of your interpretation of the shallow is pink reflector which seems to dive down rather deeply towards the left side of the interpreted section. It looks to me like it could be running much shallow and therefore avoiding the rather steep inclination on the left side of the profile.

**Response:** The reviewer is welcome to disagree with the presented interpretation and to present his own interpretation. As mentioned in the response to comment 14, the seismic sections presented are available Open Access at full resolution on DataverseNO (Koehl, 2023). The defining factor in the presented interpretation is the gently west-dipping reflection (see white arrows in figure 3b - link attached below) just east of the shallow basement high. This gently west-dipping reflection looks genuine and would then represent the top of Precambrian–lower Paleozoic basement rocks, thus the steep slope.

Figure 3b: https://s3-eu-west-1.amazonaws.com/openreseurope/linked/247277.Figure_3B-_See_white_arrows_in_figure_below.png 

**Changes:** None.  

**Comment 16:** Figure 5 – I only see a and c not b and d. This means I’m unable to judge whether your interpretation is appropriate or not.

**Response:** (b) is the uninterpreted version of (a) and is found just below (a). The same for (d) with (c). The same logic is applied for Figures 2–5, in which the interpreted section is located above the uninterpreted version. Nevertheless, the author of the present manuscript concedes that the separation between each figure part could be better outlined.

**Changes:** Thickened the separating lines between the 4 figure parts.  

**Comment 17:** ca. 2.5 to 5.0 seconds TWT  - you were abbreviating “approximate” as “c.” not “ca.” Earlier in the manuscript. Do you need to be consistent.

**Response:** Disagreed. “ca.” applies exclusively to time, whereas “c.” does not.

**Changes:** None.  

**Comment 18:** Nordenskiöld Land (Figure 6) - Nordenskiöld Land is not labelled on this figure. **Response:** Agreed.

**Changes:** Added Nordenskiöld Land to the figure and figure caption.  

**Comment 19:** Both packages also shows - Both packages also show

**Response:** Agreed.

**Changes:** Corrected as suggested.  

**Comment 20:** Comments on Interpretation: Precambrian–early Paleozoic metasedimentary rocks -  Lower Paleozoic. Early is for time not stratigraphy

**Response:** Agreed.

**Changes:** Corrected this issue throughout the text.  

**Comment 21:** “northward-leaning”   - “northward-verging” or ““northward-dipping”?

**Response:** Disagreed. “leaning” is a descriptive term, while “dipping” and “verging” are interpretative because they imply an interpretation as, e.g., faults or folds.

**Changes:** None.  

**Comment 22:** down-south  - down to the south

**Response:** Both versions are accepted, the latter being shorter and easier to read.

**Changes:** None.  

**Comment 23:** On the contrary  - In contrast

**Response:** Agreed.

**Changes:** Corrected “on the contrary” into “in contrast”.  

**Comment 24:** The package north of Spitsbergen is hereby named the Risen fault zone – The whole package is the fault zone?  Capitalize the proper name. Likewise, Kinnhøgda–Daudbjørnpynten fault zone

**Response:** Yes, the whole package. The author of the present manuscript was cautioned by other researchers in Norway to capitalize new structure names. North American geologists have cautioned in capitalizing the name of specific structures because they do not represent a specific place (e.g., San Andreas fault; “San Andreas” is capitalized because it is an actual place, but “fault” is not because it is the name of a structure, which extends over many places). The author of the present manuscript is open to adjusting to a standardized nomenclature should there be one broadly accepted among the Geoscience community. **Changes:** None yet.  

**Comment 25:** event of contraction  - event implies a short duration which you don’t know. “compressional episode” would be better

**Response:** Agreed.

**Changes:** Corrected “event” into “episode” where relevant throughout the manuscript.  

**Comment 26:** pinching out reflection packages -  reflection packages with pinching out geometries

**Response:** Agreed.

**Changes:** Rephrased into “pinching-out reflection packages”.  

**Comment 27:** salt diapir – How about a shale diapir?

**Response:** Agreed.

**Changes:** Added “or shale” and reference to van Rensbergen et al. (1999) to the targeted sentence.  

**Comment 28:** Comments on Discussion verging folds and top-east thrusts (e.g., Bergh & Grogan, 2003; Birkenmajer, 1975; Birkenmajer, 2004; Dallmann et al., 1993; Dumais & Brönner, 2020; Gee et al., 1994; Hjelle, 1979; Johansson et al., 2004; Johansson et al., 2005; Lyberis & Manby, 1999; Lyberis & Manby, 2001; Maher, 1988;  Maher et al., 1986; Maher et al., 1989; Maher et al., 1997; Manby, 1986; Manby & Lyberis, 2001a;  Manby & Lyberis, 2001b; Tessensohn et al., 2001a; Tessensohn et al., 2001b; Tessensohn et al., 2001c; Welbon & Maher, 1992; Witt-Nilsson et al., 1998; von Gosen & Piepjohn, 2001) -  This is an excessive number of citations for a single point.

**Response:** Agreed. It, however, demonstrates the knowledge of the author of the present manuscript of existing studies in the study area and gives credit to as many as possible. Should each author of the above-mentioned studies be asked, which studies does the reviewer think these authors would suggest should remain in the present sentence? Most of these authors would most likely choose dominantly their own studies. Thus, it is best to refer to them all once and for all so no one feels segregated against.

**Changes:** None.  

**Comment 29:** NNE-dipping geometry -  Oceanic fracture zones are generally vertical and are in any case formed within new lithosphere. I don’t see how the dip of the earlier structure in the older continental crust would influence the transform faults in the new crust.

**Response:** Disagreed. The Molloy transform fault zone is steep but still dips to the north-northeast (e.g., Koehl, 2020; Koehl et al., 2021). Since the two plates were adjacent prior to the breakup, and since the two actual transform faults, the Molloy and Spitsbergen transform fault zones, are parallel to crustal-scale Timanian thrust systems, it is argued that the latter controlled the formation of these two transform faults. The reviewer is referred to an ongoing discussion and associated manuscript on Orogenic Bridge Theory, which presents a new global correlation between all major (> tens of km offset) transform faults on Earth with orogens striking perpendicular or highly oblique to the active rift on adjacent margins (Koehl and Foulger, 2024). In the case of the northeastern Atlantic and Arctic oceans, in which breakup occurred coevally at ca. 56 Ma, the Timanian Orogen represented a major oblique barrier, which hindered rifting and breakup and forced the rift to step and bypass the orogen. Thus, breakup in the Fram Strait occurred much later, at ca. 24 Ma. This is because the inherited rift-oblique (WNW–ESE-striking) Timanian structures were not suitably oriented to thin the crust efficiently and it thus took longer to thin the crust in the Fram Strait down to breakup. Furthermore, as suggested in the response to comment 32, the transform faults likely initiated during rifting in continental crust and likely used preexisting discontinuities, e.g., Timanian thrust systems, to initiate lateral movements between the two plates prior to continental breakup. This is supported by brand-new U–Pb ages of synkinematic carbonates along sinistral strike-slip segments of the Kongsfjorden–Cowanodden fault zone in western Spitsbergen (Koehl and Mottram, 2024). The contrary, i.e., that Timanian thrust systems did not reactivate as strike-slip (or continental transform faults) prior to breakup and that the NW–SE-striking Molloy and Spitsbergen transform fault zones did not reactivate/overprint Timanian thrusts, e.g., the Kinnhøgda–Daudbjørnpynten and Kongsfjorden–Cowanodden fault zones (Figure 1a–b), is not convincing given the strong similarities in strike, dip, and location of these two transform faults with inherited thrusts.

**Changes:** Added “A similar controlling relationship was recently proposed for the Timanian Kongsfjorden–Cowanodden fault zone and the Spitsbergen fault zone based on new earliest Oligocene U–Pb ages for syn-tectonic carbonate cement along strike-slip fault segments of the former in west Spitsbergen ( Koehl & Mottram, 2024; Figure 1a–b).” to the second paragraph of the “Discussion” chapter.  

**Comment 30:** post-Caledonian collapse  -  In what direction?

**Response:** If an orogen, e.g., striking N–S, collapses, it would do so preferentially along an E–W-trending axis. However, this undermines the impact of inherited (i.e., pre-orogenic) oblique structural trends. In Svalbard for example, the Timanian Orogeny developed WNW–ESE-striking thrust systems during NNE–SSW-oriented contraction. Subsequent E–W-oriented Caledonian contraction reactivated WNW–ESE-striking Timanian thrust systems as reverse-sinistral faults (Koehl et al., 2022a) and formed new N–S-striking thrusts. Thus, when the orogen collapsed, it did so along both inherited WNW–ESE-striking Timanian and N–S-striking Caledonian faults, as shown by the parallelepiped shape of the Devonian Graben in northern Spitsbergen (e.g., Friend and Moody-Stuart, 1972; Dallmann and Piepjohn, 2020), which is probably bounded by N–S- and WNW–ESE-striking faults. The author of the present manuscript concedes that this should be better stated.

**Changes:** Added “and inverted Timanian and Caledonian thrusts in northern Spitsbergen (Koehl *et al.*, 2022a; Koehl *et al.*, 2023b)” to the first sentence of the “Devonian–Carboniferous extension” sub-section in the “Geological setting” chapter.  

**Comment 31:** Comments on “Implications for the De Geer Zone and plate tectonics reconstructions”: N–S-striking Cenozoic paleo-transform fault -  surely the transform should be NW-SE trending like the Molloy Fracture Zone.

**Response:** Agreed, it should be, but the De Geer Zone strikes N–S, which is yet another major inconsistency, which did not bother anyone before now. This suggests that the De Geer Zone model was already deeply anchored in the community when the actual transform faults, the Molloy and Spitsbergen fault zones, were discovered. The De Geer Zone was proposed at the beginning of the 20 ^th^ Century (e.g., De Geer, 1926; Holtedahl, 1936), whereas the Molloy and Spitsbergen fault zones were discovered in the second half of the 20 ^th^ Century (Johnson and Eckhoff, 1966; Crane et al., 1982). The author of the present manuscript concedes that this should be better highlighted in the introduction and discussion.

**Changes:** Added “The De Geer Zone strikes N–S and is thus oblique to the currently active NW–SE-striking Molloy and Spitsbergen fault zones ( Figure 1a), which implies a major change in plate kinematics at breakup (ca. 24 Ma; Engen *et al*., 2008). This change in spreading and plate movement direction is not explained in studies targeting the evolution of the Svalbard–Greenland transform margin (e.g., Faleide *et al.*, 2008; Doré *et al.*, 2015; Nemcok *et al.*, 2016), and its origin remains a mystery should such a change have occurred.” to the “Introduction” chapter at the beginning of the new “Cracks in the paradigm” sub-section. Also added “It does not require a sudden (and thus far unexplained) change in plate kinematics at breakup at ca. 24 Ma either, when the NW–SE-striking Molloy and Spitsbergen fault zones are supposed to have taken over transform motions for the N–S-striking De Geer Zone (e.g., Faleide *et al.*, 2008; Doré *et al.*, 2015).” and “This also applies to the De Geer Zone, which was largely supported by local structural field data onshore Spitsbergen (e.g., Bergh *et al.*, 1997; Braathen *et al.*, 1999; Harland & Horsfield, 1974; Maher *et al.*, 1997), but not by regional seismic studies (Austegard *et al*., 1988; Eiken, 1994; Riis & Vollset, 1988).” to the “Implications for the De Geer Zone and plate tectonic reconstructions” section of the “Discussion” chapter.  

**Comment 32:** approximately three times more than the San Andreas fault in California – This is not a strong argument because it’s a different setting and plate motion rates could be very different. Also the San Andreas fault is not a transform passive margin but an intra-continental transform.

**Response:** Agreed.

**Changes:** Deleted whole paragraph.  

**Comment 33:** half of the distance Svalbard moved away from Greenland in the Cenozoic (c. 200 km) was accommodated by lateral movements along the two, c. 200 km long, NW–SE-striking transform faults in the Fram Strait, the Molloy and Spitsbergen fracture zones -  OK but your map Figure does not show how these offset the rifted margin. Where are the transform passive margins in your revised model?

**Response:** The transform margin is located off the island of Bjørnøya to northern Svalbard and us characterized by the Molloy and Spitsbergen fault zones.

**Changes:** Added a new Figure 1a.  

**Comment 34:** continent–ocean boundary in the Fram Strait is located at least 80–90 km to the west of Spitsbergen  - Ok then show that.

**Response:** Agreed.

**Changes:** Added a new Figure 1a.  

**Comment 35**: Wegener Fault, a thousand of kilometer-long sinistral strike-slip fault inferred between Ellesmere Island and northwestern Greenland -  Need to show this on a map. Also Nares Strait.

**Response:** Agreed.

**Changes:** Added a new Figure 1a.

# Reviewer report: Reviewer response for version 1

**DOI:** 10.21956/openreseurope.18142.r39959

**Published:** 2024-05-21

**Authors:** Uri Schattner

**Affiliations:** 1 School of Environmental Sciences, University of Haifa, Haifa, Israel

This interesting and well-written study challenges the prevailing paradigm regarding the De Geer Zone near Spitsbergen. The study includes nice illustrations that convey the author's message. The text and illustrations are addressed in the comments below and the annotated manuscript. In general, when the study challenges a widely accepted paradigm, it should first present that paradigm. Then the "cracks" in the paradigm should be presented and lead to the main questions that gave motivation to the present study. This should lead to the aim of the study at the end of the introduction. The introduction should also include the general tectonic map of the entire area, with the study area within it, otherwise, the global reader will not be able to evaluate the study. All the geographic and tectonic features mentioned in the text should appear in that figure. Presenting the prevailing paradigm in the text and figure is important for the author in order to convince the reader that the paradigm should change. In this context, it is also important to add two additional figures – (1) a table with the commonly accepted geological history of the region (possibly separated into subregions). This table will also include a column with the new approach presented in the current transcript. (2) the second figure needed should include a sketch of the geological development of the region in 2-4 panels. This figure should appear in the discussion to support the text and show how the new interpretation fits into the regional tectonics.

 Below are some comments in addition to the annotated manuscript.

 Figure 1 should present a more general location map with the tectonic features, for the global reader.

 The introduction should end with a paragraph or two summarizing the previous paradigm, the open questions regarding that paradigm which are the motivation for present study, the aim of the present study. Part of this appears in the second and third paragraphs of the introduction and should be moved to the and of the introduction.

 Since this paper attempts to change the prevailing paradigm regarding a major fault zone, the author may consider presenting a figure that will show the previous interpretation of faults on a map and a seismic profile used in previous studies showing the De Geer Zone. Without these, the global reader who sees these structures for the first time will have difficulties in evaluating the new perspective of the present study.

 The current version jumps immediately to the author's interpretations.

 In Figure 2 - the names of the seismic profiles are very long. Please shorten the names to simple numbers and maybe add a conversion table. For example profile 1 will be MPD-MOFF-97….

 What does the vertical black line on the profile mean? Does the figure show a competent profile? This should be mentioned in corruption as well as in the figure.

 The "bolded bedding surfaces" are not convincing. Most, if not all, seem to be artifacts of wiggly reflectors common in those depths.

 In order to support the author's interpretation, it would be useful to mark names on the interpreted fault systems.

 It is not clear what the dashed white lines represent.

 In general, the red and black markings of faults seem like synthetic and antithetic faults bounding a graben that was subsequently inverted, as evident by the compression structures on the black faults.

 Figure 3 - same comment about the wiggly yellow lines.

 The undulating red horizons are presented as "Mylonitic shear surfaces". These horizons seem more like large folds.

 Please see the suggested additions to the deputation of the profiles in figures 2 and three.

 Figure 5 - Please separate the interpreted from the uninterpreted profiles

 In panel (a), the faults are marked along reflectors that do not seem to be genuine geological features. These seem to be reflected refractions originating from out-of-plane features. Given the noncontinuous appearance of these features the source could be fractured terminations of units. These reflectors were fractions could be removed or suppressed by using structural smoking or another filter, to enhance the background geology.

 In panel (b) the many faults are marked along wiggly reflectors that not necessarily represent horizontal discontinuities. Therefore the interpretation is not convincing.

 The author uses words such as "strongly curving", "pronounced symmetry", "steeply", "gently", "large", "minor", "major", "highly folded", "significant", "highly unlikely"…- all these words are qualitative descriptions trying to convince with the point. They are not convincing since they are not informative. It is better to present data and numbers to convince. Please correct this throughout the text. For example, what is the difference between curving and strongly curving? What is the actual dip of the flanks?

 The "interpretation" section of this manuscript should be the first part of the discussion as it compares and confronts the new interpretation with previous ones. The many citations in this section are in fact a discussion and not part of the "results".

 Please view the additional review comments in the file found here.

Is the study design appropriate and does the work have academic merit?

Yes

Is the work clearly and accurately presented and does it cite the current literature?

Partly

If applicable, is the statistical analysis and its interpretation appropriate?

Not applicable

Are all the source data underlying the results available to ensure full reproducibility?

Yes

Are the conclusions drawn adequately supported by the results?

Partly

Are sufficient details of methods and analysis provided to allow replication by others?

Partly

Reviewer Expertise:

Tectonics, seismic interpretation, marine geology and geophysics

I confirm that I have read this submission and believe that I have an appropriate level of expertise to confirm that it is of an acceptable scientific standard, however I have significant reservations, as outlined above.

# Response

**Published:** 2025-02-18

**Authors:** Jean-Baptiste Koehl

**Affiliations:** Geosciences, Universitetet i Oslo, Oslo, Oslo, Norway

Dear Prof. Schattner, thank you very much for your input on the manuscript, it is highly appreciated. Here is our reply to your comments. We hope the changes we implemented improve the shortcomings of the manuscript highlighted by your comments and suggestions. Please do not hesitate to contact us shall this not be the case for some comments. **Comments by the reviewer**

Comment 1: This interesting and well-written study challenges the prevailing paradigm regarding the De Geer Zone near Spitsbergen. The study includes nice illustrations that convey the author's message. The text and illustrations are addressed in the comments below and the annotated manuscript. In general, when the study challenges a widely accepted paradigm, it should first present that paradigm. Then the "cracks" in the paradigm should be presented and lead to the main questions that gave motivation to the present study. This should lead to the aim of the study at the end of the introduction.

Response: Agreed. Changes: Split the “Introduction” section into “The paradigm”, “Cracks in the system”, and “Goals of the study” sub-sections and added information from the discussion to provide a better overview of the main issue approached in the present manuscript and better highlight the main inconsistencies. Added three new paragraphs to the “The paradigm” sub-section: “It was first proposed based on the linear morphology of the northern coastline of Greenland and western Svalbard (e.g., De Geer, 1926; Holtedahl, 1936). Holtedahl (1936) notably described that the De Geer Zone is characterized by a series of submarine depressions west of Svalbard and the western Barents Sea extending into the fjords of northern Norway. Later on, a correlation was proposed between the southern offshore segment of the De Geer Zone, the Senja Fracture Zone, which runs along the edge of the southwestern Barents Sea margin (e.g., Myhre *et al.*, 1982), and the subvertical, late Paleoproterozoic, NW–SE-striking Senja Shear Belt onshore northwestern Norway ( Bergh *et al.*, 2010; Zwaan, 1995; Figure 1). Major NE–SW-striking post-Caledonian fault complexes seem to change polarity (i.e., dip direction) across the Senja Shear Belt, which was thus interpreted to have been reactivated as a post-Caledonian transfer zone in the late Paleozoic–Cenozoic (Indrevær *et al.*, 2013; Olesen *et al.*, 1993; Olesen *et al.*, 1997). Recent onshore studies have contributed evidence of strike-slip kinematics (e.g., slickensides) along post-Caledonian, NW–SE-striking brittle faults in the area, thus supporting the possible reactivation of the Senja Shear Belt as a strike-slip fault during the Cenozoic (e.g., Distelbrink, 2024). However, neither specific age constraints (e.g., geochronological analysis) nor precise estimates on the amount of strike-slip displacement accommodated by NW–SE-striking were obtained. Farther inland, the De Geer Zone and the Senja Shear Belt are believed to link up with the Bothnian–Senja Fault Complex, a presumed Proterozoic fault which extends to the Gulf of Bothnia (Henkel, 1987; Henkel, 1991; Indrevær *et al.*, 2013). Later studies argued that Svalbard and northern Greenland must have been adjacent to one another prior to the Cenozoic based on similar rock units and tectonic events (e.g., Harland *et al.*, 1993; Majka *et al.*, 2021; Piepjohn *et al.*, 2016). Examples of tectonic events correlated on both margins include the presumed Late Devonian Ellesmerian–Svalbardian Orogen and the early Cenozoic Eurekan fold-and-thrust belt ( McClelland *et al.*, 2021; Piepjohn *et al.*, 2016; Tessensohn & Piepjohn, 2000). Harland *et al.* (1993) also established regional correlations of Precambrian rock units in northern Greenland and Svalbard, which were claimed as evidence of long-lived close proximity between the two regions. A study of seismic reflection data along the western Barents Sea margin by Faleide *et al.* (1993) gave weight to the De Geer Zone hypothesis. The study notably suggested major dextral strike-slip movements along the Senja Fracture Zone and related faults such as the Bjørnøyrenna Fault Complex ( Figure 1). Further plate tectonic modeling and paleogeographic reconstructions cemented the vision of the De Geer Zone by showing that the De Geer Zone hypothesis is plausible from a plate kinematic perspective (e.g., Faleide *et al.*, 2008; Shephard *et al.*, 2013).”. Also added three new paragraph to the “Cracks in the paradigm” sub-section: “The study by Faleide *et al.* (1993) provided no robust evidence supporting the model of the De Geer Zone they proposed. In addition, their study did not include the seismic reflection data they used, only interpretation sketches. Furthermore, the fault zones they suggested to be major strike-slip and/or transform faults, e.g., Senja Fracture Zone and Bjørnøyrenna Fault Complex ( Figure 1), show listric and moderately-dipping geometries (e.g., Gabrielsen *et al.*, 1997; Koehl *et al.*, 2023a), which are incompatible with large-scale strike-slip displacement. Moreover, movement along NW–SE-striking faults in Senja and Kvaløya is yet to be accurately constrained as, as up to now, no geochronological constraints are available for these faults. Geochronological studies of NE–SW-striking brittle faults in the area (e.g., Davids *et al.*, 2013) and NW–SE-striking faults in northern Norway (Koehl *et al.*, 2018; Torgersen *et al.*, 2014) indicate that most brittle faults are late Paleozoic, which is not in line with previously proposed major strike-slip reactivation of onshore Precambrian shear zones and fault systems (e.g., Distelbrink, 2024; Olesen *et al.*, 1997). Furthermore, there is no trace of major faulting or indicators of large (tens to hundreds of km) strike-slip displacement along NW–SE-striking faults in northwestern Norway (e.g., Indrevær *et al.*, 2013; Koehl *et al.*, 2019). In addition, the Bothnian–Senja and Bothnian–Kvænangen fault complexes were initially inferred from elongated NW–SE-striking magnetic anomalies, which are now known to reflect basement features such as late Paleoproterozoic Svecofennian–Svecokarelian folds and greenstone belts (e.g., Henderson *et al.*, 2015; Koehl *et al.*, 2019), thus further casting doubt on the occurrence of major strike-slip movements in northwestern Norway.”. Also added the newly added references to the reference list.  

Comment 2: The introduction should also include the general tectonic map of the entire area, with the study area within it, otherwise, the global reader will not be able to evaluate the study. All the geographic and tectonic features mentioned in the text should appear in that figure. Response: Agreed. Changes: Added a new Figure 1a.  

Comment 3: Presenting the prevailing paradigm in the text and figure is important for the author in order to convince the reader that the paradigm should change. In this context, it is also important to add two additional figures – (1) a table with the commonly accepted geological history of the region (possibly separated into subregions). This table will also include a column with the new approach presented in the current transcript. (2) the second figure needed should include a sketch of the geological development of the region in 2-4 panels. This figure should appear in the discussion to support the text and show how the new interpretation fits into the regional tectonics. Response: Agreed. Changes: Added a new Figure 1a and a new Figure 9, as well as several paragraphs to the discussion to further expose the proposed change of paradigm: “The newly mapped Timanian thrust systems north and west of Svalbard ( Figure 1b, Figure 2, Figure 3, Figure 4, Figure 7) probably controlled the formation of currently active NW–SE-striking transform faults in the Fram Strait, the Spitsbergen and Molloy fault zones ( Figure 1a–b; Koehl *et al.*, 2021; Koehl & Mottram, 2024). A new tectonic model in which Timanian thrust systems accommodate most of the transform is proposed ( Figure 9a–b). As a result of sinistral movements along WNW–ESE-striking Timanian faults, N–S-striking Caledonian and Eurekan are reactivated as minor dextral strike-slip faults ( Figure 9b). This model is supported by the repeated reactivation of Timanian thrust systems as sinistral strike-slip faults in the Phanerozoic (e.g., Koehl, 2020; Koehl *et al.*, 2022a; Koehl *et al.*, 2023a; Mazur *et al.*, 2009; von Gosen & Piepjohn, 2001; Ziemniak *et al.*, 2022) and by indications of dextral movements along N–S- to NNE–SSW-striking faults onshore Spitsbergen (e.g., Bergh *et al.*, 1997; Harland & Horsfield, 1974; Maher *et al.*, 1997) and Prins Karls Forland (e.g., Lepvrier, 1990).

https://s3-eu-west-1.amazonaws.com/openreseurope/linked/247333.16791%2C_Author_response_to_Uri_Schattner_v1.pdf

**Figure 9. Schematic model of the interaction of WNW–ESE-striking Timanian faults and N–S-striking Caledonian and Eurekan faults in Svalbard during the opening of the Fram Strait.** ( a) Inherited, late Neoproterozoic Timanian thrust systems such as the Kinnhøgda–Daudbjørnpynten and Kongsfjorden–Cowanodden fault zones (KDFZ and KCFZ; red lines) are reactivated as sinistral strike-slip faults during oblique extension and localize the formation of major transform faults, the Spitsbergen and Molloy fault zones (SPZ and MFZ; dashed yellow lines). ( b) Preexisting NNE–SSW-striking (e.g., Caledonian and Eurekan) structural grain and fabrics are reactivated as local dextral strike-slip faults (orange lines) to accommodate tectonic adjustments during NW–SE-striking transform faulting. The green line shows the amount of dextral movement along N–S-striking faults, i.e., a few km up to 10 km, and the blue line sinistral movement along WNW–ESE-striking faults, which is in the order of a few tens of km. Abbreviations: IYF: Isfjorden–Ymerbukta Fault; KCFZ: Kongsfjorden–Cowanodden fault zone; KDFZ: Kinnhøgda–Daudbjørnpynten fault zone; MFZ: Molloy fault zone; SEDL: Svartfjella–Eidembukta–Daudmannsodden Lineament; SFZ: Spitsbergen fault zone. Extrapolating the schematic fault-block model from Figure 9 to Spitsbergen, the maximum amount of lateral movement along N–S-striking faults is in the order of 10 km. This is not cumulative because, in this model, N–S-striking faults are simply accommodating local tectonic adjustments in between major WNW–ESE-striking discontinuities in the crust (Timanian thrust systems) and are segmented (e.g., Hornsund Fault Complex west of Spitsbergen, which dies out north of Prins Karls Forland and south of the Risen fault zone; Figure 1b). Since the blocks in Figure 9 are schematic and rocks deform more complexly, i.e., partitioning deformation and distributing displacement along more structures, this is an upper estimate and dextral offsets of a few km are more likely. This is in agreement with estimates from previous studies along N–S-striking dextral faults in western Spitsbergen, e.g., N–S-striking Svartfjella–Eidembukta–Daudmannsodden Lineament in Oscar II Land ( Maher *et al.*, 1997; Figure 1b) and NNE–SSW-striking Isfjorden–Ymerbukta Fault ( Bergh *et al.*, 1997; Braathen *et al.*, 1999; Harland & Horsfield, 1974). This model does not require hundreds km dextral transform movements between Greenland and Svalbard. Restoring Greenland and Svalbard prior to the opening of the Fram Strait would thus come down to closing the oceanic crustal domain at the Knipovich Ridge (and possibly at the Molloy Ridge) along the two NW–SE-striking transform faults, the Spitsbergen and Molloy fault zones ( Figure 1a). Since new high-resolution magnetic data have shown that the oceanic crustal domain between Greenland and Svalbard is relatively narrow (c. 100–200 km; Dumais *et al.*, 2020; see ocean–continent boundary in Figure 1a), the first step would be relatively simple and involves matching the northern edge of the Kinnhøgda–Daudbjørnpynten fault zone shows in Figure 1b and Figure 4 with its counterpart at the Hovgård Ridge, where a comparable Timanian thrust was mapped on seismic reflection data ( Koehl, 2020; Figure 1a). To proceed with closing the Arctic Ocean and northeastern Atlantic Ocean, one would need to restore the Timanian Orogen in the Fram Strait to its original crustal thickness (> 40 km), while closing the remaining oceanic crustal domains at the Gakkel and Mohns ridges. As a result of the proposed restoration, the Svalbard Archipelago would have lain c. 200 km closer to Greenland prior to the opening of the Fram Strait, i.e., east rather than north of Greenland as suggested by previous correlations ( Harland, 1967; Harland, 1969; Jones *et al.*, 2016; Jones *et al.*, 2017; Majka *et al.*, 2021; Piepjohn *et al.*, 2016). This configuration is likely inherited from the Timanian Orogen and has, thus, likely persisted from the end of the Timanian Orogeny in the latest Neoproterozoic until mid–late Cenozoic extension. Jones *et al.*, (2016) have argued that the occurrence of thin volcanic ash layers probably erupted in Kapp Washington in northern Greenland and Ellesmere Island in Arctic Canada ( Figure 1a) in lower Cenozoic strata in central Spitsbergen suggested close proximity of Svalbard with these two volcanic centers. However, volcanic ash may travel over large distances (> 2000 km) and a single ash bed may cover broad areas. For example, the Lava Creek ash bed was erupted from Yellowstone in the Pleistocene and is found all over Texas and western Louisiana, i.e., up to 2300 km from the volcanic center ( Izett & Wilcox, 1982). This distance is larger than that between the volcanic centers in Ellesmere Island and northern Greenland and the ash layers in central Spitsbergen at present (respectively 1900 and 1200 km). In the present model, Svalbard is interpreted to have been located some 200 km closer to both volcanic centers in the early Cenozoic, thus further reducing the distance of Svalbard with the volcanic centers. The proposed model is therefore in agreement with an origin in northern Greenland and Ellesmere Island for the ash layers in central Spitsbergen.”. Finally, added a new Figure 10 showing the tectonic evolution of the transform margin. Comment 4: Below are some comments in addition to the annotated manuscript. Figure 1 should present a more general location map with the tectonic features, for the global reader. Response: Agreed. Changes: Added a new Figure 1a. Comment 5: The introduction should end with a paragraph or two summarizing the previous paradigm, the open questions regarding that paradigm which are the motivation for present study, the aim of the present study. Part of this appears in the second and third paragraphs of the introduction and should be moved to the and of the introduction. Response: Agreed. See response to comment 1. Changes: See response to comment 1. Comment 6: Since this paper attempts to change the prevailing paradigm regarding a major fault zone, the author may consider presenting a figure that will show the previous interpretation of faults on a map and a seismic profile used in previous studies showing the De Geer Zone. Without these, the global reader who sees these structures for the first time will have difficulties in evaluating the new perspective of the present study. The current version jumps immediately to the author's interpretations. Response: Agreed. Changes: Added new Figure 1a, Figure 7, and Figure 8. In addition, and split the “Description” section into “ ***WNW–ESE- to E–W-striking structures***” and “ ***N–S- to NNW–SSE-striking structures***” sub-sections and added a description sub-section and interpretation for the newly added Figure 7 and Figure 8: “ ***N–S- to NNW–SSE-striking structures*** E–W-striking seismic cross sections west of Albert I Land in northwestern Spitsbergen show a series of four N–S- to NNW–SSE-striking basins and highs, three of which are fully covered by the data ( Figure 7). The eastern basement high shallows up to near sea level and seismic reflections there are challenging to interpret due to multiples ( Figure 7). Similarly to the south-dipping packages described in the previous sub-section, basement rocks at depths of 1.5–6.0 seconds (TWT) in the two central highs consist of a c. 50 km wide system of numerous moderate-amplitude, curving, asymmetric reflections and associated linear, moderately-dipping disruption surfaces. Both the curving asymmetric reflections and disruption surfaces are dominantly found on the western flank of the two central basement highs ( Figure 7 and Figure 8). There, the curving reflections lean dominantly to the east and the disruption surfaces dip moderately to the west, which roughly parallel the irregular Top-basement reflection ( Figure 7). Some curving reflections with opposite geometries (i.e., leaning towards the west) are observed locally on the eastern flank of the two central basement highs ( Figure 7). The data also show a few small packages of Z-shaped reflections in between major disruption surfaces ( Figure 7). The westernmost basement high displays a rugose Top-basement reflection and is relatively challenging due to a relatively chaotic facies related to noise artifacts, which appear both in basement rocks and overlying sedimentary successions in the western part of the seismic transect ( Figure 7). Nevertheless, some asymmetric curving reflections and disruption surfaces are present ( Figure 7).

https://s3-eu-west-1.amazonaws.com/openreseurope/linked/247333.16791%2C_Author_response_to_Uri_Schattner_v1.pdf

  Figure 7. ( **a**) Interpreted and ( **b**) uninterpreted E–W-oriented seismic section showing the west-dipping geometry of the Hornsund Fault Complex northwest of Spitsbergen, its relationship to probable top-east Caledonian mylonitic thrust surfaces, and its influence in the formation of post-Caledonian basins and faults. The vertical black line in the data indicates a change of dataset (i.e., intersection of two seismic lines). Location is shown in Figure 1b. Abbreviation: HFC: Hornsund Fault Complex; SB: Sjubrebanken basin.

https://s3-eu-west-1.amazonaws.com/openreseurope/linked/247333.16791%2C_Author_response_to_Uri_Schattner_v1.pdf

 Figure 8. ( **a**) Interpreted and ( **b**) uninterpreted zoom in the upper part of the Hornsund Fault Complex consisting of asymmetric, east-verging folds and mylonitic surfaces probably of Caledonian age, and its relationship to the overlying post-Caledonian Sjubrebanken basin. Notice the syn-tectonic (upper Paleozoic?) sedimentary sequences in the lower half of the basin (green lines) and their erosional truncation above a basement high to the west (toplaps symbolized by white half arrows) and onlapping syn-Eurekan early Cenozoic and post-tectonic late–mid-Cenozoic sequences (blue lines). See legend in Figure 7. Of the basins overlying basement rocks in this area, the easternmost displays an U- to V-like shape. Intra-basinal reflections can be traced for tens of km and are gently curved downwards into a several tens km sag ( Figure 7). While most intra-basinal reflections onlap adjacent basement rocks on the basin flanks, sedimentary units are thicker in the basin center and pinch out towards the basin flanks. In the west, this basin is truncated by a steeply east-dipping disruption surface between depths of 2.0–4.5 seconds, which terminates into basement rocks (TWT). The central basin is asymmetric and intra-basinal sedimentary units can be mapped in great detail ( Figure 8). This basin is hereby named Sjubrebanken basin. The lower reflection packages consist of dominantly continuous, gently to steeply east-dipping reflections and thicken eastward towards disruption surfaces localized along the Top-basement reflection (green lines in Figure 7 and Figure 8). In the west, the lower reflection packages steepen and terminate as toplaps truncated unconformably by overlying sedimentary units ( Figure 8). Most of the upper reflection packages can be traced throughout the entire seismic section and display gentle thickness variations, generally thickening towards the center of basins (blue lines in Figure 7 and Figure 8). In both lower and upper reflection packages, a few curving reflections and small disruption surfaces are found ( Figure 8). The westernmost basin only partly shows in the data and consists of westward-thickening reflection packages ( Figure 7). Locally, high-amplitude positive reflections with U- and X-shaped geometries crosscut reflections in the lowermost part of the basin ( Figure 7).” Together with a few minor modifications of the first three paragraphs of the “Interpretation” section, added a new fourth paragraph: “The west-dipping disruption surfaces west of Albert I Land show a comparable truncate east-verging asymmetric folds and duplexes ( Figure 7). They are therefore interpreted as east-verging, possibly mylonitic thrusts. Their dominant vergence to the east is comparable to that of Caledonian thrusts onshore western Spitsbergen (e.g., Ohta *et al.*, 1986; Ohta *et al.*, 1995). Thus, they are interpreted as Caledonian thrusts and shear zones.” and several paragraphs near the end of the section: “Asymmetric folds also occur in sedimentary rocks west of Albert I Land where they verge to the east, except in the uppermost three sedimentary packages (i.e., up to the grey blue reflection in Figure 7 and Figure 8). Since the only episode of post-Caledonian contraction known in Svalbard is the early Cenozoic Eurekan event, these east-verging folds and related minor brittle thrusts are interpreted to have formed during this event. Thus, sedimentary successions above the grey blue reflection in Figure 7 and Figure 8 are interpreted as mid–late Cenozoic (probably post-Eocene) strata. The lower reflection packages in the asymmetric sedimentary basins west of Albert I Land (e.g., Sjubrebanken basin) typically thicken towards disruption surfaces localized along the flanks of basement highs. These are interpreted as syn-tectonic sedimentary strata deposited along half-graben-bounding normal faults ( Figure 7 and Figure 8). In the Sjubrebanken basin, these syn-tectonic deposits steepen to the west where they are erosionally truncated at a high angle above the adjacent basement high ( Figure 8). This abrupt termination and the absence or thin character of sedimentary deposits of the lower three sedimentary packages of the upper sequences (i.e., between the light green and common blue reflections in Figure 7 and Figure 8) suggests that the basement high directly west of the Sjubrebanken basin was exhumed during the early–mid Cenozoic. The fact that the lower two successions of the upper sedimentary sequences were folded during the Eurekan event but were not deposited onto this basement high and onto tilted and eroded lower sedimentary sequences indicates a relationship between exhumation, folding, and sedimentation and a hiatus with underlying sedimentary packages of the lower sequences ( Figure 7). It is thus suggested that the lower two folded successions of the upper sedimentary sequences were deposited during early Cenozoic Eurekan contraction (i.e., Paleocene–Eocene). In contrast, the strata of the lower sequences are bounded by normal faults and are thus interpreted as possible upper Paleozoic, collapse-related strata (probably Devonian–Carboniferous). This is supported by the reactivation–overprinting of preexisting, basement-seated Caledonian thrusts and shear zones by the interpreted late Paleozoic, half-graben-bounding faults ( Figure 7 and Figure 8). It is possible that Devonian (and/or) mid Cenozoic core complex exhumation played a minor role exhuming the basement high and overlying upper Paleozoic strata, as noted for other basement culminations onshore northern and western Spitsbergen (e.g., Braathen *et al.*, 2018; Schaaf *et al.*, 2021). However, there is no trace of typical, core-complex-related, bowed mylonitic detachment near the Top-basement reflection below the upper Paleozoic sedimentary strata. Further work is therefore needed to establish whether such processes were at work or not. By contrast, evidence of Eurekan folds and thrusts in lower Cenozoic sedimentary strata indicate Eurekan contraction as a major driver for exhumation ( Figure 7 and Figure 8). The easternmost basin west of Albert I Land is not bounded by any major tectonic structure. The V-like geometry of the lowermost part of the basin suggests that basement rocks were eroded possibly by glaciers and/or fluvial systems and that the basin was then passively filled by sediments. It is possible that a normal fault bounds the eastern flank of this basin, the interpretation of which is complicated by multiples in the easternmost basement high ( Figure 7). However, the eastward pinching-out character of the sedimentary rock units in this basin suggests that this is not the case and that the basin simply subsided. It is also possible that an east-dipping normal fault along the Top-basement reflection bounds the basin to the west. However, the lowermost basin strata rather thicken towards the basin center ( Figure 7). Core-complex-related detachment faulting is therefore unlikely for this basin. The absence of any major structure in the easternmost basin indicates that the only possible candidates to correspond to the Hornsund Fault Complex are the moderately west-dipping normal faults on the western flank of the two central basement highs ( Figure 7 and Figure 8). None of them is steep enough to have accommodated significant strike-slip movement and both are relatively minor faults extending for only a few tens km ( Figure 1b). In the westernmost basin west of Albert I Land, the high-amplitude positive reflections with X- and U-shaped geometries truncate both basement rocks and upper Paleozoic sedimentary rocks ( Figure 7). The high-positive contrast in acoustic impedance suggests the occurrence of relatively denser, possibly mafic igneous rocks. Their singular X- and U-shaped geometries are typical of saucer-shaped sills, which are common among Early Cretaceous intrusions of the High Arctic Large Igneous Province in central and eastern Spitsbergen (e.g., Senger *et al.*, 2013). The sills are thus interpreted to be Early Cretaceous. It is also possible that they are in fact mid–late Cenozoic and related to the rifting of the Fram Strait. However, igneous rocks related to this tectonic event are scarce and only a few occurrences of lava flows and plugs have thus far bee reported onshore northern Spitsbergen (e.g., Amundsen *et al.*, 1987; Burov, 1965; Gjelsvik, 1963; Griffin *et al.*, 2012; Hansen *et al.*, 2003; Skjelkvåle *et al.*, 1989). It is therefore more probable that the sills are Early Cretaceous in age.”. Furthermore, added “The present study places the Hornsund Fault Complex c. 50 km west of the coastline of Albert I Land in northwestern Spitsbergen ( Figure 7 and Figure 8). However, this fault and other nearby N–S- to NNW–SSE-striking faults and basins (e.g., Sjubrebanken basin) extend only a few tens km prior to dying out north of Prins Karls Forland and south of the Risen fault zone ( Figure 1b).” at the end of the first paragraph of the “Implications for the De Geer Zone and plate tectonics reconstructions” section of the “Discussion” chapter. Also split the second paragraph of the same section into two and rewrote the second part into “This is supported by the listric, moderately dipping geometry of the segment Hornsund Fault Complex west of Albert I Land ( Figure 7 and Figure 8) and elsewhere west of Svalbard ( Austegard *et al*., 1988; Eiken, 1994; Geissler & Jokat, 2004). Notably, Austegard *et al*. (1988) reported that all the structures west of Svalbard are extensional and that there are only very few occurrences of strike-slip movements. It is also supported by the lateral disconnection and/or segmentation of the Hornsund Fault Complex west of Svalbard as shown by the limited (a few tens km) N–S extent of the segment mapped west of Albert I Land ( Figure 1b). In addition, the only sparse evidence potentially indicating lateral movement is conflicting. For example, the possible sinistral strike-slip sense of shear indicated by right stepping geometries of margin-parallel brittle faults ( Eiken & Austegard, 1987) contrast with the major component of dextral strike-slip tectonics required for the commonly proposed sheared/transform margin model of the De Geer Zone ( du Toit, 1937; Faleide *et al*., 1993; Harland, 1961; Harland, 1967; Harland, 1969; Horsfield & Maton, 1970; Lepvrier, 1990; Lepvrier & Geyssand, 1985; Steel & Worsley, 1984; Steel *et al*., 1981; Steel *et al*., 1985; Wegmann, 1948). This is consistent with our interpretation of a minor extent and general lack of lateral movement along N–S-striking structures and with that of most previous offshore studies along the western Barents Sea–Svalbard margin (e.g., Eiken, 1994; Riis & Vollset, 1988).”. Finally, rewrote the first paragraph of the “Conclusion” chapter into “Two several kilometers wide south- and NNE-dipping shear zones of probable late Neoproterozoic age, the Risen and Kinnhøgda–Daudbjørnpynten fault zones, extend past the presumed location of the De Geer Zone west of Spitsbergen. The shear-zone geometries and kinematics are consistent with a formation during the Timanian Orogeny. Both fault zones are continuous and do not show any trace of lateral offset. In addition, the fault segments of the presumed De Geer Zone west of Spitsbergen developed along inherited, moderately west-dipping Caledonian thrust systems and show exclusively normal kinematic indicators, minor Eurekan contractional reworking, and limited, tens of km extent inconsistent with hundreds of km transform movements. Thus, the De Geer Zone does not exist and the faults presumably associated with the De Geer Zone accommodated dominantly vertical movements. The present results therefore suggest major revisions to all current Phanerozoic paleogeographic reconstructions for Arctic regions.”. Comment 7: In Figure 2 - the names of the seismic profiles are very long. Please shorten the names to simple numbers and maybe add a conversion table. For example profile 1 will be MPD-MOFF-97…. What does the vertical black line on the profile mean? Does the figure show a competent profile? This should be mentioned in corruption as well as in the figure. Response: The seismic section names should stand as they are important for the replicability and reproducibility of the results. Adding yet another figure/table would only lengthen the manuscript and not add much. The location of the seismic section is shown in Figure 1b. The author of the present manuscript concedes that the meaning of the vertical black line in Figure 2 should be stated. It is already indicated in the figure with the names of the two seismic lines used, both below (a) and (b). Changes: Added “The vertical black line in the data indicates a change of dataset (i.e., intersection of two seismic lines).” to the caption of Figure 2. Comment 8: The "bolded bedding surfaces" are not convincing. Most, if not all, seem to be artifacts of wiggly reflectors common in those depths. Response: Disagreed. The present study highlights potential artifacts in the interpreted seismic data. The wiggly reflections are not artifacts. The reviewer is welcome to recommend any relevant literature regarding potential similar artifacts. The targeted wiggly reflections are interpreted in terms of structural trends and shear zone kinematics and are consistent with Timanian thrust systems in Svalbard and the Barents Sea (e.g., Koehl et al., 2022a, 2023b; Koehl and Mottram, 2024). Such wiggly reflections are not omnipresent in deep seismic reflection data and therefore are unlikely representing artifacts related to frequency loss with depth. These wiggly reflections typically occur in areas where major thrust systems and orogens are known in nearby onshore areas (Norway: Paleoproterozoic Svecofennian Orogen, late Neoproterozoic Timanian Orogen, early–mid Paleozoic Caledonian Orogen; Faroe Islands and Scotland: late Paleoproterozoic Laxfordian Orogen, early–mid Paleozoic Caledonian Orogen; Iberia: late Paleozoic Variscan Orogen; Newfoundland: early–mid Paleozoic Caledonian Orogen, late Paleozoic Alleghenian Orogen; Madagascar: late Neoproterozoic East African Orogen). The first study that established the meaning of such wiggly reflection is Koehl (2021), which correlated sheared uppermost Devonian–Mississippian coal measures of the Billefjorden Group in central Svalbard to their offshore equivalent. Thus far, key examples have been interpreted in the Barents Sea (Koehl et al., 2022a), including cutting-edge 3D seismic reflection data tied to exploration wells from the energy industry (Koehl et al., 2023b) and 2D–3D data tied to gravimetric, magnetic, bathymetric data and onshore outcrops (Koehl and Stokmo, 2024), around the Faroe Islands (Koehl et al., 2024), in the Greenland–Iceland–Faroe Ridge (Koehl et al., 2025), and new examples have been identified by the author of the present manuscript in data published by other groups, i.e., in Jan Mayen (Blischke et al., 2022), in the Davie Ridge (Klimke et al., 2018), off Iberia (Lymer et al., 2019), and off Newfoundland (Keen et al., 2014). This is a rapidly developing topic with ground-breaking implications, one of which being that the De Geer Zone does not exist and that the fault segments interpreted as major paleo-transform faults (Hornsund Fault Complex, Knølegga Fault, and Senja Fracture Zone) are instead and have always been dominantly normal faults. The author of the present manuscript concede that this could be better outlined in the “Method” chapter. Changes: Added two paragraphs to the “Methods” chapter: “Notable structures interpreted include asymmetric folds within mylonitic shear zones and brittle–ductile thrust systems, which were first described by Koehl (2021) in central Svalbard. This study correlated uppermost Devonian–Mississippian coal measures of the Billefjorden Group sheared during early Cenozoic Eurekan contraction to their offshore equivalent. The onshore–offshore tie was facilitated by the relatively lower density of coal measures, which appear as bright negative high-amplitude reflections in seismic reflection data. In addition, they could be traces along the same fault zone (Balliolbreen Fault segment of the Billefjorden Fault Zone). Further studies of 2D–3D seismic reflection data and ties to exploration wells, gravimetric, magnetic, and bathymetric data as well as onshore outcrops have confirmed the character of asymmetric folds in seismic reflection data ( Koehl *et al.*, 2022a; Koehl *et al.*, 2023a; Koehl *et al.*, 2023b; Koehl *et al.*, 2024: Koehl *et al.*, 2025; Koehl & Stokmo, 2024). Asymmetric folds in seismic reflection data typically appear as moderate-amplitude upward-curving reflections with asymmetric flanks, including a relatively long and gently-dipping flank and a narrower and steeper flank. The narrower and steeper flank indicates the direction of tectonic transport (sense of shear). Other important and related structures include mylonitic shear surfaces. These have been known for some time and typically appear as bright, high-amplitude, moderately- to gently-dipping planar reflections (e.g., Fountain *et al.*, 1984; Hurich *et al.*, 1985; Phillips *et al.*, 2016; Fazlikhani *et al.*, 2017), owing their high positive amplitude to the relatively high density of mylonite ( Bell & Etheridge, 1973; Sibson, 1977).”. Also added the new references to the reference list. Comment 9: In order to support the author's interpretation, it would be useful to mark names on the interpreted fault systems. Response: Agreed. Changes: Added the names of the fault zones in Figures 2–4 and to the figure captions. Comment 10: It is not clear what the dashed white lines represent. Response: Agreed. These are artifacts and they are missing from the legend. Changes: Added the color scheme for seismic artifacts to the legend in figure 2. Comment 11: In general, the red and black markings of faults seem like synthetic and antithetic faults bounding a graben that was subsequently inverted, as evident by the compression structures on the black faults. Response: Disagreed. The black-colored reverse faults are far too gently-dipping to have initiated as normal faults. They can therefore not bound an extensional graben. The different coloring used for the faults symbolize the dominantly brittle versus ductile character of the interpreted structures.. Changes: None. Comment 12: Figure 3 - same comment about the wiggly yellow lines. Response: Disagreed. See response to comment 8. Changes: See response to comment 8. Comment 13: The undulating red horizons are presented as "Mylonitic shear surfaces". These horizons seem more like large folds. Response: Disagreed. This is the along-strike trace/expression of the south-dipping thrust system, which is tied to the N–S-trending seismic sections. Changes: None. Comment 14: Please see the suggested additions to the deputation of the profiles in figures 2 and three. Response: See response to comments 9 and 10. Changes: See response to comments 9 and 10. Comment 15: Figure 5 - Please separate the interpreted from the uninterpreted profiles Response: Disagreed. The format is the same as figures 2–4 in order to be consistent. Changes: None. Comment 16: In panel (a), the faults are marked along reflectors that do not seem to be genuine geological features. These seem to be reflected refractions originating from out-of-plane features. Given the noncontinuous appearance of these features the source could be fractured terminations of units. These reflectors were fractions could be removed or suppressed by using structural smoking or another filter, to enhance the background geology. Response: Agreed in that the faults are not continuous reflections but rather correspond to disruption (dislocation) surfaces likely juxtaposing different rock units, i.e., “fractured terminations of units” as suggested by the reviewer. In addition, the asymmetric fold reflections (yellow lines) are within the data vertical and horizontal resolution and are, thus, probably genuine. In addition, they are consistent with the occurrence of major disruption surfaces (i.e., shear/thrust surfaces; red lines). Hence, these reflections can no longer be ignored. Changes: See newly added information about the resolution of the seismic reflection data in the “Methods” chapter. Also added “Major fault surfaces (including mylonites) generally disrupt seismic reflections and corresponding rock units. However, faults do not necessarily show as through-going disruption surfaces, and some seismic reflections may locally appear undisrupted or only mildly affected. This may occur in areas where the fault rock (e.g., mylonite) is not sufficiently developed and where the rock units on either side of the fault have comparable density and seismic velocity, which may thus not produce a sufficient acoustic impedance contrast.” to the “Methods” chapter. Comment 17: In panel (b) the many faults are marked along wiggly reflectors that not necessarily represent horizontal discontinuities. Therefore the interpretation is not convincing. Response: Agreed. However, should a fault/shear zone juxtapose two units with similar density and seismic velocity and should the fault rock (e.g., mylonite) not be sufficiently developed (e.g., too thin), there should not be any contrast in acoustic impedance and the two units may appear as one single and continuous reflection despite being truncated by a fault/shear zone. The key factors here are (1) the alignment of discontinuities (i.e., truncated reflections) along the south-dipping disruption/dislocation surfaces and (2) the wiggly features representing asymmetrically folded units in between (mylonitic?) shear/fault surfaces. Changes: Added “The key to interpreting fault zones is thus the alignment of numerous disruptions/discontinuities (i.e., truncated reflections) and nearby related structures, which may also indicate proximity to a major fault.” to the “Methods” chapter. Comment 18: The author uses words such as "strongly curving", "pronounced symmetry", "steeply", "gently", "large", "minor", "major", "highly folded", "significant", "highly unlikely"…- all these words are qualitative descriptions trying to convince with the point. They are not convincing since they are not informative. It is better to present data and numbers to convince. Please correct this throughout the text. For example, what is the difference between curving and strongly curving? What is the actual dip of the flanks? Response: Disagreed. First, the present manuscript does not contain the terms “highly folded” and “pronounced symmetry”. Second, the reviewer probably means that the terms used in the manuscript may give the impression that the author of the present manuscript is trying “to persuade” the reader (for “to convince” appeals to logical reasoning, which cannot not affected by terms such as “significant”, “highly”, and so on). Thus, the author of the present manuscript is open to deleting the terms “strongly” and “pronounced”. Third, the reviewer is suggesting that the author of the present manuscript adds the dip angle of every fold flank mapped, which would be counterproductive as all fold structures are different and none show exactly the same geometry and dip angle. Thus, the descriptive terms used (e.g., “gently dipping”, “steeper”, “large”, “minor”, “major”, etc.) are better suited for the purpose of the manuscript. The author of the manuscript also argues that “tight folding” is a well-established term used in structural studies to describe fold structures with high amplitude vs wavelength ratios. Fourth, the term “significant” is used only once throughout the manuscript in a context in which it means “enough”: “significant rheological contrasts”, i.e., meaning rheological contrasts high enough to create the described rugose geometry of the Top-basement reflection. It is thus recommended that the term should remain as such to avoid lengthening the sentence unnecessarily and potentially confuse the reader. Changes: Deleted “strongly” and rephrased “show a pronounced asymmetry” into “are asymmetric” in the second paragraph of the Result chapter. Deleted “highly” in the fourth and seventh paragraph in the “Interpretation” sub-section of the “Results” chapter. Comment 19: The "interpretation" section of this manuscript should be the first part of the discussion as it compares and confronts the new interpretation with previous ones. The many citations in this section are in fact a discussion and not part of the "results" Response: While the author of the present manuscript concedes that the alternative proposed by the review is logical and makes sense, the current location of the “Interpretation” sub-section in the “Results” chapter is far more common in studies interpreting seismic reflection data (e.g., Phillips et al., 2016; Fazlikhani et al., 2017; Koehl et al., 2022a, 2023b) since the interpretation of the data is a result that is discussed in the “Discussion” and should, thus, precede the “Discussion” chapter. Changes: None. Comment 20: Reduce repetitions. Response: The targeted section is a mandatory section (Plain language summary). Changes: None. Comment 21: Please add a more general figure 1 for the global reader. Mark all the geographic and tectonic locations mentioned in the text. Also mark the location of the seismic data so the reader will be able to evaluate the location relative to the elements discussed in the text. Response: Agreed. However, the location of the seismic lines shown in Figures 2–4 is already displayed in Figure 1b. Changes: Added a new regional Figure 1a. Comment 22: Please show these features clearly on a map. Response: These structures are displayed in Figure 1b, which should be referred to in the present sentence. Changes: Added reference to Figure 1b in the targeted sentence. Comment 23: several – how many? Response: Several means more than two, i.e., at least three or more. Specifically, the Kinnhøgda–Daudbjørnpynten fault zone appears in at least 4 seismic sections and the Risen fault zone in at least 6 seismic lines. However, they probably also appear on seismic lines not yet available to the author of the present manuscript. Thus, remaining general and using “several” avoids overcrowding an already long sentence. In addition, it does not matter whether a specific fault appears on 4 or 5 seismic lines. The important thing is that each fault/shear zone appears on at least three lines including two cross sections and an along-strike section to confidently interpret its strike and 3D geometry. Changes: None. Comment 24: which seismic lines? Previously analyzed or presented in this study? Response: Agreed. Changes: Added “analyzed in the present study ( Figure 2, Figure 3, and Figure 4)” to the targeted sentence. Comment 25: discussing the implication of the study before presenting the previous paradigm and the new results is premature. This section should go to the discussion. The introduction should present the common knowledge about the study area and the De Geer zone; and highlight the main inconsistencies in the previous interpretations. Response: Agreed. Changes: Split and rewrote the last paragraph of the discussion as follows to include the information from the Introduction: “The results of the present study suggest that all the current plate tectonics models for the opening of the Fram Strait should be updated with new fault lines and kinematics. The present study shows the danger of using mostly local onshore structural fieldwork in deeply eroded Arctic areas like Svalbard to resolve regional tectonic issues. Such biases are illustrated in Koehl and Allaart (2021), whose work shows that the Billefjorden Fault Zone, although representing a major tectonic discontinuity at a local scale (tens of kilometers long fault with hundreds of meter-scale displacement), does not represent a major regional tectonic boundary as previous thought (e.g., Harland, 1969; Harland *et al*., 1992). The study also calls for a serious reconsideration of all major faults inferred from indirect observations, generally as necessities to make up for paleogeographic reconstructions shortcomings, rather than observed on specific datasets. An example is the Wegener Fault, a thousand of kilometer-long sinistral strike-slip fault inferred between Ellesmere Island and northwestern Greenland in the Nares Strait, which was proposed solely based on the physiographic morphology of the area, i.e., the linear geometry of the Nares Strait and tentative lateral offset of rock units on either side of the strait ( Taylor, 1910). Convincing evidence from both geophysical datasets (e.g., gravimetric and aeromagnetic anomaly maps) and field mapping show that the bedrock continues across the Nares Strait with no apparent lateral offset and that the Wegener Fault does not exist ( Oakey & Chalmers, 2012; Oakey & Damaske, 2006; Oakey & Stephenson, 2008; Rasmussen & Dawes, 2011; see also further references and arguments in Gion *et al*., 2017). Despite overwhelming evidence against the Wegener Fault, field geologists continue to take its existence as a fact and use it to discuss incomplete and/or sparse field observations and interpretations (e.g., Gilotti *et al*., 2018; von Gosen *et al*., 2019). This calls for strengthened collaborations between geophysicists and field geologists. It also further highlights the importance of interdisciplinary studies when mapping and interpreting major faults. Interdisciplinary studies should include at least some regional (e.g., geophysical) datasets, which are becoming more broadly available and user-friendly, rather than exclusively local fieldwork data. It is necessary to establish a methodology for the classification of faults in order to clearly segregate beyond-reasonable-doubts faults observed directly on specific datasets, e.g., during fieldwork and/or on geophysical datasets (e.g., San Andreas fault – Crowell, 1979; Grant Ludwig *et al*., 2019; Molnar & Atwater, 1973; Huffman, 1972; – and Timanian thrusts systems in the Norwegian Barents Sea and Fram Strait – Klitzke *et al*., 2019; Koehl, 2020; Koehl *et al*., 2022a; Koehl *et al*., 2023a) from tentative faults (i.e., inferred and thus not directly observed on any specific dataset; e.g., Wegener Fault and De Geer Zone). For example, the latter may be called “lineaments” or “zones” rather than “faults”. In addition, it is necessary to clearly report the amount and nature of the uncertainty associated with (1) the interpretation of the involved datasets and (2) each individual fault. This especially includes data collected and observations made during fieldwork, whose interpretation is no less subjective than that of geophysical datasets.”. Comment 26: please provide more extensive information about the data set: how many profiles, acquisition parameters, processing parameters and stages, year of acquisition, year of processing. Response: The database is already included in the supplements submitted to DataverseNO and referred to as “Extended data” in the present manuscript. Changes: Added two paragraphs and a table to the “Methods” chapter with the surveys’ horizontal and vertical resolutions compared to the size of the studied structures: “The vertical resolution of the NPD-MOFF-90, NPD-MOFF-93, and NPD-MOFF-97 surveys is ¼ of the wavelength and is computed from the velocity of basement rocks in the study area (6300 m.s ^-1^; Gernigon *et al.*, 2018) and frequency of the data (low-cut filter at 5 Hz and frequencies up to 40 Hz; Kristiansen, 1999). An estimate of the vertical resolution at high depth (using 40 Hz) is therefore c. 39 m (and minimum 315 m using the 5 Hz low-cut filter frequency). The bin size of the surveys is 12.5–25 m x 25 m (cable group length and shot point interval; Kristiansen, 1999), thus yielding a horizontal resolution of c. 313–625 m ( Table 1). The horizontal resolution of the surveys at a depth of 5000 m using a frequency of 40 Hz is c. 627 m ( Table 1). The structures studied include > 500 m wide and > 150 m thick asymmetric folds, which are within the horizontal and vertical resolution of the data, both at shallow and high depth (≥ 5000 m; Table 1). The vertical resolution of seismic data may be down to 1/32 of the wavelength in places ( Kallweit & Wood, 1982; Li & Zhu, 2000), i.e., down to c. 5–39 m for 40 Hz and 5 Hz frequency (= 6300/40/32), thus further supporting the interpreted structures. The water depth in the study area around Svalbard is about 200–400 m with little variations ( Jakobsson *et al.*, 2012) and, thus, has little influence on the interpreted structures. Seismic velocities for basement rocks in northern Norway from Gernigon *et al.* (2018) suggest that there is little to no vertical exaggeration in the basement rock interval ( Figure 2, Figure 3, and Figure 4). Thus, the geometry of the interpreted basement-seated structures is likely similar to their actual geometry. **Survey/structures** **Horizontal resolution (0 m/5000 m depth)/width** **Vertical resolution (5 Hz/40 Hz)/height** **Asymmetric folds** > 500 m > 150 m **NPD-MOFF-90/93/97** 312.5–625 m 627.495 m 315 m 39.375 m **Svalex** 312.5 m  m  m  m **Table 1:** **C** **omparison** **of** **the size of investigated structures** **with the h** **orizontal (for depths of 0 and 5000 m and using a frequency of 40 Hz) and vertical resolution (for frequencies of** **5** **a** **nd 40** **Hz and at a depth of 5000 m) of the seismic datasets used.**”. Comment 27: if the data was analyzed by one software there is not need to mention other software. Response: Disagreed. This is the policy of the journal and the European Union to ensure reproducibility and replicability. Changes: None. Comment 28: please avoid repetitive phrasing throughout the text. Response: Agreed. Changes: Deleted “previously”. Comment 29: it is preferred not to add citations in the result section. The results should be a product of the present contribution only. Previous results should appear in the introduction and/or the discussion. Response: Agreed. However, this is simply a citation of the dataset attached to the present manuscript. The dataset consists of high-resolution versions of the figures, which are necessary for the reader to explore the seismic reflection data in detail. Thus, this citation should be allowed. Changes: None. Comment 30: what are Z-shaped reflections? Response: These are reflections with curving geometries comparable to that of the letter “Z”. Changes: None. Comment 31: cross sections or lines? Please keep consistency throughout the text. Response: The terms suggested by the reviewer are not equivalent. A seismic line may refer to any 2D seismic section, whereas a seismic cross section is crossing a specific feature and is therefore a cross section relative to that specific feature. In the present case, the seismic line mentioned is a seismic cross section of the faults and shear zones discussed because it crosses them at nearly a right angle. Changes: None needed. Comment 32: “southward-leaning” – why using quotation marks? Is this true or not? Response: Agreed. Changes: Deleted the quotation marks in five occurrences. Comment 33: this map shows many abbreviations for structures that were interpreted in the seismic data. Please mark these abbreviations on the relevant seismic profiles as well. Currently it is difficult to compare the interpretation on the map with the profiles. Response: Agreed. Changes: Added the names of the structures discussed in the seismic sections. Comment 34: What is a “disruption surfaces”? Response: A disruption surface is a surface/plane along which seismic reflections are generally disrupted. In the present case, it is a descriptive term for mylonitic shear zones and thrust faults. Changes: Added “(i.e., planes/surfaces along which seismic reflections are generally disrupted)” to the first occurrence of “disruption surface” (i.e., in the first paragraph of the “Results” chapter). Comment 35: when trying to support one’s interpretation it is better to cite other people’s work. Response: While the author of the present manuscript concedes that such practice would be more objective, it cannot be applied in the present situation. To the knowledge of the author of the present manuscript, no other team or individual has yet picked on the discovery of the seismic facies of asymmetric folds and major thrust systems by the author of the present manuscript, i.e., the discovery that the wiggly reflections correspond to asymmetric folds and horses in duplex structures. The discovery and tie to equivalent onshore structures was established in Koehl (2021), a study in which the author of the present manuscript tied folded and sheared Mississippian coal measures onshore central Spitsbergen to their offshore equivalent on seismic reflection data (Koehl, 2021 figures 4a–b and 5a–b). The work was expanded upon by the author of the present manuscript to entire hundreds of km long 2D seismic reflection transects (Koehl et al., 2022a) and to 3D seismic reflection data (Koehl et al., 2023b; Koehl and Stokmo, 2024) in which the author of the present manuscript interpreted thousands of km long, tens of km thick thrust systems in the entire Barents Sea, some of which visualized in 3D and penetrated by exploration wells (e.g., Koehl et al., 2023b), others tied to ongoing earthquake sequences (Koehl, 2020; Koehl et al., 2024 in prep.). The author of the present manuscript concedes that many other scientists have worked on mylonitic shear zones on seismic reflection data (e.g., Fountain et al., 1984; Hurich et al., 1985; Phillips et al., 2016; Fazlikhani et al., 2017; Collanega et al., 2022), but no one has yet picked on his recent work on asymmetric folds. The only study that does exception to this rule is a study by Morley et al. (2017), which shows a simple antiformal thrust stack structure in their figure 8 and relatively large asymmetric folds associated with thrusting in their figure 4. However, the structures these authors mapped are relatively mildly deforming compared to the structures investigated in the present study, and these authors did not go further and interpret any of the related (smaller-scale) wiggly reflections as asymmetric folds. In addition, the examples they present are from New Zealand and Indonesia and can therefore not be cited in the present sentence, which targets the Barents Sea. Changes: None. Comment 36: Presenting a more general tectonic map in the manuscript would enable the global reader to identify these structures and assess their alignment with the proposed interpretation. This general map is crucial as one of the final figures that would show how the new interpretation fits into the context of regional tectonics. Response: Agreed. Changes: Added a new Figure 1a.
